# Supplementary material for: Coping with drug resistant tuberculosis alongside COVID-19 and other stressors in Zimbabwe: A qualitative study
Source: PLOS Glob Public Health. 2023 Aug 7;3(8):e0001706. doi: 10.1371/journal.pgph.0001706 (PMC10406177; doi:10.1371/journal.pgph.0001706)
Supplement: S1 Text — (DOCX) [file pgph.0001706.s001.docx]

**Talent**

35 year old woman who has been on ART for 6 years (since 2014). Now she is on second-line ART. She was diagnosed with extra-pulmonary MDR-TB and presented with a distended stomach which mimicked a heavily pregnant woman. Unemployed woman looking after 3 children. Husband moved out to a girlfriend when she was bedridden. She had to relocate to her mum’s place in search of a caregiver. Lost at least USD 1559 in terms of hospital bills, ambulance fees and car hire. Hospital bills are yet to be cleared.

Before treatment she was a part-time domestic worker earning around USD 25 per month. She used to get some goodies (food, soap) from employers. When the husband moved out, she assumed the role of head of household at a time when she needed a caregiver and was experiencing stigma financial haemorrhages. Around that time her skin turned darker (possibly due to clofazamine). Her youngest child (4th) was afraid of her. She has changed residency inorder to cope with the effects on DR-TB. She downsised the number of rooms she occupy.

____________________________________________________________________________

Collins: Let’s start our interview. I would to thank you so much for your time. Like what I said we are conducting this study so that we really understand your experiences with MDR-TB treatment and how you managed to stay on treatment and how you sought to maintain the livelihood of you households. We know that the treatment is very long and some people fail, some remain on treatment and recover. Let me start again by congratulating you for being able to stay on your treatment.

002: Uhh.

Collins: Uhh, so all the information we are gathering here shall be reported anonymously. Your name, where you stay or any other information that can identify you will not appear in the final reports so feel free. And I want to remind you again that we will be using this audio recorder is it.

002: Uhh.

Collins: Yes so you can raise your voice a bit so that it can be heard. So as the facilitator I will also take notes during the interview. Are there any questions before we start?

002: At the moment i do not have any question.

Collins: Can you explain to me how long you have been on treatment?

002: 2 years.

Collins: Alright, since you started the treatment let’s say the day you were diagnosed with TB have you ever changed residency? Are you still staying where you used to stay?

002: I changed.

Collins: What made you change?

002: When I got sick I could no longer afford to pay rentals for the 2 rooms I used to live in.

Collins: Alright.

002: So I moved to a 1 room, then I moved from the 1 room and went back to the 2 rooms.

Collins: So you moved from where you used to stay… [Interjection]

002: From where I lived, going somewhere else.

Collins: So at the moment who are you staying with?

002: I am staying with my children.

Collins: How many are they?

002: 4.

Collins: 4 children?

002: Uhh.

Collins: Alright, what do you know about MDRTB that you were told or what you know?

002: MDRTB I was taught that it’s a type of TB that is difficult to treat.

Collins: Uhh.

002: It may appear to have gone but it is said to be incurable. E-eh it takes a long time for the drugs to make you feel better than how you were feeling. I leant that it’s a disease that is treated with a lot of drugs and the drugs themselves are painful. E-eh I also learnt that even if its long term but if you just do everything you are told, as for me I didn’t just start with drugs only…

Collins: Uhh.

002: I also received some injections so it’s a long process but it just needs to be followed.

Collins: Alright how is it spread, have you been told about it too?

002: How it spreads?

Collins: Yes how does it spread?

002: Hoo how it spreads, yes we were taught that it spreads infected people exhale and others inhale; it travels through the air.

Collins Alright, what are the signs and symptoms of TB?

002: Most of the times coughing, chest pains, the one I had was that of the stomach.

Collins: Uhh.

002: Yes, it was in my stomach, I just felt my stomach tighten and swell

Collins: Wasn't it in the lungs?

002: It affected my stomach…

Collins: Ooh it went into…

002: It affected my stomach.

Collins: Alright, did you get any form of support or assistance from Non governmental organisations (NGOs) or other organisations or from your family so that you stay on treatment? Is there any form of support that you…?

002: I did not get support from any organization. I survived on a tight budget since our MDR tablets are given for free.

Collins: Uhh.

002: We just went to the hospital for treatment. The challenge began at the beggining when they start to notice there is a problem. There are some initial tests that you have to undergo. So those tests had to be paid for because they were done at Harare hospital until they discovered that I had MDR.

Collins: Uhh.

002: And the other challenge was when I was admitted at Harare hospital the admission bills were to be settled, the medicines that I took before I was diagnosed with MDR was paid for, admission at Wilkins hospital was paid for, the ambulance that transported me was paid for.

Collins: Uhh.

002: I think these are the challenges I faced because it took me a long time to be able to walk on my own since I was on injections. I travelled on a daily basis for injections that had been prescribed, I was on injections and they said I should tell the doctor when my ears start to have an irritating sound and he would withdraw the injections.

Collins: Uhh.

002: So I spend about 72 days on daily injections.

Collins: Uhh.

002: So during the first days, for almost 2 months I couldn’t walk on my own to the hospital, they would hire a vehicle to ferry me.

Collins: They had to hire a vehicle?

002: Uhh.

Collins: So how much did they charge you for a return trip?

002: The transport that I used… because when I was discharged from the hospital I went to Epworth… where I used to stay was far from the hospital. I used to stay in Overspill. The clinic that I went to is called Dombo raMwari and it’s far away from the clinic so I paid a dollar to and a dollar from the clinic.

Collins: Dollar nekombi?

Collins: Dollar for commuter omminbus?

002: For commuter omminbus I couldn’t … I had to negotiate [with car owner] because there was no commuter omminbus that proceeded to where I stayed, the area that I lived in was very far from the bus stop.

Collins: Alright so it was dollar to and dollar from… so it was a total of $2 a day?

002: I would spend USD$2 per day on transport

Collins: For how many days did you fork out this kind of money?

002: Uhmm for 2 months.

Collins: Paying dollar to and dollar from…?

002: From the clinic yes.

Collins: Why did you go to Epworth?

002: In Epworth I went to stay with my mother, the reason being I was looking for someone to assist me, someone who… because I couldn’t do anything for myself. I was bathed, sometimes I defecated on myself so I was monitored there. I wanted a caregiver to be aroud me every time.

Collins: Okay so that you have a caregiver?

002: Uhh.

Collins: What about the place you used to stay, who did u live with?

002: I lived with my husband and my children.

Collins: Where was he?

002: When I fell sick… I think it was difficult for him to accept my condition and then he looked for another wife. So he was….our relationship broke because of this disease.

Collins: Haa sorry about that. Alright let’s look at when… you once said you where admitted at Harare hospital is it?

002: Uhh.

Collins: Is that the only place where you where admitted?

002: No. At first when I fell sick I went to a clinic that is in the community that l lived in and then they referred me to Harare hospital. From [At] Harare hospital I was hospitalised for about a week.…

Collins: Were you bedridden?

002: Yes. I was bedridden.

Collins: Uhh.

002: So they were trying to figure out what the problem was.

Collins: They had not diagnosed you of TB yet?

002: They hadn't, they hadn't because they were worried about my stomach that kept on swelling. I coudn’t eat e-eh. I would cough but it wasn’t much. They first did a chest X-ray on me and they did not find anything and then they said the chest is normal.

Collins: Is normal.

002: They… they said one side on my lungs was not looking good but it was something small. So they did not know whether it was the lung that were causing my stomach to swell.

Collins: Your stomach.

002: Then they did another test on me. The test was very painful because the injection [needle] they used was thick and long. They inserted it into my stomach and then they extracted some fluid from there. They took it [fluid] to a private laboratory and that is when they detected that I had TB in my stomach.

Collins: Alright, so looking at… so you were only admitted at Harare hospital?

002: From [Harare hospital]… where I was diagnosed of MDRTB they said it will not be treated at Harare hospital because it is infectious and requires an isolation facility. So the isolation facility was available at Wilkins hospital. So I was moved from Harare hospital by an ambulance to Wilkins hospital where I was finally admitted for two months.

Collins: 2 months at Wilkins?

002: Uhh.

Collins: Alright. Looking at admissions at Harare hospital and Wilkins hospital as well as moving around in an ambulance who footed the bills, was it offered for free or…?

002: It was not for free; we paid. Infact I still have outstanding bills. I could only afford to pay small amounts since I was seriously ill. My condition did not allow people [hospital staff] to withhold health care service to me on the basis of failure to pay. So I still have outstanding bills. Some of the times I paid, but some of the times I failed to pay in full…

Collins: So how much was the bill at Harare hospital bill?

002: At Harare hospital I still remember if I am not mistaken during that time it was $60 if I am not making a mistake, I am not sure.

Collins: When was that?

002: In September.

Collins: Which year?

002: Let me check my records. Its unfortunate that I cannot find where I placed my initial treatment card.

Collins: The treatment when did you start your treatment?

002: This was 2018 December is it [looking at the treatment card].

Collins: Uhh.

002: So it was… I started [treatment] in October.

Collins: 2018?

002: Yes October 2018, because in October I was on injections. November I was on injections then December that is when I started…

Collins: So that costed you $60?

002: Uhh.

Collins: The conversion rate between the USD and Zimbabwe dollar was 1:1 is it not?

002: Uhh.

Collins: October 2018….the rate was 1:3, around 1:3.

002: But the official exchange rate was still 1 is to 1.

Collins: Yes so it was, so it was $60 per day?

002: Yes for one who is admitted.

Collins: Yes, so it was around USD 420, ambulance charges are around USD35. Uhh, So you were admitted for 2 months at Wilkins hospital, how much did they charge you per day?

002: Wilkins…how much was it….USD$16.

Collins: $16 per day?

002: It must have been $16.

Collins: OK. $16…

002: I am not sure about the other charges… I do not remember them.

Collins: Alright, let us proceed. So you first started to feel… you started to feel that your body was not well is it?

002: Uhh, my body was exhausted and drained.

Collins: So what did you do when you first experienced tiredness?

002: Those days when I started feeling unwell it was during the days of cholera [epidemic] and … what is it called? And typhoid…

Collins: Uhh.

002: Yes because where I lived there was an outbreak of cholera…the epidemic was devastating.

Collins: Outbreak.

002: There was an outbreak of cholera and typhoid. So I thought those might have been some of the symptoms of typhoid because I had a running stomach but I did not vomit.

Collins: Uhh.

002: I had running stomach and I felt drained and my stomach was swelling. So when I went to the clinic I was immediately put in the tents so that I could be screened for both cholera and typhoid. They collected and tested my stool and they said it was not typhoid or cholera.

Collins: Uhh.

002: So I went back home and took the tablets that they had given to me. When I returned home I spend about a week and I did not notice any change, the situation continued to [deteriorate]… my stomach continued to swell almost every day.

Collins: Uhh.

002: I ended up looking like I was pregnant.

Collins: Like a pregnant woman?

002: Yes, it had grown big to the extent that I was like someone who is pregnant. Anytime I ate any small quantity of foof, my my stomach would distend and continue to grow…

Collins: Uhh.

002: Yes, so I was in pain and the pain would extend to my ribs. So that is when I went back to the clinic and said though you treated me, but there is no change infact it’s getting worse.

Collins: Uhh.

002: That is when they referred me to Harare hospital. I didn’t manage to go on the very day that I was referred to Harare hospital because when I came… when I noticed… I got worse when I was at my elder sister’s funeral.

Collins: Uhh.

002: So when I returned [from my sister’s funeral], my husband did not want to take me to the hospital. So it got to a point when my brothers had to intervene and they threatened my husband with unspecified action in the event that my husband did not take me to hospital. That is when he took me to the hospital… he hired a car and took me to Harare hospital. Then…at Harare hospital that is where they started to do some investigations on me.

Collins: So how long did it take from the time you started to have the symptoms until…?

002: I think when I started to feel that this pain is now unbearable… [Interjection]

Collins: A-ah I mean… from the time you started to feel that your body was not well until the time you were diagnosed of TB, how long was that?

002: I remember when I was really sick its about 7 days, 7 days going to the clinic after a week of getting treatment for typhoid so it was about 2 weeks until I was diagnosed [with MDR-TB].

Collins: About 2 weeks?

002: Uhh.

Collins: Alright. So when you were diagnosed of MDRTB what came into your mind?

002: At the time when I was informed about the results I was fortunate because people told me that this was the situation I was in. Then they explained to me that MDR-TB was i) difficult treat ii) was contagious iii) and I was supposed to accept that I was suffering from MDR-TB. I didn’t… it didn't take me long to accept it because I was already on ART.

Collins: Uhh.

002: So I understood that a person who is in denial a condition may not attain the best health outcomes. So I just accepted it at that time but the thing that bothered me was that it [MDR-TB disease] was now different from how I was diagnosed [of HIV] and started on ART…

Collins: On ART...

002: An MDR-TB diagnosis is a much bigger challenge, e-eh most people are not allowed to get close to you.

Collins: Uhh.

002: E-eh when I was in the ward, I was the only one there. Visitors were not allowed to get close to me; they were to keep a distance when talking to me. So it… it affected me mentally, even when we were being treated there a-ah it was hard because we weren't treated inside as we were used to. When a person is on ART one can be close to his/her attending nurse and they can communicate with each other. It was very different with MDR.

Collins: Uhh.

002: With MDR we would get outside to get our medications. We used to go to a certain window to hand out our [treatment] cards. Then the injections were administered on something that was built and there was something that could be moved like those in the hospital.

Collins: Uhh.

002: That is what was available. They would take it outside of the clinic and the nurse would come and administer the injections. On rare occasions we would be served by a well mannered nurse….but in most cases we were served by nurses who were so scared to come close to us to administer the injection...It was difficult, a difficult moment. Because back then we there weren’t any masks that we are putting on nowadays.

Collins: Uhh.

002: We used put them on on a daily basis when we went for medical treatment, whilst at home we were encouraged to wear them so that we would not infect others.

Collins: How did they administer the injections?

002: A-ah they were actually stabbing us. The injection was painful I don’t want to lie. The injection was… one would be gripped with fear every morning at the thought of going back again…. Because the injection was given on a daily basis.

Collins: Uhh.

002: But once we got there……Not all of them but in most cases you would perceive that the nurses were also scared.

Collins: Did you tell other people that you had been diagnosed of DRTB?

002: Uhmm family?

Collins: Even family yes.

002: I disclosed to my family and even my friends within my community.

Collins: When you told them how did they receive it?

002: As for me yaa they accepted it, some accepted it but in my house there was a problem. My husband could not accept it immediately.

Collins: Alright, alright before you were diagnosed of MDRTB, when you were still fit before you started feeling sick e-eh how did you survive e-eh, what was your way of life.

002: I used to do part time jobs like cleaning houses, washing plates or accompanying some people’s children to school that is what I usually did: part time duties.

Collins: Alright, how much would you earn?

002: Yes I would earn $USD 25 a month.

Collins: Alright what about when you got sick?

002: I was not able to do anything.

Collins: Alright.

002: I was bedridden.

Collins: So at that time did it affect the food situation in your household?

002: It affected a lot of things?

Collins: What was affected?

002: It’s like my own children dropped out of school. They failed to go to school. One of them even refused to go to school. She was thinking “What if I came back and my mother is dead what would I do?” E-eh right now she was supposed to be in Form 2 but is in grade 7 because she refused to go to school. The other ones dropped out of school because I was not fit enough to be able to run around looking for jobs and assistance as their mother. I was bedridden and was also expecting help from people. So my children dropped out of school around that time.

Collins: Did your family members assist you, your uncles or other relatives?

002: The challenge I had was that my husband was previously married but the wife died. She left her 2 children and I was looking after those children. I want to thank God because those children helped me a lot, they were a grown up a bit.

Collins: They were a bit older?

002: Yes. The girl was in Grade 7. She would be chucked out of school because of failure to pay school fees but I was lucky because there was an organization called Mwana Wedu. Their staff came and paid her fees so that she could write the Grade 7 examinations but the other two failed to proceed with education. Getting food was a challenge.

Collins: Uhh.

002: But people from the community who came to pay me a visit would notice that I was still alive but had not eaten anything and they would identify the challenges I was going through…

Collins: Uhh.

002: And some of my neighbours, I remember even up to today that they used to bring me food. I realized that by disclosing my financial situation, and that I was on injections helped me a lot.

Collins: Uhh.

002: There was a time I decided to relocate from… I realized that I had run out of money for transport costs from Epworth to Domboramwari. But by then I was able get up and walk slowly on my own. Then I finished my course of tablets at the clinic that is in the surburb where I currently live.

Collins: Uhh.

Collins: Is the clinic close to your home?

002: Yes and I was now able to walk slowly [with some difficulties]. But when I took my tablets in the morning, and I was supposed to go for a clinic visit around 0900am, it followed that when I took 6 tablets [pills] I would fall into a deep sleep unless there was someone to wake me up. I would sleep. But my neighbours who live next door knew that I had informed that that I get daily injections at the clinic. So, it happens that whenever the time was close to 0900am……. I remember they would wake me up. They woke me up on three occasions reminding me to go [to the clinic] to get my injections. My neighbours would wake me up…

Collins: Your neighbours would wake you up……..

002: My neighbours would remind me that it was almost time to get to the clinic. I would thank them and tell them that I had fallen asleep. Then I would go [to the clinic]

Collins: Powerful, Powerful. Who did you leave your children with when you went to Epworth?

002: Children… my children were by themselves.

Collins: They were alone?

002: They stayed behind. The youngest one was scared of me because my skin colour changed, my complexion changed… It turned black. Real black. So she was… even adults, I wonder if that is how the drug works, I am not sure because… I changed my complexion and he rejected me.

Collins: Uhh.

O02: Yes, he was afraid of me.

Collins: How old was he?

002: He was… right now he is 7 years old so he was around 4 years, 5 years when he was afraid of me? So he left me and went back to the others, so the children started to… to take care of each other. It’s fortunate that the girl knew that the baby had to bathe.

Collins: Uhmm.

Collins: The older one?

002: She was not the oldest of them all but the fact that she is a girl… she is more responsible than the boys. She knew that the baby has to bathe and how to take care of the baby.

Collins: So can we say your family was now a child headed?

002: Yes yaa for that time they even stayed as child headed family because at that time they had to… as children who were used to living with their mom and dad and we used to have a certain kind of life and then they realized that their father, a man is bound to fail at some point.

Collins: Uhh.

002: They realized that their father was seeing someone until they get to the point of following up with him to the place where he was lodging. There they fought him but I was not aware of their intentions.

Collins: The children?

002: Yes the children. They went and [clears throat] attacked the mistress house. They then took the food that their father had bought into the mistress’ house and they brought it home.

Collins: A-ah!

002: Uhh.

Collins: Alright, so u have mentioned a lot of things. What about on taking your TB medication, did you face any challenges?

002: On the issue of TB medicines the challenges we faced are that there were some tablets that I used to take but I have forgotten the name. Those tablets required one to be determined to take them because even when she gave them to us she said they have a bad smell, they had a characteristic bad smell.

Collins: Uhh.

002: So the trick was to drink lots of water when taking them… Lots of water, and then pause to breathe in. Because by breathing in one would will inhale the scent…

Collins: Yes.

002: so you would pause breathing for a moment…

Collins: It makes you want to vomit?

002: So that you can be stable. But I was lucky that when I was admitted at Wilkins there was someone who assisted me. Uuhmm that man had a good heart because I got to a point where I couldn’t get up and go to the toilet by myself. Plus I think because that was the time when waste was being drained from my stomach so I ended up messing up myself most of the times.

Collins: Uhmm.

Collins: Uhh.

002: He would even carry me from the bed to the bathtub to clean me and change my clothes. Well at Wilkins I had… I was… although I was in pain but I was treated well. I was treated well and I understood that…

Collins: Uhh.

002: Another challenge I faced was that when I was… I think the MDR tablets can trigger up every infection that is in a person. I developed what are called …genital warts. The nurse that I informed about this condition was not able to assist me to get help immediately because since I was on MDR treatment they couldn’t refer me for treatment because they thought I would go and spread MDRTB to others…

Collins: Uhh, uhh.

002: So they did not take any action but I had reported [informed them]. So I was able to go back and I managed to… to walk and get treated but it also took a while. Anyway I got treated but it took a while for it to heal.

Collins: A-ah wonderful. How about eating and feeling hungry?

002: A-ah during the first days of treatment I did not feel hungry.

Collins: You didn’t feel hungry

002: I was not hungry at all. There was no taste in my mouth. I was not feeling hungry. I didn’t feel like I wanted to eat… you do not feel hungry. I was not feeling hungry. I just ate because in my mind I would just think that I want some food so that I can take the tablets but I was not hungry.

Collins: Uhh.

002: I lost the sense of taste, the whole food smelled bad. So over time I started to feel better and I started to feel hungry and food was now needed. These tablets then were many…

Collins: Uhh.

002: They will be many tablets if I am not mistaken I took about 4 types.

Collins: Yes that’s true there are about 4 types.

002: 4 types, some you take 4 at once, some 3, so they were too many.

Collins: So how would you get the food, which type of food did you prefer?

002: I didn't select. All I wanted was something to eat because at that time all the pain was gone and the drug had settled in the body.

Collins: Uhh.

002: Because of the drug you would want to eat a lot, whatever I got even sadza or anything I got I would eat. But I was getting a lot from the neighbours… [Interjection]

Collins: Your neighbour’s really helped you?

002: My neighbours would… some would cook sadza in their home and bring it to me, others would cook potatoes for me. Just because I disclosed my situation to them, so they would monitor [check on] my situation.

Collins: Alright: So I think you touched on one of my questions that I wanted to ask you how you managed to stay on treatment, you have mentioned about the help you got from your neighbors right?

002: Uhh.

Collins: So did you… is there any other way that kept on financing you. When you realized that the money that you had was nolonger adequate, what are some of the things you did to keep you going… [Talking at the same time]

002: I… I sold my kitchen chairs, I sold my kitchen chairs because I wanted to buy food. At that time I really wanted food, so I sold my kitchen chairs. It was a kitchen table with 4 chairs I sold them during that time.

Collins: You sold them for how much?

002: I sold them for USD25.The market price around that time was USD 120 but I didn’t have a choice.

Collins: Apart from that did you do anything else like borrowing money or leasing some of your things like a-ah for money?

002: A-ah I didnt lease anything because I didn’t have anything that is worth leasing out.

Collins: Alright, alright so is there a time when you reduced your meals because of food shortages like reducing meals per day?

002: Yes we reduced because sometimes you would want to eat let’s say porridge… but when i used to do some part-time jobs I knew that if I go out and work when I am done some of the employers would say you may take this packet of rice or bread which was not part of my wages.

Collins: Uhh.

002: But when I was sick there was nothing because sometimes I would just drink tea or I would just have sadza. Instead of having 3 meals I would just have 2 meals a day. Or I would have porridge in the morning, sometimes when I have porridge in the morning it means I will nolonger have tea then I will just have supper in the night.

Collins: Horaiti, I think we are just left with 3 or 4 questions. There is money that is given to assist people on MDRTB treatment how helpful was it?

002: Uhmm I didn’t receive any money, [Background Noises] I didn’t receive any money.

Collins: Did you even have knowledge about it?

002: I only heard that there is money that will be collected in town and you will receive text messages but I didn’t receive anything.

Collins: Did they ask for your ecocash number?

002: Yes I gave them my phone number but I didn’t receive anything.

Collins: Alright, you told us that because you were already on ART and this helped you to accept MDRTB results?

002: Uhh.

Collins: Can you please explain to me what it is like to be on ART and taking DRTB medication at the same time. I am looking at pills here and what is the experience like?

002: Pills. Aa-ah! I was on a lot of pills because I was moved from the first line ART and placed on the second line. In the second line I had a challenge because I was taking Lopinavir and Abacavir. Abacavir is not available in all the places, it’s hard to find. So I had a challenge that Abacavir drug was out of stock. Being out of stock meant I had to buy it myself. So I would buy the monthly supply that costed USD7. So this caused my viral load to go down… to increase tremendously to 37 000 [correcting herself].

Collins: Uhh.

Collins: To increase tremendously.

002: Yes increased, it increased a lot. So I had to take a lot of pills.

Collins: How many times did you experience of out of stocks, how many times can you say you bought the drugs?

002: A-ah even 6 times.

Collins: Alright, apart from the fact that the drugs were out supply, on the issue that the drugs were too many which ones do you think were okay for you and what did they do to you?

002: I realized that I should not tell myself that the pills were too much because my blood tests and the tests that have been been done by the doctors meant they would have realised that it was the best prescription for me.

Collins: Uhh.

002: Because when I first started receiving MDR treatment they told me that with time I was going to finish the course and then I was going to be left with ART only…

Collins: On ART.

002: So I just persevered, the tablets were painful but I endured a lot.

Collins: Which ones were very painful?

002: MDR tablets were too many…

Collins: Those are the ones that are painful?

002: A-ah they are painful. E-eh for ART I would take 2, 2 Abacavir and 2 Lopinavir in the morning then in the evening I would take 2. So they are better. For MDR I would take tablets in the morning, afternoon and in the evening. So I was taking a lot of tablets.

Collins: So you never thought about skipping MDR-TB tablets so that you concentrate on ART?

002: A-ah my focus was on my kids and that was something was at the back of my mind…

Collins: You focused on your children?

002: because of the fact that… plus counselling helped because we were told that if we adhere you will recover because I remember some people who I was admitted with at Wilkins hospital they passed away becaused they refused to take the tablets.

Collins: Uhh.

002: So I took heed to the instructions [during counselling] when they told us that the treatment is painful but we had to endure it… So I looked at these people [paid attention to the nurses] saying to myself that if I fail to take my tablets…. my children are suffering when I am still alive, what more when I am gone. So that gave me the courage to endure the treatment even though it hurt but I was just… my prayer to God was to ask Him to let me live so that I can look after my children… so I persevered.

Collins: Uhh.

002: It was painful but I had some courage in me so I wanted to see how it was going to unfold but I had the will to persevere. Because I could have skipped the injections dropper because it is painful to take injections almost every day. It’s difficult. You end up fining it difficult to sit because of the daily injections.

Collins: How long have you been on ART?

002: I started in 2014, April 2014

Collins: Alright. Right now what do u think your future is going to be like?

002: A_ah right now I can see that I have fully recovered, I am alright.

Collins: Uhh.

002: E-eh if God continues to take care of me I have to work so that my kids can go back to school and their future can be bright. And I have learned that women must never rely on their husbands. One should just be self sufficient. So that you won’t be strained a lot because of situations [when a husband leaves].

Collins: Uhh.

Collins: Okay lets say you have been given a chance to seat on the board that wants to decide the support that is supposed to be given to MDRTB patients, you have experienced this disease, what would you like them to do for the patients?

002: I would like MDTB patients to be provided with food; there is also need to check if they are also eating properly. Because I have seen that when a person is sick there is time when you will be really sick but there is also time when you will start to eat and you will be needing food because I experienced that.

Collins: Uhh.

002: I think the time when a person now wants to eat if they could be assisted with good food and they take their tablets on time as they are told they will recover faster than a person who is taking tablets but does not have food on an empty stomach.

Collins: Uhh.

002: Because you end up feeling dizzy and you can tell that a-ah this dizziness is not pain but its because of hunger. Such people should be provided with food, and also be assisted especially with counseling.

Collins: Uhh.

002: 002: I think probably it was easier for me because of the counseling that I received… I started having counseling long back from the ART program. So I think a person who has never been on ART and is being referred to MDR without [proper] counseling that can take time for him to accept it.

Collins: Uhh.

002: Because I still remember being on MDR that there were some days about 4 to 5 days if I am not mistaken when I got mentally disturbed because of these tablets.

Collins: Uhh.

002: So that requires one to have monitors [counsellors] close by. In addition, people who have MDRTB should disclose so that they can quickly get help. If I had kept MDR-TB from my relatives or neighbors saying… it’s not about just telling your relatives only. You can only find maybe 3 of your neighbors that are nearby… they will call your relatives after they have helped you first.

Collins: Uhmm.

002: It needs you to accept it 1, self-disclosure but not to everyone. As an individual you will realise that if I share with so and so you can get assistance.

Collins: Uhh.

002: Even to family members who would have come to the hospital to visit their relative, the health workers should inform their relative has been diagnosed of MDR should talk to them telling them that this is not the end of their relative’s life but its just a disease.

Collins: Uhh.

002: I think when they disclose that this person has MDR and it is infectious some people will think that a-ah… I think that is what affected my husband. Because when he was told that it is infectious he stopped coming close to me.

Collins: Uhh.

002: But there must be education that e-eh educating them that the chances of contracting it is there but there are measures [infection control mesures] we can take so that the patient is safe and also those who are not sick do not get infected.

Collins: Uhmm powerful. Alright last question, e-eh so if they are to be given food which type of food should they be given and… how will the food be distributed? If its money or food how will it be done?

002: In my opinion e-eh money is needed because some may experience the challenge that I had of children dropping out of school. But if there is an arrangement that they are given food, some people do not know healthy food that they are supposed to eat.

Collins: Uhh.

002: It’s not everyone who knows what is good for their health… it’s like some people do not know that beef can be supplemented [substituted] with beans.

Collins: Uhh.

002: He might think that beef is healthier than beef. So that also requires the Ministry of Health who knows that these drugs being given to… these people. If one can get some peanut butter, cooking oil, dried fish, mealie meal, porridge these are the things that one has to eat on a daily basis and then maybe if they can add some money so that incase the person needs to travel or to cater for other needs and wants that are needed.

Collins: Uhh.

002: But what is most important for me is that people get food. Some people may be given money but since he will be staying with other people they may not care about the needs of the patient and end up spending the money for their needs and wants. They will not value the importance of monitoring this sickness but what we are focusing on is to fight the disease and ensure consistence in taking the tablets and the survival of the patient.

Collins: Uhh.

002: Money is needed, but having a healthy diet is the key. Food is needed and money can be used to buy some of the things that you have not been given.

Collins: Uhh.

002: But food… people should be given food because these tablets from my experience they require food in the human body. People should eat.

Collins: Thank you. A-ah which areas do you think were mostly affected by MDRTB in your life?

002: It affected me and especially my children. My children were affected a lot that all 3 of them are not at the level they were supposed to be. When I got sick my children dropped out of school. E-eh it has a challenge especially if the patient is family leader or the breadwinner.

Collins: Uhh.

002: There will also be need to check on how MDR patients and their families are living. I think even having mobilizers [volunteers???] who can do home visits. Not all people want to be given that help but people can go and do home visits and monitoring. When they get to a home, they can observe the various challenges in different homes.

Collins: Uhh.

002: And help a person in those areas so that they are not burdened. I think if a person is overburdened… I used to meet people at… When I went to the hospital and did not see some of the DR-TB patients for one of two days and then meet after 3 - 4 days they would say, “Aa-ah! How are you doing? You are recovering. Tell us what you are doing to get better faster.” We used to have small group discussions where we would consel each other. We would advise each other to accept our conditions; we would encourage ourselves to pray and we would call each other and so on.

Collins: Uhh.

002: It is also good for people with MDR to form their own group because someone may be overburdened at home but when they meet someone with their own situation they will share…

Collins: They will be sharing.

002: You might think that your situation is worse but when you talk to other people you can realise that you might be suffering but your situation is better than his situation, his situation could be worse. There should be a group for people in the MDR like what is done at the ART program where people can share information or even have parties or having functions where people will be… that will make some people fell like they are not alone, they are many and we have a certain way of life that we should follow rather than shutting ourselves at home while taking the medication.

Collins: Uhh.

002: At home we have faced many challenges to the extent that many of whom I know and I used to collect tablets on their behalf were rejected by their families. They thought that since it’s infectious they thought if they come close to someone with MDRTB they were going to die. The only secret [infection control measure] is to both of us wear masks. That way we will be protected and can live well.

Collins: Thank you so much for the wonderful information you have provided. You know we come to you without knowing things like what you have told us and this information is not available in hospital registers. Thank you so so much. Uhmm the information you have given us like I said earlier on will be transcribed and this audio will be deleted. We will find a pseudo name like Getrude…. We want to see change for the better in the way TB is managed. That way people on TB treatment benefit from our goal. Before we close do you have any questions, we have finished our discussion.

002: My question is that even… the problems we had was not just from the nurses only, we were told that our reviews must be done by a doctor. The doctors were supposed to come to the areas we were in, but we had a challenge that the doctor came only once.

Collins: Throughout your treatment?

002: I saw the doctor once, they would even call him… and the Doctor was supposed to come every Thursday but I don't know if the doctors were also afraid of the MDR. They called the doctor when my ears started ringing and that is the only time when the doctor came and stopped the injections…

Collins: How long were you on injections?

002: I was on injections for 72 days. My ears started ringing around day 44. They told me that when your ears start to ring you should immediately notify the doctor so that he can come.

Collins: Uhh.

002: They called the doctor from day 44 but only came on day 72.

Collins: So far are you hearing properly?

002: Yes from the time I stopped taking tablets… injections they are hearing properly. I can hear properly.

Collins: How about your chest, are you not in pain?

002: At moment I am okay.

Collins: Uhh.

002: I think… plus the injection have some side effects because the ears kept on ringing and you end up not hearing at all. Because the injection is called… I think when the ears start to ring it is an indicator that the drug is now enough in the body because of how the ears will be ringing. So if they take long… my ears started to hear a little bit by bit but by that time they had closed… when they stopped the injection they took a while to hear. They will be ringing like a phone, so much so that it's almost like it’s a bug ringing in your ears… it irritates a lot. So I think if a doctor has been called they should come immediately…

Collins: Immediately

002: Yes to come at once and examine the patients, plus when the doctors hear that… I met a certain woman who said she nolonger wanted to take the tablets and l said uhh uuh do not say you nolonger want tablets contact the doctors first. She said ‘Aa-ah the doctor is not coming and I said wait until the doctor comes because you can stop taking the tablet that is already working in your blood system’. So we just appeal to the doctors to do our reviews on time.

Collins: A-ah thank you so much.

******************************************************************************

END

**Agnella**

**Introductory questions:**

**Collins:** Like I mentioned before in this study we want to understand how you managed to stay on treatment despite the challenges that you may have come across.

**Collins**: Firstly I would want you to give me a brief background about yourself.

**Agnella**: I am a woman aged 38 years. I am staying with my two children, a boy aged 9 and a girl aged 19 years. We have been living in this location for the past 8 years. I am not employed but I used to earn my living as a vendor.

**Collins**: Firstly we want to know when you started your treatment and for how long have you been on treatment?

**Agnella**: I started in 2018 up to 2019, it takes a long period like 2 years.

**Collins:** Did you finish your treatment already?

**Agnella:** I finished my course last year in September

**Collins:** Okay, last year in September?

**Agnella:** Yes

**Collins:** So when you were diagnosed with TB, where you staying here or you were somewhere else?

**Agnella:** I was staying here

**Collins:** Alright, so you didn’t shift from where you are?

**Agnella:** Yes

**Collins:** As a person who once tested MDRTB positive, what do you know or what you were taught about MDRTB?

**Agnella:** We were told that MDRTB is curable, you are supposed to take your pills religiously without skipping at all. It’s a disease that is contagious, it spread very fast...we were told not to go to places with huge gatherings. We were told to put on masks.

**Collins:** What did they say about the way the disease is spread?

**Agnella**: When you cough, you are supposed to cover your mouth then greet someone with those hands.

**Collins:** When you first diagnosed with TB, is there any support that you got so that you continue with your treatment?

**Agnella:** Yes I was counselled from the hospital where I used to collect the drugs, I was well counselled to an extent that I continued with the therapy.

**Collins:** Alright, let’s go back to the time where you started to be unwell...when you had symptoms...that my body is not well, may you tell us what happened up to the time where you were diagnosed with TB?

**Agnella:** What happened was that I started to lose weight, vomiting, not eating well. Firstly I went to the hospital thinking that it was flue or it might be a cough, there was no change and I went to see doctors at Red Cross Clinic in town...I was treated but there was no change and I went back to the clinic. That’s where they discovered that it was TB.

**Collins:** Approximately, how long did it take you to get diagnosed with TB?

**Agnella:** It took me 9 months to get diagnosed with TB.

Collins: Alright, let’s go back to the day when you were informed of the results, what happened?

**Agnella:** I was greatly troubled. To make the matters worse, I was informed that I was going to be injected for 6 months but got counselling and it strengthened me a lot.

**Collins:** Alright, after all that, did you inform anyone that you were diagnosed with TB, did you share the information with others or you kept it a secret?

**Agnella:** I shared it with others, some accepted it and others couldn’t accept

**Collins:** Are those you shared with your relatives or not?

**Agnella:** I shared it with my relatives and friends

**Collins:** May you tell us what you came across with at the hospital, or anywhere else, it might be treatment, side effects, or the way you used to live, or the way the health practitioners treated you, tell us your experience you came across with?

**Agnella:** Whilst being treated MDR, had difficulties in walking...couldn’t walk properly, we were taken well of at the clinic. We had nothing to eat during the time of treatment.

**Collins:** Have you ever had side effects?

**Agnella:** We had several side effects even up to now, I still have them...I have numbness especially on my legs to the extent that I can’t walk for a long distance.

**Collins:** How did it exactly affect you from the way you used to work?

**Agnella:** I was really affected to an extent that I could not work because most of the time I was on therapy, whilst taking medication like that...you can’t work. After taking the pills you feel very weak...you feel like sleeping.

**Collins:** Did you had a time that you couldn’t do anything alone to an extent that you needed help from someone?

**Agnella:** Yes, for the first 6 months I couldn’t do anything...I wanted someone to help me but as time goes on, one gets used to it...but it takes a long time to get used.

**Collins:** What else did you encountered with especially others get to an extent of selling their properties...did you get to such an extent in-order to raise funds for home consumption?

**Agnella:** Yes. I sold my TV set so that I get monies for food, I sold property. At that time it was supposed to fetch $USD50. It was supposed to fetch at least $90.

**Collins:** Can you tell us if you encountered challenges when collecting the drugs

**Agnella:** Uum. At times we encountered problems, after failing to get certain drugs, they would say that we are supposed to go to Nazareth and other poly clinics like Rutsanana in Highfields...they would say, “We ran out of a certain drug.” Then we would rush to either Nazareth or to Rutsanana to look for it. This used to happen several times...they would say, ‘we don’t have such a drug. Go to such a place. It used to happen here and there.

**Collins:** What would they say was causing the shortages?

**Agnella:** They would say we don’t have it and also if we went to pharmacies, you could not find it...you only get it from Nazareth and other poly clinics like Rutsanana.

**Collins:** We also want to know if you had kids who were going to school, were they affected in attending the classes...have you encountered that?

**Agnella:** Yes. My eldest girl failed to sit for her exams that year and she sat for her exams the year that followed. She passed but we could not afford fees for her to continue with education because things were very tough. Even up to now, life is still tough. We had no money for her to write end of year exams.

**Collins:** So, you still doesn’t have money for her to go write again?

**Agnella:** She finally set for her exams but she could not go ahead with her education.

**Collins:** Alright, is she the only one who was affected and others continued with schooling?

**Agnella**: Yes, others are continuing since they are still at primary level. Secondary level was difficult for us since I wasn’t well

**Collins:** Alright, who is helping you to pay the fees?

**Agnella**: No one

**Collins:** Because I heard you saying "we" could not afford.

**Agnella:** Yes because secondary school is more expensive than primary school.

**Collins:** So are you raising them on your own or what?

**Agnella:** Yes I am alone. Am a single parent.

**Collins:** Did you became single after diagnosed with TB or what?

**Agnella**: It came after I was diagnosed with TB. Their father went away. He ran away. Up to now he hasn’t come back.

**Collins:**What really transpired, when you were diagnosed what time during the treatment journey did this happen?

**Agnella:** When I was diagnosed with MDR TB, I think after 8 months whilst on treatment, that’s when my husband ran away from us and I was left alone. Life got tough for us and children. Up to now he hasn’t come ...am still alone.

**Collins:** Is he not communicating?

**Agnella:** He hasn’t communicated

**Collins:** How many children do you have?

**Agnella:** I have 2 children, a boy and a girl.

**Collins:** What made you to continue with treatment otherwise with your situation you might have given up on it?

**Agnella:** What made me to continue with the treatment, firstly my health is very important and my family as well, I thought of my children and it urged me to stay on treatment so that I can be with them.

**Collins:** Have you ever got to a situation where you borrowed from relatives or friends?

**Agnella:** We mostly borrowed from relatives and friends, in-order for us to get food.

**Collins:** We want to know how you got food and money to move from one place to another.

**Agnella:** I would borrow money for bus fare from my friends intending to give them back but couldn’t do so because I didn’t have. They helped me with monies for food, bus fares to the hospital ...I would ask from people.

**Collins:** Are those people your neighbours or relatives?

**Agnella:** Others are relatives and others are my friends

Collins: What type of food you really wanted whilst on treatment, you mentioned that food was a problem, did you feel like eating hips of foods and what was that food?

**Agnella:** TB drugs cause one to consume lots of food. I used to like Sadza every day, I liked fruits, drink and also milk

**Collins:** So how did you handle a situation whereby you wanted your own type of food whilst your children wanted something else?

**Agnella:** It was difficult.

**Collins:** Tell us how it was when you wanted something whilst children wanted theirs?

**Agnella:** That’s when we’d go and borrow from relatives and friends, I ended up borrowing.

**Collins:** Did you get to an extent whereby if things gets hard, where you used to cook thrice per day you ended up reducing to twice?

**Agnella:** Yes. We reduced because there was nothing to eat like in the morning we would wake up and have some porridge, then after sometime I would have some sadza and take some tablets and that was it for the day.

**Collins:** Hmm, there are some money that is given to MDR treatment, how did it help you?

**Agnella:** It helped us a lot but sometimes it could not come so we ended up borrowing and by the time we got it, we were in debts already.

**Collins:** Alright, so which areas did the money cushion you?

**Agnella:** It would cover on purchasing food because an MDRTB patient needs to eat a lot and the pills need one to consume a lot of food. It helped me on food but couldn’t afford to pay rents with it.

**Collins:** For you to get registered in order to get the monies, how was it, was it difficult or it was easy?

**Agnella:** No. It wasn’t difficult for me.

**Collins:** From the people you would be interacting with, through the way you would be discussing with those on MDR therapy, there are sometimes whereby others would be taking TB, BP or even ART tablets also, what would they be saying about it, how difficult is it on them to take TB tablets whilst you are also taking diabetes pills etc., we just need a general answer.

**Agnella:** It’s difficult to take TB pills whilst taking ART as well...it’s difficult.

**Collins:** Which one are they saying it’s hard for them?

**Agnella:** TB tablets causes’ pain, but others are fine, TB pills are painful.

**Collins:** So other pills are not painful?

**Agnella:** TB pills need a lot of food, if you don’t eat, they will eat you up.

**Collins:** Alright, are there no chances whereby one would prefer to take ART pills continuously and skip the TB one?

**Agnella:** Alright, yeah sometimes people would end up leaving TB pills saying ART is better off... but at the end of the day you may fall sick...there is a challenge

**Collins:** So what do you currently say about your future?

**Agnella:** My future looks bright because I managed to finish my medication even though I have some side effects, I experienced lots of side effects on my legs...they are numb such that I can’t walk, for me to walk long distances I end up having pain on my legs but my future looks bright.

**Collins:** Alright, if we are look at your future and the children’s' what do you say?

**Agnella**: It’s difficult with the children because I need to work for them but with my painful legs, I can’t.

**Collins:** What about education, the one you were saying had passed, do you see her finishing her education or that’s the end?

**Agnella:** Uum. I don’t think she will finish because we don’t have money.

**Collins:** Alright, how do you survive before you were diagnosed with TB?

**Agnella:** I used to survive as a vendor, selling items but the moment I got sick I couldn’t manage to do that in order to look after my children...I couldn’t manage to do what I used to so that I look after my children ...paying fees for them...I failed to do so due to this sickness.

**Collins:** Okay, what were you selling?

**Agnella:** I used to do buying and selling...I would sell anything.

**Collins:** So are you saying concerning your life before you were diagnosed with TB and the current life you are living in?

**Agnella:** I was greatly affected because before I was diagnosed with TB I was able to work for myself ...selling stuff and paying fees for my children but now I can’t do anything.

**Collins:** Alright, my last question...if it happens that you are given a chance to speak on behalf of those on TB treatment, what would you say that needs to be done for those on TB treatment? You went through that situation so we want to hear from the horse’s mouth how we can help people on therapy, what would you say?

**Agnella:** People who are on treatment who are going through this TB therapy are supposed to be helped firstly with monies for food, secondly with school fees for our children, thirdly when we go to hospital we are not supposed to pay for the services.

**Collins:** Is that all? Where you treated well at hospital?

**Agnella**: Yes

**Collins:** Alright thank you so much we have finished our interview.

******************************************************************************

**Caleb**

Collins: Like I mentioned before in this study we want to understand how you managed to stay on treatment despite the challenges that you may have come across. Uhh, so all the information we are gathering here shall be reported anonymously. Your name, where you stay or any other information that can identify you will not appear in the final reports so feel free. And I want to remind you again that we will be using this audio recorder is it.

Collins: Firstly, may you please tell me how old you are.

Caleb: I am 33 years old now

Collins: Can you please tell me about your family?

Caleb: I have 2 children, a girl and a boy, the girl is in grade 3 and boy at ECD level.

Collins: Alright. Do you mind telling me when you started the TB treatment and how long have you been on treatment?

Caleb: I started TB treatment in April 2020 up to now in March 2021, am still on treatment which I was told takes 2 years.

Collins: Alright. Did they explain to you what MDRTB is at the hospital, did they say anything?

Caleb: Aaagh! I am not sure from my wife if she was told because of the condition that I was in. I woke up asking my wife what was going on and she told me I had been diagnosed with TB. We asked what TB was since I was not coughing. I know that people suffering from TB have chest pains. That’s when they explained to me that…............

Collins: Where you told my sister from the hospital how TB spreads, is there anything

that you were told concerning MDRTB?

Caleb: Firstly, they asked me if my husband smokes and I told them that he doesn’t smoke but there is that small gadget…which is added some flavours that he occasionally smokes, it’s called a Habra [shisha] and is sold in South Africa. They mix different flavours and they smoke that one with his friends. They then asked if he smokes cigarettes and I said no, they also asked if he drinks beer and I told them that he sometimes drink but not that much. They asked me if I knew where he might have gotten the TB from and I said I wasn’t sure since he works where there is crowd and inhale the dust that is caused by lots of people. They said one can get TB through inhaling air contaminated with TB. Maybe he got it when he breathed in TB from someone who had TB but it stays the body for a long time then manifest later on. I just said Ok.

Collins: We want to know when you felt when you were not feeling well. What did you do?

Caleb: I first realised that my body wasn’t well when I had a terrible headache. Since February I told my wife that I was having a continuous headache and she said the pain would go dismissively. She bought *paracetamol* and pain eazy. I would take these and I would wake up complaining of the same headache the following morning...from there I thought it will eventually stop but it was to no avail. I then started to be get weak and lose some weight. We then realised that there was need for my parents to intervene because it was now a burden to my wife. I used spent the whole day at the shop but now I would sleep at home yet I am someone who used to be up and about looking for part-time jobs and was also into marketing. So they observed that I was now spending the whole week sleeping. They said, “This person is not known for sleeping so it means it’s serious”. Then my parents would come and see me....buying tablets, medicine and the like... It became worse to an extent that I could not recognise whether it was morning or afternoon or to even to know what day of the week it was. I could not even recognise some of the people.

Collins: Hmm. Thanks, after you got to such an extent…it was worse...what did you do to get medical help? We want to know everywhere you went up until the TB was detected. What did you firstly do? You used to buy *paracetamol* and realised that it wasn’t working, what did you do next?

Caleb: When he started to feel really sick, he had held music shows on a weekend and had converted his earnings. I was at the shops and he went to the council bar and when he got there he collapsed. He was ferried to Dr X. The people who took him there did not inform us at once; they informed us in the evening that they had taken him to the hospital and had been admitted. We were told in the evening and he only brought some pills that he had been given. We stayed with my sister who used to stay at the shops. We woke up on the 3rd day and I gave him a toothbrush and told him to brush his teeth...when he went out he fell and bled from the nose and I cried....I then called his father and he said since you went to private doctors what diagnosis they come up with? My nephew told me that bleeding from the nose is a sign of TB.

Collins: Alright tell us the other places that you visited together with the monies that you spent. You went to Dr X, did you spent some monies there? Where else did you go?

Caleb: We went to the place Y....we were now complementing with traditional medicine.

Collins: Alright, maybe if you say when we visited so and so. We went there for so many times and we spent so much on transport and medicine and other procedures that might have taken place there. Even on traditional healing, just state what you paid even if you paid in kind just say so because we want to really understand.

Caleb: On the first days when I was able to walk but was weak, we visited a certain area where there is a certain man who does traditional healing. He told us that he could help us but we could not afford his charges. We told him that we were only able to pay a small amount so that’s where we got some help in the form of traditional medicine. He didn’t charge us but only stated that there was need for large amounts of money. We had brought R800 and we gave him the R800 and we told him that we were going to bring some more when we get it. We have not honoured our promise till now. When my father realised that the situation has worsened, we then went to Bishopstone. At Bishopstone, that’s where we consulted another herbalist and he informed us that there was need for two white goats with which he was to perform the rituals. Initially he asked for a white sheep but we could not find it. He charged us R2500 but my father negotiated with him…my father said he had brought R1000 and he promised to bring the balance of R1000. We bought the two goats for R500 each from the local area. So the money we spend is R1000 for the rituals and R1000 for the two goats then we came back home. Sometimes I would refuse to go and would stay behind. The moment I boarded a car, I would complain of pain. I didn’t want to board a car and be moved around. I wanted to spend the day sleeping. My wife would go in the company of my father and my grandmother trying to find a solution. They would try here and there…whenever they noticed any slight change, they would stick with the healer...when there is no change, they would move on. We were also advised by the traditional healers that we are supposed to go and consult hospitals and other traditional healers who help......they mostly advised us to go to hospitals.That’s when we started going to Newly [a private clinic].

Interviewer: Alright. Let’s start to discuss about Newly, how many times did you go there and how much did you use for the transport?

Caleb: We would use our family car to get to Newly. We would refuel the car with 5L of petrol for a return trip. It is a small car and each trip was around R120. As for the drips…I can’t remember. Can only remember there was a day when I was infused with 2 drips. That is when I was still able see. I only heard that I was given some more at Newly but I don’t know the number. She knows...

Interviewer: So how many drips were infused my sister?

Caleb: 16

Interviewer: How many times did you go there?

05: We went there 8 times

Interviewer: Alrighty. We have finished what happened at Newly, where else did you go?

Caleb: Bridge [Beitbridge]. Yeah as for Bridge I dont know anything but I heard that we went there.

Interviewer: Did you go to a public or private hospital when you got to Beitbridge?

Caleb: Private. We started when he was diagnosed of TB.

Interviewer: Alright. Let’s start discussing about private hospital. At private hospital, we want to hear how many times you went there, who did you consult and also how much money did you pay and what else were you told to buy there?

Caleb: On the 1^st^ day we paid R200 for the card [consultation] and they prescribed some tests. We did blood sugar tests and other tests which I can no longer remember because there were many of us so we would give each other time to see the patient and I was the last one to go in and to be consulted in everything. There was an aunt (his sister) who was talkative. I was the last one consulted on all issues to do with hospitalisation. I was now relegated to menial and hard tasks...when it comes for him to be lifted up, they would ask me to do it. In instances where money was required, they are the ones to make a decision. They would only invite to ask me to chip in with some money for some of the medical procedures.

Interviewer: The money was from you or from them?

Caleb: It was coming from me

Interviewer: Alright. So how many times did you go to private hospital?

Caleb: 2 times

Interviewer: Alright. You paid in each and every visit?

Caleb: Yes

Interviewer: Where did you go after the private hospital?

Caleb: We went to a public hospital and they advised that there was need for an X-ray examination. He had a Chest X-ray and was informed that there was dust in his chest so he was to be given TB pills because he had TB. He was given huge TB pills and was taking 6 of them per day....he only took them for 1 week and they asked to have his sputum tested...and they said he was on a wrong treatment; the treatment for the TB he had was supposed to last 2 years. The doctors said during the 2 years he would get daily injections and there were at least 180 injections. However, the number could be less because they would be carrying out some tests on his ears. So they started to inject him and after a month, they asked for the sputum. So on the second month, they said they are stopping injecting him but they gave him some pills.

Interviewer: Was there any problem with his ears? Did they just say we are giving him some pills?

Caleb: They tested his ears twice. On the first day when he got tested he could not understand what was said, they said they were putting a device in his ear since he had hearing problems already...they said when the device is squeezed it would make some sounds in his ears so he was to respond that he heard the sound but he didn’t give them the signs and they in turn wrote that he had hearing problems. When we came back home, I explained to grandfather what had happened but he said I can talk with him and he hears properly. So if they say he does not hear properly he might end up having hearing problems because they may try to treat him of hearing problem which is not existent. Then we called our friend and he said if that’s what had happened that Prince did not understand the instructions he should be brought back. We went back with him. They tested him and they discovered that he could hear.....we explained to him that he was supposed to respond when he hears the sound from the device that was going to be inserted in his ears...if he keeps quiet after hearing the sound, he was to be given treatment for something he wasn’t suffering from. They said they are to reduce the number of tablets...they reduced them and he continued with the treatment. Initially, we were given 2 week supplies of pills, then the supply was increased to a month. For 3 months we were getting 2 week supplies, and we changed to 1 month supplies.

Interviewer: When you went to hospital, did you go together or he would go alone and how much is it to go there?

Caleb: We go together even up to now, am still accompanying him.

Interviewer: How much do you pay each?

Caleb: On the first days we could not board buses but would travel in our family car.

Interviewer: How much fuel were you using?

Caleb: My father would fuel the car and we would go. He would fill the tank in advance since he had 2 cars. He would park the other one at our place and would leave the keys with my wife. He could not stay with me since he would be running up and down in order to look for some money. He was into selling sweet potatoes, avocadoes and many more...and would inform my wife that there car is on full tank and would ask her to take care for me. I would call his friend who works at council, he had already reported at his work place that he and the other woman would help us. He was the one who would drive the car. Whenever we went to the hospital, he would call to inform us he would be coming to accompany us to the hospital. He would drop us home, leave the car and board the lifts back to his work. Wherever my father came, he would refuel the car using the monies he would have gotten from the sales.

Interviewer: Tell us about the monies you have spent. What sort of help or what did you do to get the monies for you to continue going to the hospital? You talked about the car and fuelling of it, what else happened, it might be what you sold, where did you get your monies from?

Caleb: My wife had joined a rotating savings and credit group in which each member would contribute R1000. So there is a time when she was given the money and I was seriously ill. She once narrated the story to me. I can’t remember but it is either R18000 or R11000 that she spent. She explained about my illness to the rotating savings and credit group and fortunately they all know me and they agreed that she be excused in making contributions since she was faced with a problem. They said she had to solve the problem at her home and she would pay when she gets back on her feet. So she was given that lump sum of R11000, up to now she hasn’t paid it back.

Interviewer: So, what else did you sell either furniture or anything?

Caleb: What I managed to do is I once worked as a cook....I went to a certain man who works at an abattoir to look for place to set up a kitchen [canteen]. The canteen cushions me a lot. When he requests for meat, I can manage to bring it for him. When there are bills to be paid at the hospital, I would manage to do that....currently I have a canteen at the workshop.

Interviewer: Kitchen?

Caleb: Yes. I cook food. That’s my other source of income that really helped me.

Interviewer: What about any furniture that you sold?

Caleb: We didn’t sell any furniture. I sold 2 speakers. The old man [my father] sold a car. He sold his [Toyota] Wish. That time money was hard to come by. It took a long time before my wife got a kitchen [canteen]. During lockdown people were not allowed to move especially the first lockdown....that’s when our father sold his car. Life was tough. Sometimes my father would hire a car when he was away...we would pay R300 for car hire and we would fuel the car and go [to the hospital].

Interviewer: So how much did the car fetch? Was it a new car?

Caleb: Aagh! It was less than a year; it was roughly 6 to 7 months old, we don’t know how much he sold it for.

Interviewer: How much did you sell the speakers for and how much were they supposed to fetch?

Caleb: As for me, whenever I went and played music either at bars or parties, I would charge R1000 or even R800 depending on our negotiations skills. l negotiate. When you charge some R1000 they pay the money instantly, they don’t negotiate. People are different. Others can negotiate down to R700, you end up playing for them because you need money, the little you get can help you in other way. So as for the speakers I knew that as long as I am hired I would continue to make money but I sold them because of the situation that was there. I made a loss because I know if I have the speakers they guarantee a continuous inflow of cash but once you sell them it means no more money will come. You can get it once when you sell them, then you exhaust it. It’s different from hiring them whereby you get the money and the speakers back and would hire them again the following day. I sold them for 700 each and got R1400 for both speakers. I bought them for R3500 each meaning R7000 for the two. I was looking forward to sell them for R2000 each and would get R4000 for the 2 speakers. So, I sold them for R700 each because things were tough for us.

Interviewer: Alright. We are about to finish now, if you are to tell us what you were doing before you were diagnosed with TB, how were you getting your monies? What exactly were you doing that was giving you some money?

Caleb: Before I was diagnosed with TB I worked as a DJ. I had a PA system and I used to play at parties, weddings getting some monies for my savings. When I started this job I didn’t have enough resources; I only had 1 speaker and would play, get some monies and give it to my wife for safe keeping and we managed to buy 2,3,4 speakers and I became well known for playing at parties and I was making money. I started to get unwell, then there was lockdown in 2020 and I stayed at home due to sickness. They would always advise me that even if I get well, there was nowhere I would go due to the lockdown...I stayed at home and there was nothing I could do. I used to work as a DJ but ever since I was diagnosed with TB last year when I started my treatment I have never worked. In addition, I was advised that I must not mix and mingle with people in dusty environments before I finish my treatment since I might meet a TB infected person and may get infected.

Interviewer: So we want to know how much money you were getting per month comparing to the current state, is it different?

Caleb: Per month I would manage to get R2000 or R2500 depending on the month. There are some charges that goes up if its holiday...holidays like Easter and valentine..., On Christmas...people would know that majority of the people would be there, those from diaspora would be in the country so we charge a little bit higher like R1500 per night up from R1000 because we know if someone bought goods worth R10, 000 they can be sold out in one night. So ever since I got sick last year...my health is improving...in November when the lockdowns were eased I managed to pocket some few dollars. In December I delegated the job to my boys that I trained to go and play wherever I was hired. They played on the 24th and brought R1000. When people are coming from lockdown they negotiate a lot saying, ‘Can’t you see that we are also coming from the lockdown....lets help one another to rebuild our businesses.....so if we give you R1000 it would help you to rebuild going forward’. You then realise that it’s better than sitting at home. So on the 25th of December they played again for R1000, on the 30th I played for 1000; on the 31st and 1^st^ of January they played for R1200. So all in all I managed to get R4000. I usually work with 2 boys but in December there were 5 of them because of the demand. We would be advised that there is a party in a certain area that needs a DJ and would assign someone to go and cover there. There are moments when 3 parties were held and were in need of DJs so we would just assign the boys to go and perform and cover up for all the parties. It is different when performing at a birthday party....at birthday parties people negotiate a lot saying they would be doing the parties in-order to impress the kids. There is no other business that they do so we would sometimes perform for R450 or R500 and I would pocket R300 while R200 goes to the DJ. When the situation gets tough, I would rather talk to my father and he would give me the car if it’s available so that I can go by car. At bars, we would strictly do business and would get lump sums. Since then, I have never worked. My wife is the one who is doing the kitchen [canteen] business but was affected by the lockdown that occurred this year. She would briefly go to her canteen and come back home. The canteen business was now difficult to operate because of lockdowns and that’s where we were getting some monies to meet hospital expenses. We would pay R80 on our trips to the hospital; we would pay R20 each and R40 for the return trip, making it R80.

Interviewer: Alright, thank you sister for being on our husband's side in the time of need. If we are to look closely, how has TB affected your life, maybe there was something you had planned to do that got affected, that’s what we want to hear.

Caleb: As for me it affected me a lot because I ended up selling my assets that I wasn’t prepared to dispose. That PA system...! I am now thinking of replacing the speakers that I sold. Plus there is a house we were constructing using the money that we had saved after working so hard and had given it to my wife for safe keeping. We should have finished building the house but the money was spent on medical expenses. So the building project was put to a halt.

Interviewer: Alright. Do you hear properly?

Caleb: Yes. The ears have no problem at all. The problem that I faced was of food....I had problems taking food.

Interviewer: Tell us what happened?

Caleb: If I didn’t take pills, I would manage to eat a little bit since I wasn’t feeling well. You don’t consume much when you are not feeling well. I would eat small portions and my father would encourage me to eat a little bit more so that I could take my pills. After a meal, and taking the pills, I would throw up the food immediately.

Interviewer: So what then happened?

Caleb: Not knowing because the first days of my sickness...my wife told me that I was given a wrong treatment and they later on changed it and I improved a little bit...when I ate and took the pills...vomiting...sometimes I won’t last a week. Per week, I would take the pills for 2 days and would not vomit but would vomit soon after taking the pills other 3 days. So I went to the doctor and advised him of the situation and he told us to buy some pills so that I don’t throw up. But the problem did not go. Sometimes it was better and the other times it would be worse. We went back and told him that there was no change. When we came back, my wife tried something; she sought advice from people and was told that I should be given some lemons. So whenever I consumed food, they would cut a lemon and I would put a small piece into my nostrils and leave it there. When I put the lemon in the nostrils, I would feel better but the moment I remove it, I would be nauseous so I would put it back...when they delay to bring the lemon, they would find out that I had thrown out already. Since I wasn’t able to walk or stand, I would just vomit where I was seated.

Interviewer: So you couldn’t walk and for how long were you bed ridden?

Caleb: I spent at least 2 to 3 months failing to walk but I could speak. I could not walk, when I want to use the toilet, my wife would carry me on her back. She would carry me on her back...for her to support me so that I could walk...my feet were sore. I couldn’t walk. She would carry me and leave me on the toilet seat. She would stand there waiting for me to finish before lifting me back to the bedroom. At the toilet, she would put me on the seat because I couldn’t do that myself. Even to take off my pants, I couldn’t do that. She is the one who did all that; she would take it off and then make me sit. She would take me back....Even bathing myself I….I couldn’t do that anymore. Even feeding myself… I could not do it....She is the one who fed me as I couldn’t hold even a cup or spoon or a morsel of sadza I could not do it. She would feed me after noticing that I could not do that on my own. As for bathing, she would lift me up and place me in a dish and bathe me. My strength was gone. I didn’t have any strength at all.

Interviewer: So is there time where you were admitted in the hospital?

Caleb: Only once. He was admitted around the days when people were dying of malaria quite a lot. So he ended up having hallucination so the nurses said this man is truly having hallucinations and he said he is seeing his grandfather who came to fetch those who had just died so we need to bind his hands on the bed. I then called his father and he said he was going to ask the nurses to discharge him if that is what was happening. His father said it was better if he lost his money on fuel for daily trips to Beitbridge than to have his son’s hands bound on the bed and no one is allowed to visit him. So it was better for him to visit the hospital from home every day.

Interviewer: So how many times did you visit the hospital going by car? For how long did you visit the hospital?

Caleb: We went to the hospital for 3 consecutive weeks.

Interviewer: Alright. My second last question, the incentives that are given to those on treatment, did you manage to get them....are there any challenges you faced in getting them?

Caleb: Yes we got the money but what happened is that....when I started taking the TB treatment in April that’s when I got registered. They collected my phone number, I used a sim card that was not registered for ecocash services; it belonged to someone else. It was registered by someone so they advised me that I was supposed to have my own sim card that registered under my name. I changed the sim card, and registered my new ecocash number. So from April until December they just said since you now have an account number in which they will deposit the money since I was attending clinic for TB treatment... So I patiently waited for the monies from April last year to December. In January, I was asked I was getting the monies, “You continue coming here but have never heard you acknowledging that you are getting the money. Are you receiving the monies? I told him that I never received any money. We were asked to go and see someone whose name we were given. We went to her office but couldn’t find anyone. We called and she asked us to come to Beitbridge and I told her that I no longer had bus fare to get to Beitbridge; I was there today and couldn’t find you....now you are asking me to come again to your office. I told her that XX hasn’t received his monies from the time he got registered. She asked us to wait and see how she could resolve the issue of his monies. So we waited for a week and some money was credited to this phone.....he immediately concluded that it was money from the hospital. I told him to go and enquire at the hospital. We called the office and they informed us to go and get a nostro account so that we could withdraw the money....we called my father we went.

Interviewer: How much money did you get?

Caleb: We went there, showed them my phone and informed them that we had come to collect the money....they asked us how much it was. I told them that I wasn’t aware, when I was about to show them the phone, he asked me to read ...he then took my phone and read...he said that there was a mix up, the account number wasn’t mine...the details on the ID was different from the one on the account. He asked for my phone number and I gave him. He verified with the details on the computer ....he then said I had my own account details...I told him the person who had done the account for me and he asked me to go to a certain wing where they took my number and created an account for me then he asked me to go to the other wing to request how much my money I was supposed to get. They told me that it was USD 200 but I could only manage to withdraw USD185. The remainder were bank charges....that’s what they told me.

Interviewer: So it’s for 8 months

Caleb: Yes. They gave me USD185 cash and an ATM card. I filled in the form....I then asked them if I was going to get the money the same way I got the money. When I got there I was given cash, so what was the purpose of the card? They just said, since I had the card, I would swipe in shops, if I need the money I would get it from the card, so what I didn’t understand was that I was given cash, so when the next batch comes...is it going to be in the card, how am I going to withdraw it?

Interviewer: You just go to the bank and withdraw the cash.

Caleb: Yeah, that’s what I wanted to know because at first they said they were going to give me bond notes [local currency] after converting it. I refused to accept the bond. She [bank teller] said don’t you know that here they indicate that the money is in bonds. I went back to the man and he asked me to go and collect my USD. The man informed me that my account was credited with USDs. He then accompanied me and asked them to give me the money in USDs and they just said isn’t it what we are doing? If it wasn’t for that man who stood by me I was going to be get RTGS [local currency] because they just said...this card that you have is for bond notes. Can you see it? …and I said yes. Then they said, so that’s the money you have. We are going to transfer it to your card then you can go and swipe if you need to buy. That is when the man said, give this person his money. It’s in USD. I was given the money and I left. That’s how I got it.

Interviewer: If you are asked how you want those on TB treatment be helped, what would you say, what is it that you want done, anything that you can think of.

Caleb: As for us…with the expenses that we went through, we were lucky that our families tried their best to help us through. If it wasn’t for them we would be saying something else....people can die because of lack. Sometimes even if money is available and so forth...a person can die also but most of the time those people who don’t have money can borrow from others but if they keep on borrowing, that could end up distressing. The wife would tell you that I have gone to borrow from so and so and that person would in turn see it fit to stay with them since you are always asking from them. Like what I did, I ended up saying to my wife, I am tired of life; whatever that needs to happen, let it happen. We are kindly asking if its money, let it be increased and let it come on time. If we are supposed get it every month, let people get the monies monthly unlike what I went through that I get the money after a year, I think it took me 10 months to get the money. I got registered in April and got it in February...so you see...from April going to Bridge everyday...April 2020 to February 2021, I got the money in February. It was USD200 but I got USD185. Considering everything that we experienced, the money is too little even without factoring in the monies that we spent at traditional healers....traditional healers charge exorbitant fees as compared to hospitals. If you go to hospitals, you only pay for transport since treatment is free, but at traditional healers, you can’t leave the place without paying something. That is how they operate. So we are asking if they could increase the incentives. Even if it’s as little as USD20, let it come every month, not that the USD20 comes after 10 months and we are told it’s for 9 months, what would I be consuming? If there is nothing, it’s better to let us know than to make us happy hoping that we would receive the money that was promised. When you are sick, you would be saying soon I will get some money. If you in turn realise that there is nothing after you are promised that the money is coming...even if it is $10 or $1 after 5 days, let the $1 come after 5 days not to be told that your money has finally come after 9 months.

Interviewer: Alright, we were about to finish but there is something that you and her spoken about...you said I was about to give up...she said I ended up asking for help from my relatives. Please clarify on that.

Caleb: Yes that’s true. As for me, there is a time when I said to my wife, “Uumn, I have moved around a lot trying to get healed, you are now over burdening me by lifting me up to get into the car, taking me to different places. Today I am at Newly, I have been infused a drip, I have cried a lot. I am on drip today, I cried a lot, it’s better for me that I go. May God give me rest because I am in pain. As for my wife, she was always in pain telling my father what I was always telling her. My father would come to ask me what the problem was. My father is the person I wanted to see every day because my wife knew that each time my father was away there was not rest. So I got to a time I gave up because of the situation that was there. There was a time when I would get to the hospital today, only to be told to come back the next day. After going back on the proposed date, they would ask me to come again the following day. So it was changing each and every time...we started going to private hospitals and we were not told too many things. They said they only wanted to do some tests but my wife said that there is a certain doctor at private hospital who told her that we were wasting their time on me since I was not going to make it. That’s what she said….that I wasn’t going to make it.....imagine someone telling you that?

Interviewer: What about where you mentioned that you ended up looking for help from your relatives?

Caleb: Uum. We realised that we had burdened people so my wife ended up phoning my mother in-law. My mother-in-law would ask my father-in-law in Harare to send us some money for groceries and it really helped us.

Interviewer: Thank you two for the important information you have given us....that you ask for your monies to come timeously and to be increased.

------------------------------------------------END----------------------------------------------------------

**Introductory questions:**

Collins: Like I mentioned before in this study we want to understand how you managed to stay on treatment despite the challenges that you may have come across.

Collins: Firstly I would want you to give me a brief background about yourself.

Felistus: I am a single woman aged 22 years. I have been living in this location for the past 2 years. I am not employed.

Collins: Alright, thank you so much for giving us this opportunity to interview you. As we have already mentioned we are doing this interview so that we can understand better the challenges faced by people on DRTB treatment is it.

Felistus: Yes.

Collins: We need to better understand what can help people facing such challenges to stay on treatment, and how you were helped so that you can stay on treatment. We will capture all the information using an audio tape and no video recording is going to be done. Again no information will be traceable to you but it will come out on final results. You can choose a pseudoname that will be used in our reports. So I would like to remind you again that we are using this audio recorder… Sometimes you shall see me writing some few notes… Do you have any questions before we start?

Felistus: I don’t have.

Collins: Alright, alright. E-eh maybe as we begin we would like to know how long you have been on drug resistant TB treatment?

Felistus: For 20 months.

Collins: Its now 20 months? *(Baby crying from the background)*

Felistus: Yes.

Collins: Right. It’s now 20 months, have you ever been diagnosed of TB before or this is your first time to be diagnosed of TB?

Felistus: I was diagnosed before in 2018.

Collins: Ooh you have had it in 2018?

Felistus: Yes.

Collins: Then you were treated?

Felistus: Yes.

Collins: And you finished the treatment course?

Felistus: Six months. Yes.

Collins: OK. Alright. How long have you been staying in this area?

Felistus: Let’s say 20 months because I started staying here in April last year when I was diagnosed with TB. So we can just say 20 months.

Collins: OK. When you were diagnosed with DR-TB?

Felistus: Yes.

Collins: So what made come and stay here?

Felistus: What happened is I faced some challenges with my aunt. I was living with my aunt. When I was diagnosed with MDR they said they were afraid of the type of TB that had infected me, so they didn't want me to stay with them and they said I had to leave because I would transmit TB to others. They said, ‘We are scared of the type of TB that you have’. So I was offered accommodation here. This is a pre-school. I spoke to her about the challenge I had faced at my aunt’s and she said, “It’s not a problem, come and stay. Get treated from my place.” That’s how I came to stay in this place.

Collins: Where are your parents?

Felistus: They are in Masvingo.

Collins: Alright. It’s okay. Alright so did they explain to you about DRTB when you started your treatment at the clinic?

Felistus: Yes they told me… (Collins Interjects)

Collins: How is it spread? How can one be infected with DRTB, what did they tell you?

Felistus: They said that I had been infected with the kind of TB that is called Multi drug resistant. You will get injections for six months then you continue with pills. Of which the nurses did not explain much because the nurses were also scared since they have heard that its MDRTB, they will be isolating us.

Felistus: Uhh.

Collins: How can be transmitted?

Felistus: They didn't. I got help from… environmental health personnel who even told me that I could have contracted the TB from someone or it could have been in my body since I have been infected with TB before… so since it resisted the first line of drugs that is why it has come back as MDR.

Collins: Okay.

Felistus: Uhh.

Collins: Alright. Now we want to turn our attention to what happened back in April. When you started having symptoms, when you started coughing, sometimes night sweats, sometimes weight loss …from the time you started having these symptoms, tell us what happened until you were diagnosed with TB?

Felistus: I used to experience side pains, side pains and chest pains and night sweats. I experienced this for less than a month. When I started having these symptoms and I said maybe the TB that previously infected me was not fully treated let me go to the clinic and get tested. Then I went for testing and when I got tested the results came out MDR-TB positive.

Collins: So it happened in less than a month?

Felistus: Yes.

Collins: Alright, so can you say when you were diagnosed with TB you were able to walk on your own?

Felistus: Yes I could walk on my own.

Collins: It’s okay. So did you pay anything?

Felistus: That time when I started treatment?

Collins: Yes.

Felistus: I did not pay anything in April because I just went to the clinic and they said you have to start taking pills and also coming for injections. So I can say, in April I never spent any money.

Collins: Let’s move on to the day e-eh when you were told that your results are out…

Felistus: Uhh.

Collins: Let's now go back to the scene where you are told the result. You are talking to the nurse or the doctor who is giving you the results. Let's go back the same day. What came into your head?

Felistus: In my mind…I was so worried but I knew I was going to get better because…..I cried that day and I said, “I-ih, am I strong to endure all the injections?” Because my sister got sick from MDR.

Collins: When was this?

Felistus: It was… 2014.

Collins: Before your first episode of TB?

Felistus: Yes. She finished her medication in 2015. So she once got sick with… so I know the treatment she received then. So it really worried me. So I said well… my sister's experience is what I will also experience. One of the things that really hurts me is that when I start to get my injections, my ears… They say the injections have side effects like causing deafness, so I am going to be deaf. So how am I going to do my school work so am I going to do anything in life and that’s what really worried me then.

Collins: What am I going to do about school, can you explain further?

Felistus: School or life because here when I first got sick I had not started going to college and up until now I haven’t proceeded [to tertiary education]. So I said now that I am sick like this am I going to do something in life…

Collins: Ok. You had completed form six?

Felistus: Yes.

Collins: Aright, alright. Let’s us proceed. Did you ever think you were going to die?

Felistus: No I did not… I never had such thoughts because I knew that people can get treatment and they recover because my sister e-eh when she got sick I was not there but she went to the hospital in a wheelchair because she couldn’t walk or do anything but she survived. She was treated and now she has completely recovered like… So that is what really gave me hope.

Collins: Alright when they told you the results and you now have your results, did you tell other people about your results?

Felistus: Yes.

Collins: Who did you tell within your family or other people, who did you tell?

Felistus: The first person I told is the Pastor from our church then this lady who gave me a place to stay and my boyfriend whom I told him I was diagnosed with TB. Then I called my parents and my aunt I told them while I was still at the clinic.

Collins: Alright. Your aunt?

Felistus: Yes…and some of my friends…

Collins: What did your aunt say?

Felistus: Haa my aunt just said, “That’s so sad you have also been diagnosed with the type of TB that your sister once had”. That was on the first day but she started behaving in a strange way on the second day. That is what made me come and stay here.

Collins: How long did it take before you left your aunt’s house from the day you were diagnosed with TB?

Felistus: It took me five days.

Collins: Five days?

Felistus: Yes because when I was at her place she kept on saying I was supposed to go to our rural home for treatment… to get treatment in the rural areas of which the first time I was diagnosed with TB and had been on treatment for two months she asked me to go to our rural home and to come back after finishing the treatment. So I would get treatment there and then come back here.

Collins: Uhh.

Felistus: So when I told this lady, she said it was not ideal for me to change places where I get treatment, I was supposed to get my treatment from one place so that it becomes easy to know the problem…what will be happening. So that we better understand after you have been treated from one treatment facility rather than after changing health centers which makes it difficult to understand your progress.

Collins: Well. Let’s take a look at before you were diagnosed with MDR-TB. What was your way of life e-eh how did you survive? What were you doing in life?

Felistus: When I completed my form 6 I lived with my Aunt. I used to ask her to allow me to sell things such as sweets or freezits at a nearby school… but she’d refuse saying it was better to apply to the Poly [technic] of which no one was committed to paying my fees. I used to apply for nursing training but the vacancies were difficult to come by.

Collins: Uhh.

Felistus: They said….they forbade me [from selling sweets and applying for nurse training vacancies]. They even promised that they would enroll me at Poly but they did nothing.

Collins: Alright, so were your results already out in February?

Felistus: Which results?

Collins: “A” level results?

Felistus: Yes I completed my studies in 2014. All along I was doing nothing.

Collins: Hoo you were not doing anything?

Felistus: Yes.

Collins: Up until 2018?

Felistus: Yes.

Collins: Uhh, they kept on postponing?

Felistus: Yes.

Collins: Uhmm. Alright. So how did MDR affect your life?

Felistus: Uhmm [Sighs]. No. I cannot say… It didn’t… it did not really affect me because of the situation in our country. I can say it did not really affect me because it’s just a sickness.

Collins: Is there anything that you were hoping to do in life that you can say if it wasn’t for MDTRB I would be somewhere better?

Felistus: When the MDR results came out an opportunity had arisen for me to work at Glow [Service Station] after I had applied for the job. Now the week that I was supposed to start work is the same week when my results came out. I failed to go to work and I said to myself let me focus on treatment because I would get injections every day. The drug is too painful for one to do work the whole day while standing.

Collins: Uhmm, about school, you had already took a long time without going to college?

Felistus: Yes. Because my brother is the one who pays my school fees of which he had said he did not have some money that time. So I applied for places which would not require me pay fees.

Collins: Uhmm.

Felistus: Yes.

Collins: So did you make a follow up on your job?

Felistus: I didn’t.

Collins: Alright. Other people say their ears were affected, as for you did you face any other challenges because of the treatment?

Felistus: Two months that is when I started to feel the effects of the treatment when I started to hear very loud noises in my ears and feeling like they are about to close. That is when I went to see the doctor. So I did not get the injections for six months. I received 81 injections, I was injected for two months. For the two months I was injected everyday… even weekends every day on the first two months. Then in July I was injected during weekdays only and I did not go for injections during the weekends. The whole of July I would go for injections 1 week and I would skip the weekends. I would get injections from Monday to Friday then rest on Saturday and Sunday. The doctor then said I needed to stop the injections because of ear problems.

Collins: Both or just one?

Felistus: Both.

Collins: Alright, so how is it now?

Felistus: Right now they are alright.

Collins: Alright, how about in terms of challenges you faced in collecting your medication?

Felistus: The tablets are extremely painful, like if you do not get enough food while taking these pills they can affect even your eye sight but it would happen for a few hours and then you will start to see again. They cause fatigue and stomach pains. The pills are extremely painful.

Collins: You are saying they require a lot of food. So did you manage to get enough food?

Felistus: Food, my food came from the church. Those are the people who really helped me. They brought food every month, sometimes they would bring food that would last up to three months then they would bring some more.

Collins: Uhmm?

Felistus: Yes.

Collins: Did your aunt bring any food?

Felistus: My aunt didn’t.

Collins: Alright, so which type of food did you like? Was there a certain type of food or you felt like you needed to fill up your stomach?

Felistus: We would eat mostly the type of foods that was recommended to us at the clinic. We were told to eat sadza and mufushwa (dried vegetables), caterpillars, chicken, fish… porridge with peanut butter or millet porridge… they would recommend a healthy diet that we were supposed to eat like vegetables with peanut butter.

Collins: So the people from church did they bring these foods or they just brought what they could afford?

Felistus: Some would bring… they would buy peanut butter, cooking oil, mealie meal, some would say since you have such a problem let us bring such food for her.

Collins: Alright.

Felistus: Uhh.

Collins: So what can you say uhmm if there was no such and such and no I would never have finished this treatment? What can you point out that a-ah this, this and this, helped me stay on treatment?

Felistus: Alright, I got most of the support from the church and everything that they brought, even my mother who was not around but far away she always supported me. Sometimes she would send the food that l liked such as peanut butter and millet meal.

Collins: Uhh, alright what about some other things apart from food support?

Felistus: Aaah and the money that I received from the Ministry [of Health] helped me a lot.

Collins: Alright we shall talk about that. Did you ever get to a point whereby you could tell some of the things that you had?

Felistus: Alright when I started staying here since it was a school, I was selling Zapsnax to school children

Collins: Okay.

Felistus: They would buy yes, it helped me also. I would then buy things like relish…

Collins: So you did not sell anything. How far is the clinic where you collected your tablets?

Felistus: Uhmm it can be 5km from here.

Collins: You used public transport is it?

Felistus: Yes. Sometimes I would use public transport… (interjection)

Collins: Was there a point where…

Felistus: Sometimes I walked.

Collins: Alright it’s a walking distance. So you did not sell some of your belongings such as shoes or other things so that you could have transport fares?

Felistus: I didn’t.

Collins: Aah. It’s okay.

Felistus: Uhh.

Collins: Alright, e-eh did you ever get to a point when instead of having three meals, you ended up having two meals so that your food can be enough?

Felistus: Yes. I used to do that.

Collins: There are funds for DRTB you once mentioned it. Did they help you?

Felistus: It helped me a lot.

Collins: Can also tell me how many times you received it.

Felistus: Last year I received it maybe twice then this year I received it in June. It was USD75. That is all the money I received ever since I started my treatment.

Collins: Uhmm, alright we shall talk about it in detail there is some information I would like to share with you about why it delays. So when you registered, isn’t it you are supposed to register?

Felistus: Yes.

Collins: Which challenges did you face in order for you to be registered?

Felistus: At the clinic?

Collins: Uhh.

Felistus: As soon as the results came out at the clinic, that is when I was registered. They took my file and registered me as soon as I started treatment. I didn’t face any challenges?

Collins: Alright, maybe this question doesn’t apply to you we would like to understand, there some other people who are on TB treatment is it you said It has a lot of tablets. Then they will be on ARV treatment again with even more tables. Did you face such challenges that you would be taking several drugs even diabetes or high blood pressure?

Felistus: No I was taking only TB medication.

Collins: Alright. So now how do you see your future?

Felistus: A-ah I think it is good because I have recovered.

Collins: You are now fit?

Felistus: Yes I am fit [Laughing together]

Collins: Alright. Congratulations! It was a long journey isn’t it.

Felistus: It is.

Collins: So would you say TB… okay I have already asked that question… if you were asked to improve the situation of people on DRTB treatment what would you want to improve?

Felistus: People on treatment?

Collins: Uhh.

Felistus: They need a lot of support, especially when it comes to food and financial assistance so that when they are going to see the doctor they can board transport. Then there are some drugs that they are told to… some other drugs which are not for TB because the TB drug has a lot of side effects… So you can be told that you are supposed to buy the medication or you are told to go and see the doctor or you can be referred to Parirenyatwa if you have some other side effects of which all this requires money.

Collins: It requires money?

Felistus: Yes. So if you do not have the money then you will end up saying a-ah I am stopping the medication because I have experienced so many side effects.

Collins: How about at the clinics is everything okay or there is something that you can say a-ah this needs to be improved or fixed?

Felistus: Yes, the major issue at the clinics is that they make you wait outside. You will be isolated and when they want to give you an injection they can just take you behind the block and they will inject you of which there will be people passing by and will be watching everything.

Collins: Behind the block [building]?

Felistus: Yes. They do not inject you inside the clinic or in a tent. They administer the injection outside the building?

Collins: But you will be dropping your clothes a bit?

Felistus: Yes.

Collins: Then they inject you just like that?

Felistus: Yes that is… haa at the clinic. That is what they do in our clinics…

Collins: They do that even to women?

Felistus: Yes. We would get our injections outside. I have never had my injections inside the building… all these days I have been getting injections outside. There is nothing. Not even a shade… they just say, “Let’s go behind the block.” Then they inject you there and you then leave.

Collins: [Laughing]. They just say let’s go behind the block. Alright at least you managed to continue and here you are. You have recovered isn’t it?

Felistus: Yes. Because the nurses will be… I think people with MDR are terrifying to them because the nurses do not even care… though there are others who take good care of you but for others it’s a challenge.

Collins: So what do you think should be done?

Felistus: In regard to the clinics?

Collins: Uhh.

Felistus: I think there is need to create or to build a place that is specifically for MDR patients or to pitch a big tent that MDR can use since they will be few. A place that is designed specifically for MDR patients. It’s better.

Collins: Alright, thank you so much e-eh we have reached the end of this discussion, is there anything else that you would want to say, things you might have forgotten to mention before we finish?

Felistus: Plus at the clinics sometimes they would say… they say we are supposed to get tested regularly isn’t it. Then at some clinics they say they do not have our forms [Treatment booklets] so that they can file our… our results when our samples go for testing… so it delays you again from getting your results even though they always come late.

Collins: Alright.

Collins: So did your results come on time?

Felistus: Yes they did, just that they were delayed because of COVID issues.

Collins: Alright. Thank you so much my sister.

Felistus: You are welcome.

END.

******************************************************************************

**Godfrey**

CT: Thank you for your time. Feel free to speak in either Ndebele or Shona or a mixture of Ndebele, Shona even Engilsh right. Let me begin by saying congratulations for completing treatment. We know it was a long treatment.

Godfrey: Ok. I’m thankful too.

CT: So as we begin may you please tell me about yourself (family, age, and employment).

Godfrey: I am a man aged 44. Currently I am not formally employed. I survive by doing part-time jobs. At home, it’s just that people that I used to stay with at that time are enrolled in tertiary institutions. But at the moment I stay with my father, mother and two grandchildren who are in primary school.

CT: Alright. So when did you begin and complete your treatment?

Godfrey: Eeeh this treatment I…I can’t remember which year it was.

CT: Uumm.

Godfrey: 20…it should be 2014 Im sure. I started feeling sick in 2014. I had a fever. My feet were painful. I could feel like I’m…I had flue like symptoms then realised the flue never got cured. I started coughing then I told myself, no this kind of cough is not usual. There is a problem but I didn’t waste time. No. I went to the hospital. No one had advised me to go to the hospital but what I was feeling is what compelled to go to the hospital. When I got there and they…I narrated my story. Then they said, “Alright, we need your sputum, the sputum sample”.

CT. Ummm.

Godfrey: Unfortunately there was no electricity. Since there was no electricity they said, “Alright, you can come back the following day. It seems electricity was restored when I had just left. Aaah! I then got a call advising me to come back early the next morning or return at that moment if was possible. Ahh return this time and yet the distance is so long. I went back the next morning. Right, they said I had been diagnosed with TB. Haa TB? Alright, Ok fine. Aah then they said alright eeh I was supposed to…do you see the room that you were in?

CT: Ummm.

Godfrey: Behind that there is an EHT’s office. They said aah…isn’t I was also not familiar with this place…They said you go this direction so so so and get into that office you will find an EHT who will help you with the necessary paperwork.

CT: Umm.

Godfrey: Aah then I…I didn’t get lost fortunately, that was very helpful. I got into some office and that office is where HIV testing was being done. There was a nurse who was my acquaintance. She is working for a bus company now. She said “Oh! You have come! Aah, you are welcome”. I didn’t know… I just assumed it was the EHT’s office. Aah then I saw them…aah you have come for testing and I said to myself, “I have no option since I am already here”. The results indicated I had tested positive.

CT: For TB?

Godfrey: No. I had already been told that I had been diagnosed with TB. I was supposed to go to the EHT to complete the paperwork. Then I got into an office thinking that it was an EHT office yet it was the OI clinic where HIV testing is conducted and I was told I had tested positive. All that didn’t stress me. I just said to myself, “No. It’s part of life”. Some people are stressed by that to the extent of falling sick or having near death experiences but for me it was easy. Then….the first treatment was for six months. The tablets that I used to take were…they were 3. I took them in the morning or just after a meal. There were 3 tablets. I took them for some time… they were giving me two weeks’ supplies and then I would go back for resupplies so that they could monitor if my body was improving or to report if I had a problem. I never had any problem at that time. Ahh! Right. I beat the TB and I got cured clean and fine. Aah then time went by… [*interjection*].

CT: Alright so you did 6 months?

Godfrey: Yes six months.

CT: Ummm

Godfrey: Ummm. Right. I completed the 6 months course.

CT: Ooh you completed?

Godfrey: Yes but I wasn’t admitted…I used to collect my medication and go home. After 2 weeks I would come back. Right, I completed it. Then unfortunately maybe it’s because of ignorance… You will be thinking that sometimes if you complete the treatment as someone who smoked and drank alcohol….. I told myself that the illness is over and I can go back to my habits. It seems I made a mistake of reverting back to my dirty habits without knowing that the same habits are the ones that got me sick in the first place. Right. Aah! I spent just a short period I think a year hadn’t even passed. There was a funeral at some place during the rainy season. Aaah! We were drenched in the rain so much on that day. Then from there I felt my body…. from there my body wasn’t well. I had chills and realised something was wrong.

CT: So what did you do?

Godfrey: No. I went home. On that day I didn’t even drink alcohol. I tried but I couldn’t. I couldn’t even smoke. What is happening here? Could it be that thing again? It’s that thing again! I was certain about that. I decided to wait for some change: if I did’t see any change I would go back to the hospital. I realized there was no change. Then I went back.

CT: How long did it take after completing treatment before coming back?

Godfrey: Aah. It was…. a year had not even passed, no. It seems when I started smoking again after completing treatment that’s when the problem started. I realized that I had made a mistake. Then I went back and they diagnosed me of TB again. Aaah. It’s that thing again! They said, “This time you are being hospitalized, 2 months in hospital. This time around there is an injection and some tablets”. I said, no I can stay at home and come for treatment. They told me I wouldn’t manage because the injection was too strong.

CT: How far is your home from here?

Godfrey: The 16 kilometres that I mentioned earlier.

CT: Alright….[*Interjection*]

Godfrey: So if you are healthy it’s a very short distance. I don’t even consider it. But I walked one, two three days haa no. I said boys…my father has a car, I asked my father to pack my clothes. I told them to prepare my clothes and pack them well. I told them I was going to the hospital because I could nolonger manage. On the last day I spent the whole day walking, got to the hospital, entered the hospital, sat and told myself I could no longer manage. I got to the hospital. Isn’t I said there was no doctor at that time? Yes, there was a sister-in-charge and the nurses. I stayed at the hospital for some time. When I was left with a week to be discharged I felt I was strong but the treatment hadn’t worked well enough.

CT: Alright. So all all this time, from the time you were diagnosed with DS-TB and MDR-TB how much did you spend?

Godfrey: Aaah!....money that was spent…especially at the hospital. Aaah there was no food. There was a shortage but my father together with some teachers and others would bring me food every Tuesday when he was off duty. But I did not have any appetite then.

CT: Alright. Alright when you were diagnosed with MDR-TB. Is that right… [*interjection*]

Godfrey: Right still on that, I wasn’t diagnosed with DR-TB just like that.

CT: Ummm.

Godfrey: They diagnosed me with TB for the second time right. Then…just because there was no doctor at the hospital, they proceeded to start me on treatment. Then a week before I was discharged…I’m sorry to say most of the people who were taking TB treatment at the same time with me passed on. They died. Right the doctor was called Dr Moyo, with whom I was kind of related. He was surprised to see me in hospital. He asked the sister-in-charge what my problem was. He had retired and since he had previously worked at the hospital, they requested him to come back after retirement…Aah and he came back. He asked, “Young man what is the problem”, shaking his head. I was due for discharge in a week’s time. He said he was going to come back after doing some ward rounds. Then he came back with the sister in charge and asked what my problem was. He asked whether they had conducted a sputum test and they said they did. He said, “I am not talking about the one that is done at this hospital. You have to send one sputum sample to Mpilo Hospital”. They said they hadn’t. I don’t remember what else was asked. He asked about blood samples that had to be taken there as well, and what else…., urine samples etc. He said, “This hospital does not have the capacity to conduct some conclusive tests. What you are doing here are mere jokes”… [*laughter*]. He ordered them to take all samples by 4 am the next morning. The nurses did it in haste the next morning. They took all the required samples and sent them to Mpilo Hospital. If they diagnose anything at Mpilo they don’t wait for the result slip to be printed and on that day they called and advised that my treatment had to be changed. That’s how I was diagnosed with MDR-TB.

CT: Ummm

Godfrey: So they spent a few days hesitating to tell me that I was here to stay. This was no longer time for jokes. I had to forget about the previous two months admission it was a joke … [*laughter*]. I was going to be admitted for six months for DR-TB. Haa they were quiet for some time but I could hear some rumours that things were about to take a different course… [*interjection*].

CT: How long were you admitted?

Godfrey: Six months plus the two months that they told me to forget. So all in all it was eight months. Aah right then a nurse came whilst I was still asleep. She said, “Aah, may I please disturb you? I came here to tell you that what we were trying failed. So you are now being initiated into a new six month treatment.” You know, I looked to the side and pretended as if I hadn’t heard what she had said. I then asked her if the six months she was talking about also included the the 2 months that I had already done, and she said I should forget about the previous two months. She said, “So this new treatment is different from the one that you know.” Umm…haa I said to myself I was going to see how it goes.

CT: Alright. When you heard that it’s MDR, what, what came into your mind when you heard that it’s a totally new treatment?

Godfrey: Yaah I thought that it mearnt I was getting into the proper treatment then I gave myself hope that I was going to survive.

CT: Alright.

Godfrey: I realized that I was being told about new treatment it meant that the previous one was out and another source of hope was the availability of a doctor. You see. Then I followed that. The tablets were not taken threes per day like before. Haa. Those ones are like a bulldozer. They hit like a big hammer…[*laughter*]. That treatment! No. No. No. No. That treatment hit me hard and made me very very weak and the injection made me sleep the whole day. No.No.No. Then I took the treatment regardless but the problem was appetite because I became nauseous. So, I was very selective in terms of food such that I used to tell my father and my brothers to bring particular foods because any other food was a waste of time. I used to give pregnant waiting mothers the packed food that they brought. They had also become my cooks because I didn’t like the food that was being provided by the hospital.

CT: What kind of food did you like to eat? Which food did you prefer?

Godfrey: Eeh. I used to….things like sausages. You see. I liked sausages. Things like beans. Beans mixed with mealies, I liked that. I didn’t like meat. All the other kinds of food, no. I didn’t like them.

CT: So what did you do to get the food that you liked when you were now at home?

Godfrey: At home… eeh when I was now at home… isn’t I did that 2 months course then the new course was actually for two years… I spent the first six months in hospital….the remaining 1 year six months I completed it at home but I used to go for drug pick up.

CT: How did you get food?

Godfrey: I used to get food yes. Fortunately my father was employed at a mine he was able to haa at home I used to…I had a little bit of freedom by that time you see. I started gaining my appetite gradually I could now manage to eat food that I previously didn’t like. My siblings were very supportive they used to bring me different types of food. I used to request what I wanted and sometimes for sausages. We have a fridge at home. They used to bring lots of food and put it in the fridge so that I could…Then things started to look good gradually and I ended up…but another problem I encountered was vomiting. It got worse as time went by. Whenever it was time to take my medication then it would be as if it had been provoked. It would happen spontaneously an hour before taking my medication. An hour before taking medication a lot of saliva would form in my mouth and I would know it’s almost time to take my medication. Ummm.

CT: Ummm.

Godfrey: Then when I took the medication mmmm. I would swallow too many tablets, more than…maybe they were more than fifteen. Even the injections the dosage was such that the sickness wouldn’t survive in one’s body because it was too much.

CT: Ummm.

Godfrey: Then after 30 minutes or an hour I would vomit. Then I went to see the doctor. They tried to help me before I left. They advised me to adjust the time I was taking my medication just to disrupt the routine and observe if there was a change. Then I had to…I started; I started taking my medication in the afternoon eeeh… I adjusted from taking medication in the morning. I can’t remember what the problem was with taking my medication in the morning … ooh I realized that if I took the medication without eating anything the side effects would hurt. So I decided to what…..to take the medication after lunch in the afternoon but the injection was still being administered in the morning at 10. I then decided to revert back to the normal timetable of taking medication at 10 since I would request people at home to cook something for me. At the hospital we would have porridge at 10 then have tea immediately, it was just plain tea. So taking all those tablets after eating that little was a challenge hence being at home was far much better.

CT: Alright. Now let’s talk about before you were diagnosed with TB, how did you earn some income?

Godfrey: Haa at that time I…aah menial jobs… [*interjection*]

CT: Menial jobs?

Godfrey: I used to work for a bus company from 1992 to 2000. Haa…then I realized the company was struggling then I… I quit when I realized the company was struggling. We actuallywork as security guards most of the time.

CT: Then you went to do menial jobs?

Godfrey: Yes at some mines… [interjection]

CT: At a mine?

Godfrey: Haa not these big mining companies no. The small scale mines… [*interjection*]

CT: Small scale…so what we want…

Godfrey: We were security guards there…

CT: Ooh. You were a security guard. Alright. What we want to understand is how TB affects income like what you were earning before TB diagnosis and after TB diagnosis.

Godfrey: Alright.

CT: How much were you earning per month from those menial jobs during your TB treatment?

Godfrey: Yah. I have forgotten. What we used to earn per month…we used to earn quite a lot.

CT: How much was it?

Godfrey: Haa at that time we used to earn USD yah and at that time it used to be…At those mines it also depends on how much the mine makes from the production and then the actual profile of the mine. We used to earn around $200 and something, USD.

CT: Did you manage to go to work when you were now on treatment?

Godfrey: No, no I couldn’t manage.

CT: Whats your source of income now?

Godfrey: Haa.As of now haa haa I haven’t…actually we depend on…the same menial jobs. So as of now I …I was thinking of; what’s the name of those companies that are advertised in…those earth moving…

CT: Umm. Oh earth moving.

Godfrey: So I was ready to go there unfortunately the Covid pandemic began. Infact I had raised money to book for the…then the Covid pandemic began and everything got to a stand still. Then from there haa… [*interjection*].

CT: So how do you think TB has…how did TB that you were once diagnosed of affect your livelihood?

Godfrey: Haa. Very much; very much, my life had…it retarded my progress very much. My progress in life was retarded very much. Otherwise I could have been at a different stage in life with what I had planned but it all failed…It all failed because of my illness.

CT: Alright so you were on treatment that was long and painful, you talked about injections right?

Godfrey: Yes.

CT: What would you say motivated you to complete your treatment, something you would single out to say if it weren’t for this I would have defaulted?

Godfrey: Before I respond to your question, I had a friend with whom we were admitted together and was discharged a week before I was also discharged. E-eh. You know… I wouldn’t say he was bragging but he taunted me over being discharged and leaving me behind but unfortunately he didn’t make it when he was at home. Aaah right he was discharged and he went home but his sister told me that he was regretting having left the hospital saying his condition would have been better if he hadn’t left. Unfortunately he died. He regreted having left the hospital and because we later on had a new doctor. Otherwise he would have survived like me.

CT: Ummm

Godfrey: I will then go back to your question. Ummm if someone is sick and they do not want to go to the hospital then it means they do not want to live. The hospital should be your close friend, for instance someone who is scared of; who hates the police, mmm mmm be friends with the police because you might need them some day. Likewise, be friends with the hospital. I personally experienced it eeeh I narrated my ordeal of being admitted at the first diagnosis then I was reinfected and then I realized the mistake I had made. Right I was determined to correct the mistake and totally get rid of the illness because I wanted to live. I realized that the hospital is a very important friend for one to shy away from it. So I had to forget about everything else and concentrate on my desire to live. Then after that my life gradually got back to normal. Then these other people who eehh don’t like going to the hospital…people who get treatment and throw it away have died. So we learn from other people’s mistakes to actually realize that if you default treatment you also die. So I cancelled everything and told myself that I now want to live and God helped me I am now fine because my experience was like fighting a war…[*laughter*]. I acknowledged that it was terrible but I gave myself hope and so many…This place is actually a mission hospital which is affiliated to the Brethren for Christ Church. Pastors who used to come to pray for me day and night stirred up my faith. I saw it was important to turn to Christianity because there was nothing wrong with it.

CT: Ummm.

Godfrey: Ummm. Sickness can change a lot of things in one’s life you see. You can’t continue with your habits when you are admitted. Smoking cigarettes, smoking weed, those who smoke weed and other things if one wants to live… even alcohol you stop all that because you want to live. That’s what motivated me to complete treatment otherwise if I had been disobedient you wouldn’t have been talking to me now … [*interjection*].

CT: Aah. Thank you…

Godfrey: I could have…but because I realized that doing this was necessary in order for me to survive. I discovered that God had given me more years to live. So I thought I shouldn’t waste the chance that I had been given hence I had to do as I was commanded.

CT: Alright.We want to talk about that money. We are about to finish….money that is given to patients on MDR-TB treatment. Did you receive it?

Godfrey: Me?

CT: Did you ever receive money that is given to people who are on treatment?

Godfrey: Yes. I received it.

CT: How many payments did you receive?

Godfrey: Ummm aaah ooohh 4 times if I’m not mistaken.

CT: 4 times? But when you received the first disbursement, how long did the first disbursement take from the time you were initiated on treatment, from the time you started getting injections how long did it take? Did you receive it when you were still getting your injections…?

Godfrey: Fortunately or unfortunately the money came when I was in the mid way through that … [*interjection*]

CT: Midway through?

Godfrey: Ummm. Then there was this issue…they once talked about the issue …then after some time when I was midway through my treatment…That’s when the EHT helped me with the paperwork and then he also helped me with everything that was needed for me to have an ecocash account.

CT: How much did you receive per disbursement?

Godfrey: Those funds were actually not being disbursed every month. I think that money was 25 US dollars per month. Since it was USD25 they realized that the money was too little then they…they would keep it for some time and disburse it as a lump sum. They would wait for 3, 4 months then we would receive it as a lump sum.

CT: Alright you sometimes received it as a lumpsum?

Godfrey: Yes. Yes.

CT: Alright how helpful was the money?

Godfrey: That money was very much helpful because I could now buy some things that I previously couldn’t afford. I used to give my siblings my phone. I would give them my phone and tell them to buy me different things and other groceries which would benefit the whole family. I wouldn’t buy things for myself only because I also had dependents. I would say, “Here is some money buy me this, this and that and then buy things that would benefit everyone not myself only”.

CT: Aah. Alright. You said you were tested for HIV, how is it like taking ARVs and DR-TB treatment at the same time? How was the experience?

Godfrey: Yah.Isn’t if you are on both DR and ART they would delay the other treatment depending on which treatment you would have been initiated on first? So if you are initiated on TB treatment they would delay initiating you on first line ARVs because you’d have been initiated on TB treatment. They have their own way of pausing the other treatment so that you don’t take ART concurrently with TB treatment. Then they would resume the paused treatment at the appropriate time…but I didn’t have any problems.

CT: The tabletes are too many, didn’t that give you problems?

Godfrey: In terms of problems I would particularly mention what I said before. Since there are so many drugs taken over a long period ….. I think the sytem will be saturated with those drugs.

CT: The TB drugs?

Godfrey: The TB drugs? Yes. With the way they are so many, together with the injections, my system was now saturated those drugs. That’s why I ended up having side effects like vomiting. Then with ART…I never had any problems because they had said I might have certain side effects like…some would make you do certain things but personally I never had any problems.

CT: Alright.Last question.What do you think could done to improve the situation of MDR-TB or TB patients on treatment?

Godfrey: I-ih. TB treatment needs a lot of counselling for those who are on treatment. Depending on their heart’s desires, their wishes yes and what they will be feeling at a particular moment and the side effects that they will be having they have to be informed about how the drugs work. Sometimes one might react to the drugs and end up…for instance let’s say you are at home then you realize the treatment has enormous side effects then you end up… one stop taking the medication. Reaction to treatment varies from one individual to the other. For some they might affect the eyesight and other things, right, they should be sensitized of all those things. I could give an example of my father who works at Blanket Mine at the mills where gold is processed. So just because they use a lot of chemicals to process the gold at the mills, he was infected and he finished his treatment last year.

CT: Ummm.

Godfrey: Then he was diagnosed with TB at the hospital and got his treatment but was never admitted. So whenever he complained of side effects I would counsel him and assure him that it’s something that will pass.

CT: You said counseling?

Godfrey: Yes counseling.

CT: Anything else?

Godfrey: Aahh! Anything like….[*interjection*]

CT: That can be done for people on TB treatment.

Godfrey: Yah! Eeeh people on treatment need a lot of care. You have to listen to what they say. Right? Some people need someone to share problems with but if you ignore them thinking they are bothering you…some people do not have empathy for patients. You might ignore a patient …people on whatever treatment need care especially in terms of food. Provide them with good food, ensure that they are in a safe environment, and ensure that they bath and that their blankests are washed time and again. All those things….the dust on their blankets or their clothes might delay the cure of TB because they wouldn’t be staying in a safe environment.

CT: Ummm.

Godfrey: Yes. Those are some of the things we need to be conscious of and people on treatment should adhere to the given guidelines because their life depends on it. They must observe this and be determined to get cured and focus on their plans after treatment. You find that life becomes easy if this is done but there are people who do no treat patients with dignity. If you get sick they stop treating you like a human being, you become a burden which they wish could die. That’s very discouraging to people on treatment. You reduce their chances for survival because they will be lonely and think that people have written them off yet if there is love…[*interjection*]

CT: Have you experienced that?

Godfrey: Yah yah yes.

CT: Ummm.

Godfrey: Yes. But not here at the hospital as all nurses had become my friends even all the support staff had become my friends. With the nurses I could even tell them that I don’t like this particular food. They would ask me what I would like, and I would be free to tell them what I wanted. Some of them would even bring me food after they knock off from work or I could give them money so that they could buy me things that I wanted. Or I could ask them to buy some ingredients and prepare the food and bring it to me. They would bring it….[*interjection*]

CT: Have you ever had problems with your ears?

Godfrey: I have never had a problem with my ears but the only problem which was a side effect is the one which I mentioned; I could hear sounds that can be likened to a running engine. Yes that’s the side effect of the TB drugs. Ummm.

CT: Haa alright thank you so much for the time. Haa thank you so much.

Godfrey: I am grateful too.

CT: Haa we really undertstood what you experienced and what you think can be done.

Godfrey: Yes.

CT: Counselling too. That’s whats others are saying so we will also tell them that no this is what they want. Even issues to do with food those are some of the issues that we will explain.

Godfrey: Alright.

************************************END**********************************

**Loveness**

Collins: Firstly, we would like to know how long you have been on treatment.

004: I started in February, on the 6th of February that’s when I started this treatment.

Collins: So you are on treatment for almost years, is it?

004: Yes, its 2 years this February.

004: Alright, so from the time this TB was detected, were you staying here or you used to stay somewhere houses?

004: I was staying here.

Collins: I would like you to explain from the time you realised that your was not well, maybe you were coughing, maybe you were losing weight or you were experiencing night sweats, when you started to be unwell and decided to look for medical help, we want to know what happened up to the time the TB was detected? What action did you take?

004: I started to feel unwell in 2018. I wasn’t coughing neither was I sweating but I had nyon’o. I struggled to breathe and had chest problems. It was like nyon’o such that each time I consumed anything, I would have symptoms of someone who has acids. I went to the doctor and he prescribed medication for acids. So I visited the doctor several times and he would prescribe different medications. He ended up saying if its acids, it was supposed to respond to the pills, meaning it was not. He requested me to have an X-ray so that he could check what the problem was. I went to have an X-ay at Machipisa. I went with the results to the doctor and he mentioned that the other side of heart seemed to have a problem and it seemed as if the heart was swollen. He said there was an infection. So I was given some other tablets, 10 of them. I no longer remember the name of the tablets. He said, if I finished taking the pills and I did not notice a change he was going to change the treatment. I was given the prescription and I went and purchased the pills. I finished the course but there wasn’t any change. My family then decided that we stop going to private doctors and go to a real clinic. We then decided to go to Karanda. The family came together and contributed some money because life that year was tough. We went to Karanda. My brother accompanied me to Karanda and some tests were carried out, they do several tests in Karanda and they advised me that it was MDR but I wasn’t coughing that much. When they mentioned that they were going to do some TB tests on me I was surprised that they want to test TB yet I didn’t have any known TB symptoms. Since I never stayed with someone who had TB, I just told myself that I didn’t have it. But they said, since you are having chest problems and breathing difficulties let’s carry out some TB tests. They requested me to produce a sputum and I told them that I did not have any sputum even if they wanted me to cough, what was I going to cough? They asked me to breathe in heavily and breadth out, and to try to cough from deep down then spit in the container. I did that. People were laughing at me....there were a number of us, it was easy to some since they had the sputum already. I produced a lot of saliva and we waited for the results which came out instantly and I was informed that I had MDRTB. So, when they gave me the results, they gave me a person who accompanied me so that my papers could be processed quickly. I was taken to see the doctor but I don’t know his area of specialty. I was then informed that I had TB called MDR but they could not treat it in Karanda. When we went to Karanda, we had an uncle who works in the Lab. We put up at his home that night. So when I got tested I was then told that I could not go back to my uncle’s house because I was to be given a bed at the hospital. I was admitted in my own ward and was alone....since I was not supposed to mix with others. They informed me that the following day, I would go back to a clinic which was close to my home so that I could receive my treatment from there. I told them that I know of Nazareth where they deal with TB cases, they gave me a letter and I took to Nazareth. When I came to Nazareth with the letter, I didn’t know that they don’t open during weekends. They told me that they don’t work during weekends. I went back on a Monday with the letter, it took some time for them to accept me. I went to the TB section and they advised me to go to the wards ....they asked me to see the doctor in the wards. When I went to the wards the ward staff that area told me to go back to TB section before I saw the doctor...they said that’s where I am going to get my help.....I kept on going up and down in order to see the doctor....that time my body was weak but I had to be strong till we saw the doctor from the wards who managed to help us but it took me 3 to 4 days to get started on treatment. They said, “You were transferred from Karanda where you were diagnosed with MDR, so what are we supposed to do?” They firstly asked me to re-do the tests and it was done. They wanted the tests to be done at the institution so that they could start me on treatment. The tests were then done but I couldn’t go back [to Nazareth] and collect the results because the doctor from the ward section wrote all the treatments then transferred me to Budiriro Polyclinic. He asked me for my nearest clinic where I could go and I told him that it was Budiriro because that’s the one I knew was providing such services. I didn’t know of any other clinic that was close to my home. When I got to Budiriro Poly, I showed them my papers and they said I should go to the satellite clinic which is in my hood.

Collins: Alright. It was a journey. I want to go back to where you are advised that it was MDR in Karanda. What got into your mind after the results were shared with you?

004: Oh! I was really disturbed in my mind because I didn’t know what MDR was all about because they didn’t explain it to me. It didn’t hurt me that much because I couldn’t understand what it meant. I could not find anyone to explain it to me and also the time to do so. So I had to board the bus and came back expecting to hear the explanation where I was going but the way they did it at Nazareth disturbed me a lot, after being advised in Karanda that I was not supposed to mix with other people. I could no longer sleep at my uncles house but at hospital....I was told that it was MDR ...it disturbed me because I didn’t know what kind of a TB it was, .....what kind of a TB it was. Because when I came back home and reported the results, no one could understand it to an extent that my father went around asking people if they knew anything about this particular TB which people say it comes from South Africa and it resists medication. It disturbed him and the whole family. They were all asking what kind of a TB it was because the one we knew ...when you get treatment you get healed but this one from South Africa was said it resists medication. So it disturbed us a lot. What really touched me is failure to get someone who could counsel me about the TB. Two of the DR-TB patients whom I met at the clinic helped me a lot. I would ask them for information and they would give it to me. Health care workers did not provide any information for me so that I could understand what MDR TB was all about, what it means, and whether is it curable? What I could only see was my end; death was fast approaching. I said to myself, “If this TB resists medication, is there any other medication that is going to treat it? No.” It really stressed me even if you ask my father, I went to the clinic on my own and was strong then I wasn’t that pale then but the depression of not knowing what kind of a disease that I was suffering from stressed me. I deteriorated. What they say, if one goes to the hospital and get medical attention, he would feel much better. For me when I went I was better and came back worse. I could not find information. No one could explain to me what the TB was all about. It took me a long time for me to get a health worker who could just explain to me what the TB was all about. The only information that I got was that it was infectious so I had to put mask-up every-time.

Collins: We want to hear how TB has affected your life. How you used to survive, what other means of survival were you doing when you were strong? What were you doing for a living before you got sick?

004: I used to do buying and selling before I got sick. I was a cross border. I would go to South Africa to sell goods. Sometimes I would stay there for a while until my goods were sold out. I then quit the trade and came back home. I then started hoarding goods from South for resale here in Zimbabwe. I would also go to Zambia to buy goods for resale here in Zimbabwe. That’s what I used to do.

Collins: Alright. Now you are diagnosed with DR-TB. Did it change your way of survival?

004: The selling stopped instantly. I couldn’t do anything. I was sick. It wasn’t well with me. Before I knew I had TB but had nyon’o I used to go to Zambia. I would go today coming back the following day. It would affect me but I would go and come before I knew I had TB but the moment DR-TB was detected in January 2019.....from that time, I was bedridden from the 1st of January to December. I went to the Cross over service on the 31st of December 2018, when I came back, I was bedridden from that Cross over night. I was bedridden from the 1^st^ of January. I kept on feeling unwell without any strength. I was feeling dizzy up until the time I started treatment. The treatment on its own complications.

Collins: Alright. Let’s look at the treatment itself. Tell us your experiences.

004: Firstly, I want to narrate what I experienced at the clinic. The satellite clinic where I used to go, they were always late in ordering my pills so I couldn’t get them. I would go for my medication or the injections. What’s their name? And I would find none of them. So I would get to the clinic but with no tablets, and would be advised to go to Budiriro 1. There is another time I met a challenge that they send me to Budiriro 1. When I got there, there was this nurse who screamed at me and it disturbed me a lot. She said “You people from the satellite clinic why do you come here? You are exhausting all the tablets that are ordered for other patients. Your tablets are ordered. Go back to the satellite and ask them to order medication for you. You see that? I am the one who is supposed to tell staff at the satellite now. You could tell that the nurses have challenges, sometimes they have monetary challenges, am I as a patient supposed to go and tell the nurses to order my medication. Is that possible? It will be difficult. Sometimes I would go [to the satellite] and would be brave enough to tell them that I was being scolded at at Budiriro 1, they are saying that you are supposed to order our medication. They would just say they are supposed to give you, they would answer whatever they wanted. That’s the challenge that I came across. Sometimes we were given expired tablets. On that one I was informed by a certain person who asked me to check the expiry date because one can get expired medication. So when I checked the other day I discovered that some of the tablets I was given were expired and I had to go back. I would show them the pills that they would have given me. If I had not done that, I would have taken expired pills. Sometimes they would have expired 2 to 3 months ago.

The other challenge is lack of knowledge about MDR among nurses. As for me, I would suggest...I don’t know if you are able to train/teach these nurses about TB. Many of them don’t have enough knowledge Sometimes I would get to the clinic and would be served a new nurse. I would be in trouble that day. They told us (TB patients) to come early in the morning so that we could collect our pills. They would open their doors whilst we were at the clinic already, but it was impossible to for us to be served and come back home before 10am. I would usually leave the place between 10 and 12. By then, I would be very hungry and weak, then stay at the clinic from 8 then leave at 12 and sometimes 11, they would send us to the backyard. When you go to the satellite clinic, they would send us to the back and would attend us from there. That’s where the drainage system is. It’s always bursting, that were they told us to sit and there are no benches. I once talked with the other patient asking her if we are really live people like them. They would send us to sit at the drainages for them to treat us there. That is where there is no shade, no tent...we would sit on the grass which is contaminated with raw sewages. Is that healthy? They exposed us greatly, sometimes it would rain whilst we were outside. They would not allow us to get indoors since this sickness is infectious. But we didn’t have anywhere to sit even on windy days even when it was hot outside. Even during injection time, they would inject us while we were outside where we could be seen by people who stays close to use. They are some houses which do not have dura-walls, some have fence on their yards...they would be seeing us getting our injections.

Collins: While you were outside?

004: While we were outside. Sometimes they are people who used to gather in groups, ladies and others...they would be having their meetings. I don’t know what the meetings would be all about, they would be there….they would see us being injected whilst they would be looking at us. They would watch us getting our injections. There is also another challenge that we need counselling. As for me, if someone is diagnosed with MDR they should have someone who can explain it to you even if you have a disease they don’t. Isn’t it that people who are infected with corona are not housed in one place? We should have seen people overcrowded in a ground like Zimbabwe ground there....made to seat in the sun so that they could not spread the infection to others. Are they not managed from shades and rooms? But us, we would be outside there, just like rubbish. We are treated as if we are not humans...even the counselling itself...just to assure us that we will get well. It doesn’t matter even if they lie to us....even a person with cancer that has spread all over the body ...but the doctor assures her of life....it settles one’s mind and not kill you. Your mind can kill you before you are supposed to die.....but you can see that there is nothing, totally nothing, you would then think that, u-ugh there is no life that I am coming across with here. The way I am being treated doesn’t show any hope that I am going to survive.

The other challenge happened the days I got sick, life was tough. I don’t if this treatment causes one to be selective when it comes to food. There is a type of food that you’d loving. You will be loving delicious food. Food like Pizza (laugh ...hahahah). There is a time where you feel you would need certain type of food And that food…that is the one you would need at that time. If they bring sadza and vegetables, you would not want it. You heart would have disliked the food. After eating veggies it would be difficult to take medicines. So it was difficult because my father here supported me a lot because that is when some of his pension came out. All of it was used to cater for my treatment....when I became sick with MDR, ...there are some other diseases that would pop out that needed prescription....they won’t be having pills, bandages and they would be telling you that they don’t have betadine and they would write a prescription. My father would take money from his pension. I exhausted all of his money (laughs) buying medication. He would buy food, whenever he realised that I wanted polon, he would buy the polon, he would buy it but wont consume it himself and others but me only. Thats when he discovered my favourate meal on those particulars days. When he sees that I need spuds, it becomes my favorite on that particular time...whilst others woud be eating junk food ....myself would be taken care of, I would have want I want.

Collins: (Laughs hahahah...) Yeah, your father did a great job for buying yummy foods. What about the sickness itself, you once mentioned that you had wounds, explain what exactly happened....what you exactly came across stating everything.

004: In sickness? Yeah, I was very sick. When I started treatment, isnt it I told you that I wasnt coughing?

Collins: Umm

004: That coughing, was discussing with others at the clinic and they were saying that but you are not coughing and I told them that as for me I dont cough, I was bragging to them saying uurgh I am of grace, I dont cough. I started coughing and I took a long period of time coughing, its approximately 5 months coughing and failing to sleep, I could not put my heard down......my father would hear the cough in his bedroom...coughing the whole night....the coughing became worse during the night and had to wake up and sit down, up to dawn coughing. Some of the time, I could not breadth well, all that, I came across with. So that coughing, I think it caused me to be swollen in here..... I was even afraid to cough, I would try by all means to suppress it......feeling that in my chest.....so that when I started to be swollen in here...it started swollen continuously....it became whitish like I showed you that time. It became whitish, so all the time I was visiting the clinic and every time I went there to collect pills I told them about it. You know what, even if I told them that I was facing this and that, they would just stare at you, write down the medication and give it to me or prescription to go and buy some pills. For them to check how it looks like, no one bothered. Up until the time I got sick, I was very sick...isn’t it that I showed you the photos. Up to an extent of oozing the puss, in my neck area....they would put some clothing. I was swollen to an extent that these became big to a size of reaching the shoulders. When I go to clinic and let them know of the situation, they would write a letter, to go and see the doctor. I would then go to Nazareth to see the doctor. When I get to the doctor and narrate my story, he would write a referral letter and stamp it for me to go to Parirenyatwa, I get the paper and went to Parirenyatwa, got to Pari, went to the reception area and told them that I was told to come here. He read the letter and asked me whom I was referred to see. I told him that the doctor from Nazareth referred me to come here and then he said, uugh but on this one you were not told whom to see, I don’t know the title of the responsible person I was to see, I told him that I also don’t know, am a patient I don’t know how you operate. I was given a referral letter by the doctor and advised me to come here at Parirenyatwa, come at the reception, you are the ones who knows if I am in this situation, you go that side, nothing, was hopeless. I phoned a certain lady we go to church with, lucky enough she was at work (Parirenyatwa), I told her that I was referred to Pari but I don’t know where I am supposed to go, those at the reception doesn’t know as well. She offered to help me, she took me to the other side (outpatient) and she processed all the papers. Then she took me to where I was supposed to go and see the doctor. We went there in the morning up to 4pm....the doctor wasn’t there yet. I was attended to when the doctor came in and he did biopsy and asked me to go home....I said to him.....with everything that you have done....can’t you give me some tablets or anything to drink....am having pain....can’t you write a prescription or anything? He then wrote prescription and asked me to go and buy cocodamol tablets....and I asked what about this place where you have cut doing biopsy because it was very swollen so when they cut the piece of flesh the puss oozed so they (svina), after that they put beta dine and a bandage. I asked him how I was going to take care of the area when I got home because I don’t know, I know nothing.......I might get home and do the wrong thing, I might put traditional herbs doing the wrong thing. He advised me to go to the clinic where I collect my TB pills so that they would give me bandages and beta dine. I came back home, and went to the clinic to collect bandages and beta dine and they said they doesn’t have, they wrote a prescription and went to buy at the pharmacy. It never shrinks, (kusererera), they told me to wait for the biopsy results, I waited and waited....then they phoned me telling me that the results were out but there was need to repeat the biopsy. So the time they said I should repeat biopsy I had a wound on the other side, I had biopsy on the left side and on the right side....I had a tumor that was cut off....it was white....I asked the people at the clinic for advice, I wasn’t sleeping at all. The prescription that I was given for cocodamol, is finished and see what is needed here.... They told me to go back and see the doctor uurgh, they said I should go back to Parirenyatwa and I told them that I was told to wait for the results at Parirenyatwa....What am I going to say to them? Some other people from the community, those in the health sector helped me. I think you know people from the community. They told me that it was better for her to quit going there and those are the days when Parirenyatwa hospital was sid to be shut whilst I was in this situation, there was another lady from our church who works for a doctor.... she told the situation that I was in was bad, lets go to Dr Duri ambonokuona. We prepared and went, I had a system of collapsing, collapsing to an extent of not knowing anything, people would hem tell me that I had collapsed and was quiet. My father panicked the other day and he called people telling them that the situation was not good at home....she fainted and was taken by a certain lady from our church to High glen ....at High glen they charged a lot of money and they also wanted to have some blood tests in order for them to diagonise the problem. We couldnt afford to pay the needed monies. I went back to Highglen coming from Dr Duris surgery....to have some blood tests....uurgh they told us that the blood that was left in my body was little....it was 4 pints only yet a person needs to have 12 pints but in my case it was 4 pints left.

Collins: Hoo 4gramms per decimetre, ndo hemoglobin iyoyo

Collins: Ok, 4grammes per deciliter, that’s hemoglobin.

004: (laughs) we dont know what it is called.

Father: So she would usually faint and she couldnt have proper sleeping position, she would just sleep anyhow .

004: Yes, I would just sleep, when I wake up for porridge in the morning,.....just after that I would sleep......in the afternoon again, I sleep. I was always sleeping and I really felt better by sleeping, in the afternoon and evening...would be sleeping always. So people would woke me up to sit but I felt that I should continue with my sleeping always. So thats when Dr Duri presccribed me some medication. He told me to buy tablets, Yeah the pills were too many. Others were for adding the blood and injections...for me to be injected. So, since the lady stayed here in Budiriro, there are some injections for Vitamin B12, when I started it was a monthly thing then skipped whilst they give me medicine. When I wasnt well, I would then go to that doctor. He is the doctor who helped me a lot and is the same doctor I would ask questions on what I should do. He would tell me, helped me, even as for these wounds...he would encourage and strengthen me that it was going to be well. I started to have that strength plus I had lost weight since I was diagonised with TB.....isnt it that you lose weight.

Collins: Umm.

004: At the clinic, they dont do that, they dont weigh you on the scale for them to see your weight ....that the person we are treating....is he gaining weight or losing, even to check the BP for them to see, they dont....you would only go for the pills only and come back, to examine if the patient is improving , not at all. We would go to clinic to take pills ony and come back and no one would bother to check if the person we are giving treatment, what is changing. I wanted to ask if they dont check BP, or to be weighed on the scale for you to see that after 2 to 3 months, how does the patient we are treating responded, is it improving or its becoming worse. Becuase you can give me treatment and I become worse, and worse and worse. They should see that uum maybe the treatment that we are giving her is not responding well after seeing that the person has gained or not gaining at all. But you are only given treatment to gulp and gulp.

Collins: Ok but you managed to get help, these ones are called lymph nodes.

004: What are they called?

Collins: Lymph nodes. When you consulted Dr X, did you pay?

004: I didnt pay at Dr X, he helped at free of charge. I was surprised what manner of love is that because we know doctors need their monies but maybe its because of the lady we go with to church...he might have told him about the issue, he helped me at free of charge. HE would help me for free but as for medication he would write and I would purchase

Collins: You have covered lots of questions that I had, which drivers can you point at...that if it was not for so and so I shouldnt have finished taking the medication?.

004: Hoo ok, you ean if it wasnt for this and that?.

Collins: Yes, what caused you to keep on taking your treatment?

004: What made me to continue with the treament is the love that I was shown here. My father here and family showed me love and they didnt put me away, instead they drew me closer to them monitoring me whether I have eaten or taken the pills. It got into me to an extent that even if I fainted, even if I fell very sick that day, I would know the time to take my pills. You would know that people doesnt have money but they are putting their effort so that I have a life so I had to see that I have to be on treatment nicely....also I have seen the way my family was worried. I was just praying to God so that I get healed......... so that all those who were standing by me would be happy one day, that really helped me, the love from the family and community that I was in helped me to continue with the treatment. Also what strengthened me to know that I would be well.....people were coming and encouraged me that I will be fine....there are some other people who would give their testmonies saying my so and so came across the same situation and got healed. A certain family friend of ours....the wife to my father's friend...came saaying that she onced was diagonised with MDR but I got sick and was treated in South Africa, its treatable. The fact that you are told from someone who once was diagonised with the same disease that you have.......the assurence that you will be fine....... and the difficulties he went through, ......some would tell you more difficult situation he might have come across with. It makes you strong, I became strong that I will be well one day. Some people saying....my uncle suffered from this MDR and got injected but yours are too many....we were injected a few , someone took the whole year, the other one some months.....people were testfying different testmonies. They were saying, I would be well, I would be strong, I would go back to South Africa or Zambia. It encouraged and strengthened me and I felt that I should keep on taking treatment so that I will be well because at first I lost hope that the treatment was very painful plus what I was coming across with at the clinic that information was not dispatched.....and also the sickness itself, it wasnt changing...I was seeing as if I was getting worse.

Collins: You mentioned that your friend explained to you some of the information, did you get any support either from the other patients you were together with?

004: I met patients **whom we were recieving treament together**, also those with relatives who suffered with TB and those who were diagonised of TB. They were all well, very fit to an extent that if they dont tell, you wont know it. He would explain to you that I suffered with TB and I did this, was carried in the wheel barrow......was like a little baby, I did this and that and now can do this. I strengthen myself.

Collins: Alright, have you ever had problems with your ears?

004: My ears still have problems even up to today. At the moment I hear some echoes. The right ear can’t hear so I use the left ear.

Collins: Alright. Did you receive monies that are given to those on MDR treatment and also did you came across difficulties in accessing it?

004: Aaah this money took some time for it to come so it came here and there, it doesnt come monthly. Sometimes it comes and then stops. Like the last batch was in December. There is a time we heard of problems with ecocash....it was said ecocash should be cheap and thats when it was stopped again and were made to open accounts from ZB bank and we went there at ZB to open accounts.....and I got some of my monies which was for 2 months....I was later phoned by an uncle who works at Glenview telling me that there was money in my account. So since we didnt have travelling documents , we were afraid to move without letters.

Collins: But how helpful is it to you?

004: Yeah, it helps, it cant exhaust everything but it helps. To tell the truth, like myself and the situation that I was in ....if you get it, you would need to buy some sugar or cooking oil and everything since I am someone who would be just sitting with no income so the monies would be little. Like now, I have problem with my legs, these toes shake/shiver on their own, do you see that? When you see it, it will be moving on its own, I dont know whats the problem with them. I also have lumpness from here, and also in my joints here, sometimes they stick, even when I am sleeping, I feel it in my hands. I would have gone to see the doctor or clinic to get services but I dont have the money but you would need medical attention and food. For one to balance it, it cant. It cant meet.

Collins: So what do you think those who are donating/giving those monies should do, what is that you want?

004: Can we tell on what to do?

Collins Yes

004: They should disburse the money on time. Even if it’s little but it should be disbursed on time.

Collins: Makatarisa ikozvino tirikuona mange muri pa treatment, haagh ikozvino zva improver zvakanyanyisa kubva pane zvazvange zviri. Makatarisa ramangwana mukuriona sei ramangwana renyu?

Collins: If you are to look now, you were on treatment, there is a great improvement now from what it was. If you are to look at your future, how do you see it?

004: My future?

Collins: Uumm, muchadzokera here pane zvamaiita, mave fit here , muchi compare nezvaive muri naikozvino

Collins: Uuum, are you going back to what you were doing, are you fit, comparing to what you were like with now?

004: Ramangwana rangu rinoziikamwa zvaro na Mwari. Saka ini ndinenge ndichingotarisira kuti zvichanaka, handifanirwe kutarisa kumashure kana kurwara kana chii. Ndikutorisira kuti ndichatanga zvandaita kana ndasimba. Ipapa simba harisati ranyatsowanda zvaro semunhu akaita gore rose nemwedzi ndakarara saka ndiri kutotarisira kuti simba racho iroro ririkuuya zvaro mbijana mbijana. Ndainge ndisingakwanise kana kusimudza 5litres dzemvura but ikozvino izvi ndiri kutosimudza 20, ndiri kuona kuti zviri kutochinja ende ndiri kutarisira kuti zviri kutochinja nezita raJesu. Ndiri kutotarisira kuti zvinofanirwa kuita, kuti nditoshanda futi.

004: My tommorow is only known by God. So as for me I would be wishing that it will be well, I am not supposed to look back or get illness or what. Am looking forward to do what I have been doing when I get well. I still dont have enough strength since I spent the whole year and some months on the sick bed so am looking forward to gain strength little by little. I couldnt even carry the 5litre bottle of water but now I can carry 20 litres, am seeing a change and am its changing in the name of Jesus. Am looking forward that it will be well, that I will work again.

Collins: Horaiti, mubvunzo wekupedzisira, pane zvimwe zvamatoti bate bate, pamange muchitaura mataura zvakawandisa. Ende zvizhinji zvacho zvinopindirana, zvatinenge tasangana nevamwe vachitaura kuma interview unoona zvichinopfekana kuona kuti ichi ichokwadi ichi. Asi imimi mukanzi mapiwa mukana kuti mutaure kuti chii chotoita mu TB treatment to improve the way yairi kuitiwa kana kuti ku improver wo upenyu hwevanhu vari pa treatment kuti vakwanise ku accesser treatment , zvii zvamungati izvi zvofanira kutarisiwa izvi?

Collins: Alright, my last question, there is something that you have already covered when you were answering some questions. Most of the things intertwin, what we have come across others testifying in other interviews....you can see that it marries and you can tell that its true. But if you are to be given an opportunity to speak or point out on what we are supposed to do in TB treatment to improve the way it is being done or to improve the life span of people that are on treatment for them to be able to access treatment, what is it that you can point that needs to be looked at?

004: If a patient is diagonised with TB, there should be some counselling that can give him hope ....that these pills you are taking...you will be alright. A certain erlderly woman who was diagonised with TB at Satelite and she went to Poly clinic and she was screamed at, I dont know the way they harassed her and she went to another clinic and was harassed again. She ended up saying that she was giving up on taking the pills, I dont know she was helped because she had decided to surrender saying that she was going back home, if she was to die, she would die because of the way she was harrassed

What I see as important, the way they handle the patients of TB, they should give them hope that there is a future ahead. And also they should have a place where they say this place, it might be a small space...they should put a shade to cater for the TB patients and put them at a place designated for them not to throw them away, putting them where there is rubbish and say thats where you are supposed to be.

When taking treatment there is need for clear information on how you are supposed to take the pills. Like myself on the first time even if you are to ask my father here, I was just given pills, there were so many and was told that you are supposed to take them same time everyday, you take them same time everyday. I didnt know that what I was told about the same time...I was supposed to drink all the pills once or 15 or 12 at the same time, I would take some in the morning, afternoon and some in the evening. Do you get that. What I know is that I was suppose to take them at 8 am, I woul eat my porridge then take my pils, if its lunch time I would take my pills at 12, after supper I would take them at 6. Isnt it same time if you are to look at it? They said you should take hem at the same time, I took almost a month then met with Phebbie here because there they dont inject during the weekend. We would then go to Poly for injections, Phebbie then said mama, how are you taking your pills, how many do you take? That is the moment whereby will be asking each other as patients. As patients we ask a lot and we would be sharing ideas. Phebbie then said, the way you are taking your pills mama is not the correct one......but that’s the way I was told...her mom works at the clinic there and she told her that you dont take them all at once, you chose whether you want to have them in the morning, afternoon or in the evening. She told me that you can have them in the afternoon or morning then spend some time doing exercises whilst the pills would be absorbed in the system. I don’t know how these pills make you feel when you take them, when its evening, they would disturb you sleep so you need to take them during the day so that when they disturb you it would be in the afternoon so when its night you can have a good sleep. I then said aah I wasnt aware about that then I changed and took them the time I wanted. I asked her the time she was taking hers and she told me, I was now taking the pills same time with her. I didnt have enough information . The people would act as if there would be afraid when giving you information....its as if they say, let me quickly tell you and leave you to go otherwise you would infect us with MDR. So old people where really taught like HIV and AIDS, people are taught this and that....people would get into it knowing but with MDRTB we know nothing, as for us we know the usual TB. Most people doesnt know about the other one. It really disturbs you because you wont have enough information.

What I see as important, the way they handle the patients of TB, they should give them hope that there is a future ahead. And also they should have a place where they say this place, it might be a small space...they should put a shade to cater for the TB patients and put them at a place designated for them not to throw them away, putting them where there is rubbish and say thats where you are supposed to be.

When taking treatment there is need for clear information on how you are supposed to take the pills. Like myself on the first time even if you are to ask my father here, I was just given pills, there were so many and was told that you are supposed to take them same time everyday, you take them same time everyday. I didnt know that what I was told about the same time...I was supposed to drink all the pills once or 15 or 12 at the same time, I would take some in the morning, afternoon and some in the evening. Do you get that. What I know is that I was suppose to take them at 8 am, I woul eat my porridge then take my pils, if its lunch time I would take my pills at 12, after supper I would take them at 6. Isnt it same time if you are to look at it? They said you should take hem at the same time, I took almost a month then met with Phebbie here because there they dont inject during the weekend. We would then go to Poly for injections, Phebbie then said mama, how are you taking your pills, how many do you drink....that moment where by will be asking each other as patients. As patients we ask a lot and we would be sharing ideas. Phebbie then said, the way you are taking your pils mama is not the correct one......but thats the way I was told...her mom works at the clinic there and she told her that you dont take them all at once, you chose whether you want to have them in the morning, afternoon or in the evening. she told me that you can have them in the afternoon or morning then spend some time doing exercises whilst the pills would be absolved in the system. I dont know how these pills make you feel when yooo take them, when its evening, they would disturb you sleep so you need to take them during the day so that when they disturb you it would be in the afternoon so when its night you can have a good sleep. I then said aah I wasnt aware about that then I changed and took them the time I wanted. I asked her the time she was taking hers and she told me, I was now taking the pills same time with her. I didnt have enough information . The people would act as if there would be afraid when giving you information....its as if they say, let me quickly tell you and leave you to go otherwise you would infect us with MDR. So old people where really taught like HIV and AIDS, people are taught this and that....people would get into it knowing but with MDRTB we know nothing, as for us we know the usual TB. Most people doesnt know about the other one. It really disturbs you because you wont have enough information.

Collins: Thank you so much for the information you have given us.

******************************************************************************

**Moses**

Collins: Like I mentioned before in this study we want to understand how you managed to stay on treatment despite the challenges that you may have come across. Do you have any questions?

Moses: No, I don’t have any questions. I am a married man aged 38. I am currently not working. I have two daughters of school going age.

Collins: Firstly I would want you to give me a brief background about yourself.

Collins: Firstly, we want to understand for how long have you been staying where you are now?

Moses: It’s been 15 years now.

Collins: Is this where you have been staying after diagnosed with TB?

Moses: I used to go out doing piece work jobs. But that’s where I stayed for long time.

Collins: Alright. Have you ever been diagnosed with TB before?

Moses: No. It’s my first time. I have never been diagnosed with TB before.

Collins: So, what do you know about Drug Resistant TB, anything that you have been told at the hospital either by nurses or you came across with?

Moses: Yeah. The nurses said that it is difficult to treat and the TB bacteria doesn’t respond to standard medication. Also if you are diagnosed and you know you have it, you are supposed to stay in a well-ventilated place so that you don’t infect others since the bacteria is infectious thereby increasing the risk to others around you. Since it is difficult to treat, it will be a huge burden to the family if we all get infected.

Collins: On the issue of how the disease is spread, what did the nurses say?

Moses: The nurses said that you are supposed to cough in your elbow mostly and if you are married you should desist from having sexual intercourse for a longer period of time so that you don’t infect your partner.

Collins: Alright. Let’s move on to the day when you started to feel unwell, what did you do to make sure you get medical help?

Moses: It’s ok. What I did when I started to feel sick? Firstly, I bought flue medication from a tuck-shop. Whilst taking the medication, I noticed that there was no change. I ended up taking other medications as well since I wasn’t sure what was exactly causing the sickness because sometimes I would have stomach aches. It got even worse and I decided to go private doctors. When I went there, some of them thought that it was malaria. They prescribed malaria treatment and I would go home and finish the course of malaria. Still, I realised it wasn’t getting any better since there was no change at all. When it became worse, I used to enjoy good, not sub-standard meals. I would vomit after having a meal and had stomach aches and diarrhoea. That’s when I went back to private doctors and was advised to have a chest X-ray. I think I had 7 Chest-X-rays done on me because they could not come out clearly. When I had the last one, that’s when they discovered that I had signs of TB and was supposed to go back to see the doctor. At that time, I had exhausted all the money I had while buying strong cough remedies such as Benylin cough syrup. But it was to no avail, there was no change at all. We had used all the monies that we had by the time I went back to the doctors at Wilkins who confirmed that it was TB. They gave me the treatment and requested me to have more tests. After the tests that’s when they discovered that it was MDR TB. I was really frightened to hear that. When I heard them saying it was difficult to treat, I said to myself, “A-agh, so this is the end of my life”.

Collins: Alright, whilst on that one, when you went for chest X-rays at private doctors, did you pay for them?

Moses: Yes, I used to pay. The chest X-rays were not free of charge. The examination charges were different from one private clinic to the other. We were also afraid to have those Chest X-rays because of the exorbitant charges that are involved. To make the matters worse, the X-rays didn’t show anything positive, so it was very difficult time for me.

Collins: Ok, so u paid for all the 7 chest X-rays?

Moses: Yes I paid for them all.

Collins: And all the consultation fees to see the doctor?

Moses: Yes, I paid all the consultation fees...some doctors require you to pay before you talk to him about your problem...he would then prescribe for you. The moment he starts to treat you, you pay again.

Collins: So, you started by consulting private doctors before going to public clinics?

Moses: I went to the clinic but they could not refer me to the hospital. They only gave me some medication according to the information I had provided to them. Like I said the situation I was in was bad that’s why I opted to go to private doctors so that I could get help quickly.

Collins: Alright, there is a time you went to Wilkins, you started your treatment and have your sputum taken.

Moses: Yes, I went to Wilkins.

Collins: So for how long have you been taking medication?

Moses: Have been taking wrong medication for a period of 2 months.

Collins: So, you were on wrong treatment for 2 months?

Moses: Yes. That’s when they changed my pills and said it’s because the ones that I was taking were not effective since it was MDRTB. I was given these 6 types of drugs and the moment I took them, I felt weak because you are supposed to have eaten before you take them...you get tired and nauseous.

Collins: Ok. You are informed that its MDR and you are given your results... What came to your mind after the notification?

Moses: Aaah, I just said to myself, “This is the end of my life, am I going to be well again?” They said the disease was difficult to respond to treatment. The fact that it’s difficult to treat, I thought it was incurable yet in reality, it takes time for one to get healed. It affected and disturbed me a lot as I thought it was the end of me.

Collins: When you were given those 6 types of drugs, have you ever thought that you were going to finish the whole course?

Moses: Not at all, I didn’t have any hope of finishing the therapy... I just thought I was going to leave all the drugs. The moment I took the tablets, it required a strong will for one to take them the following day because of the way they react in ones' body...but I later realised that it was because of insufficient food. If you don’t have enough food, it’s a huge challenge. If taken with an empty tummy, you won’t like them again the following day. So, if you find someone who is dedicated to monitor you like what my loving wife was doing, you persevere. I used to gulp *mahewu* and then take your pills or eat porridge and later on drink lots of water. It helped me quite a lot. But what really troubled me a lot was that the tablets, especially the first batch of tablets we were given on a weekly basis. I was then given 6 types of tablets which were given every 2 weeks. So transport was a challenge since I had to board a commuter omnibus. Sometimes I would hike two different commuter omni-bus to get to the clinic only to be told that the drugs were out of stock. They would then refer me to other hospital such as Beatrice Infectious Hospital or another clinic to check if the tablets are in stock. So, there will be huge challenges because there was a time when I used to go to hospital daily for a period of two months because they wanted to perform various tests on me. In most case clinic staff would say, “You came late, come back tomorrow”. Sometime they would give me two sputum collection mugs for evening and for the morning sputum and it meant I was supposed to have another trip to the clinic the following day. It made my life very miserable. Because at first you don’t have an understanding of what would be needed at the clinic but if you are used to it you would know what is needed every month. I would know what I am supposed to do monthly.

Collins: Ok, whom did you tell about your MDR results, did you go on your own to hospital or someone accompanied you?

Moses: I used to go with my wife.

Collins: Is she the one you usually go with?

Moses: Yes she is the one I go with.

Collins: We have skipped something, when you were diagnosed with TB, were you able to walk on your own or the fact that you go with your wife it’s because you couldn’t walk your own?

Moses: It was difficult for me to walk on my own. I couldn’t walk on my own. Sometimes we would hire a car because what happens is that, the legs will be very weak that’s why I was using a wheelchair or crutches. We would hire a vehicle to fetch me to the hospital and upon arrival, I would walk slowly to the hospital with the help of my wife and the driver of the hired car...the driver was very kind. We would go to the doctor’s room and see him, by this time we would be home already. When we use commuter omnibus that’s where we struggled a lot because I was too weak to walk properly...so I had to fetch out some monies for anything that is needed to be done...in-order to lessen the burden...for me to be carried on the back, it would be difficult...my wife would support me to walk. So there was nothing else we could do for our living...my wife stopped doing piece work jobs because she was looking after me.

Collins: Have you ever been carried on someone’s back because you couldn’t walk on your own?

Moses: No. But they would grab me from both sides.

Collins: So, apart from your wife, have you ever told anyone about your results?

Moses: Yes, we told our church peers, some relatives and friends.

Collins: How did they receive that?

Moses: Friends and colleagues accepted it but they distanced themselves from me for fear of contacting TB. Some of the church members received it well, they prayed with me together with some friends but they did not get near me for they did not want to get into the situation I was in. There were afraid because it’s difficult ... but as for my wife, she received it well and it gave me the strength to carry on.

Collins: Ok, so some of the friends kept a distance away from you?

Moses: Yes, they kept a distance away from me since they heard that it was difficult to cure...they would say, what if I get infected I would really struggle.

Collins: Tell us about how you were earning your living before you were diagnosed with TB. We want to know how did you earn a living as a family, where did you get your monies from as well as your wife?

Moses: Before I was diagnosed with TB, I would do part-time jobs. I used to search for part-time jobs and whenever I hear of any that pays better, I would quickly rush there because I was strong...and would use the money to meet some expenses. My wife used to do gardening and we would survive. At home we used to keep road runner chickens and it really helped us but we had a huge problem especially in the garden when all the plants wilted because ZESA switched off the electricity...where we stay we fetch water from the bottle that is powered by electricity so there was nowhere to get water. We would get water from our neighbours and that where we are getting it now...if you go there with your 2 or 3 buckets...if you get some for drinking and bathing, that’s enough...So the vegetables wilted during that period of sickness. We used to survive on it. Still there was no one to fully take care of the garden since my wife was caring for me. That time our chickens got affected by bird flu. We used to rely on chickens for our relish...if you get sick you don’t want to eat substandard foods. One needs to have good quality and nutritious foods but we had no money...we would sell some chickens then get money to buy sugar, cooking oil or rice. So that’s how we survived until we reached a point where she would sell some spanners...she would look around the house to find out if there is something else to sell so that I would have monies for bus fares. It affected my progress a lot since I couldn’t do piece work job even my wife couldn’t do anything. We would look at each other pondering where we would get mealie-meal and also sometimes we would get maize meal but the money to process it was nowhere to be found. It was difficult. So we would try harder and the situation for a moment seemed to get a little better. We no longer ate three meals like we used to do but reduced it to once or twice because of the circumstances that we were in. On the other side, our children were sent back home from school because of school fees arrears. My wife would go to school to have a meeting informing them that I wasn’t well and would pay the arrears in instalments when I get better. We still owe the school some monies even to this day. If we get the chance we will pay so that the children continue with their studies.

Collins: How many children do you have and did you came across a scenario whereby one would stay at home to help with household chores?

Moses: That’s what happened. The girl who is in Form 1 got to an extent that she would not go to school for a shorter period of time helping with household chores for 2 to 3 days or even a week and then she would go back and continue with her studies. I have 2 children, a boy and a girl. It was really difficult.

Collins: Thank you very much for continuing taking medication. You have seen the impact of TB in your life, what really made you to continue with medication otherwise you would have given up on it?

Moses: What made me to stay on treatment is because the nurses and doctors used to counsel and strengthen me. I got to understand how to properly take each of the medications, especially taking medication on time after food...I could notice that I was gaining weight and could see I was recovering...I could also feel that I was gaining strength. I was feeling better. So that strengthened me because there was a noticeable difference compared to the period before I started treatment. I could see change every time so that gave me the determination to finish the treatment.

Collins: Did you get help either cash or food from well-wishers?

Moses: I didn’t receive any help from anyone. There is some money that we were told is meant to help TB patients. I only got it once. It must be in June and it was USD 25. We converted it into bond notes [local currency]. At that time things were really difficult, it kind of helped me because I managed to buy some sugar, soap and cooking oil once. That’s the only time I managed to get the money. During other times and in the absence of well-wishers who can help with sugar in order for the children to have some tea...it’s difficult. It was difficult, I didn’t get any help from any organisation up to now.

Collins: Alright. Is there any type of food you were forbidden or encouraged to take by medical practitioners?

Moses: The doctors informed me to eat a lot of food that I managed to put on the table but mostly the nutritious ones. They also advised me not to take alcohol and as for me I don’t drink alcohol.

Collins: What about smoking, do you smoke?

Moses: No, I don’t.

Collins: Alright, on the issue of MDR patient incentives, did you face any challenges in registering and how long did you take to do that?

Moses: Yes, I faced some challenges in registering for the cash until a certain guy phoned me about the issue. He is the one who called me and said that he came across my name in the registers and I was supposed to get help, yeah the man helped me very much. It didn’t take me long and he also advised that it might take 2 to 3 months for me to get the money but I was never supposed to lose heart since it was definitely going to come. It surely came but as for now it has been long since I last received the money because it came once. I was really happy then and said to myself, ‘life is becoming easy for me’. At that time things became easier but was really difficult to be registered so that I could benefit from the money that is meant for MDR-TB patients.

Collins: Alright, tell us what do you think should be done on the day you are diagnosed with MDR TB? What should you do in-order for you to get registered?

Moses: Yes, they should register the patient immediately wherever MDR is detected either at the hospital or private doctor... he should add your name to the list because on your own...to look for helpers, yea h it’s difficult. You wouldn’t have any knowledge about that. As for me, I didn’t know that there is financial assistance. I just thought like any other disease it would be a man for himself but I have discovered it that one cannot manage on his own. In the absence of help, one is bound to have a miserable life. It was good to be registered the day the TB is detected. After registering if there are any programmes for MDR patients they then include you meaning you start to get help because if they say you are supposed to go to hospital after every 2 weeks, it would be very hard and there are lots of tests that need to be done. So one would need to board commuter bus or to hire a taxi because sometimes it’s difficult to board Kombis because you would need to be ferried from home to hospital and back. So if you have got some money and you don’t have savings, you won’t make it.

Collins: Thank you. There is a question we usually ask others, I don’t know if it applies to you. You are free to choose whether to answer it or not.

Moses: Yes

Collins: There are some other patients who are on ART, they are taking ARV pills. I assume you know what I am talking about right?

Moses: Yes

Collins: They will be also on TB treatment, have you ever been involved in such a situation where you will be taking either diabetes or any other tablets and the ones for TB, kindly comment.

Collins: They will be also on TB treatment, have you ever been involved in such a situation where you will be taking either diabetes or any other tablets and the ones for TB, kindly comment.

Moses: Yeah, I feel pity for people in that situation. The advantage that I had is that I was only diagnosed with TB. Because of the lump-sum of TB pills that I had....if I had diabetes or BP, it was going to be overwhelming. Luckily, I am taking only TB pills. Currently, I am taking TB drugs only but there are 6 types so it made my life easier...in the event that I had diabetes, it was going to be difficult. I feel sorry for my colleagues who are also taking other pills like Diabetes or ARVs.

Collins: What do you currently say about your future?

Moses: Yeah, I can say that my future looks bright if I regain my strength. I would like to make regain what lost, all the setbacks that I had...I would like to work very hard because uum it’s difficult to realise everything that I wanted to do ...was held back by this sickness up to this level...for me to surpass it like I wanted when I was healthy. I have to do something so that I finish building my home. I want my children to progress with their education or even have extra lessons whilst I prosper in everything that I will be doing...looking for projects. It’s a little bit difficult but with the strength that I am gaining...I feel if I get something to do I would be strong enough and would make sure I avoid getting infected by TB again...even my family, the whole country, loved ones, relatives and friends because it’s not easy.

Collins: Tell us what you have lost due to this sickness, it might be domestic animals or household goods

Moses: We started selling our household goods such as radio, TV, spanners…I am a driver. I have a Class 2 driver's license. We sold our spanners even chickens. I sold our roadrunners. They were many and some financed my trips to the clinic. Unfortunately, there was an outbreak and we lost all of them.

Collins: Approximately, how many chickens did you have?

Moses: I had a considerable number of road runners since I used to breed chickens of different sizes. I managed to sell at least 40. They died when the numbers had gone down to around 15. I was left with nothing. That was my source for family consumption because they were at least 90. Yes, they were many.

Collins: Currently, there is totally nothing left?

Moses: Not at all.

Collins: Are all your children going to school and no one is lagging behind?

Moses: They are all attending school. We had to beg the headmaster to allow them to learn. We wanted them to be enrolled on the BEAM programme but it seems one needs to have connections in order to get registered...whenever they are send away from school for not paying fees, my wife would immediately go to school to plead for them to be re-admitted. Like currently, when the schools opened, they were sent back on the opening day. Just yesterday, when we went to the hospital that’s when we went to negotiate with the headmaster advising him of the situation that I was in and he understood. They are currently at school.

Collins: Alright, my last question is...let’s say you are asked to improve the situation of people who are no TB treatment, what would you do or recommend

Moses: Yeah. Those patients on treatment need to be helped financially or with projects. When they do projects, the proceeds they get will be channelled to other projects like poultry for chicken and eggs. They can sell broilers and they can do gardening if the water is available....yeah their lives will improve. Since there is a lot of travelling involved, there is need for nutritious food so if one starts a project for survival, he would live well without any challenges. But if there is nothing to help patients…Uugm you get stressed and may die because it’s a bit of a challenge. You may be given treatment whilst you don’t have any food for consumption or you run out of your 2 week supply of tablets and you are supposed to collect some more whilst you don’t have bus fare. We face challenges in collecting the pills from the hospitals. You may get to the hospital only to be told that you were supposed to have come the previous day. Your treatment was taken by someone else or the doctors would advise you that they are reserving the treatment for one of their patients. It so happened that on one of the days, I was scheduled to collect my MDR tablets and the sister who booked the tablets advised me that the pills were already taken by a certain doctor so that he could give them to his patient. So it’s a challenge…., but if you have an income generating project, you can collect the tablets on scheduled dates and can live comfortably. If sugar runs out, you can easily buy it on time.

Collins: How did the healthcare workers treat you?

Moses: At the hospital…we faced a difficult situation. I have discovered that doctors are kind hearted. Doctors never looked down on people...nurses ended up running away from me. Even if I go to hospital or clinic today, I have to hide my face by looking down because the moment I get there and they notice that I am a DR-TB patient, they run away. They run away and even the way the treat us is inhumane. They look down upon DR-TB patients to an extent that sometimes I thought that maybe I was about to die because of the way they talk. “So you already infected your wife and children? Go away from here! Stand over there!''. They rudely chase you away from others to an extent that you can tell on your own that patient care is lacking in the hospitals...you will be in a hard situation. Even if you are about to finish the MDR therapy, it’s difficult. Your morale gets high when you get home and start sharing with your wife what would have happened. She would counsel you and tell you that it happens in life...You are about to finish the course, continue to be strong father of my children. The nurses are troublesome when it comes to looking down upon individuals, you can tell that there is no care at all but she is the one who is treating you. You will be disgusting to her.

Collins: Yeah, thank you very much for the information you have given us.

Moses: We also thank you.

**---------------------------------------------------------END------------------------------------------------**

**Peter**

41 year old male, not yet married. He relocated to live with his mother when he was diagnosed with DR-TB. He lives in a high density area. Socio-economic activities are vending and art. The suburb is known for theft, squalid living conditions and substance abuse. His girlfriend lives overseas and she sends him remittances during his treatment. Spent 30 months on treatment: 6 for DS-TB and 24 for DR-TB. Laboratory culture results came late to certify his treatment outcome. He endured 200 Kanamycin injections. DR-TB greatly affected his plans to emigrate to Australia on a pre-marriage visa. He lost at least USD 4000 during visa application which was denied on grounds of TB. At the time he started treatment, the cost of a pre-marriage visa was around USD 9000. When he finished treatment, the cost had gone up to USD 25000.

__________________________________________________________________________

Collins: Like I mentioned before in this study we want to understand the experiences that people on MDR-TB go through, and how they manage to stay on treatment despite the difficulties they may go through. So feel free to talk in even in slang, even jokes is it, feel free. So as we start I want to thank you for your time. We also want to congratulate you for finishing your treatment, we know it was difficult. So what we need from this study is to understand the various ways that made it possible for you to continue earning your living or livelihoods and also to manage your treatment, we know that the treatment was very long and too demanding. So all information collected here is reported anonymously and if you want you can give us a pseudo name that you want us to use in our reports. Then I want to remind you again that we are going to be recording this section but we will transcribe this recording then we will delete the audio files. Do you have any questions before we start since I have already explained?

Peter: Not at the moment.

Collins: Please tell me about yourself

Peter: I am a man aged 41 and am not married. I stay with my mother. I am not formally employed. I am an artist.

Collins: Alright

Collins: Alright, Please tell us how long you were on treatment?

Peter: Haa 2017, 2018, 2019 about 2 and half years.

Collins: You were on treatment for 2 and a half years?

Peter: Yes.

Collins: Then have you had TB before?

Peter: I never had TB before.

Collins: Since you were diagnosed with TB are you still living where you used to live or you once moved?

Peter: A-ah its like when I was diagnosed with TB I was in Chitungwiza…..

Collins: Okay, you were living in Chitungwiza?

Peter: Yes I was staying in Chitungwiza. But I was diagnosed at OK Mbare at a mobile clinic that one that moves around. So as soon as I started my treatment I moved back to the ghetto because it’s a short distance from my place to the clinic.

Collins: So what made you want to relocate and come back home?

Peter: It‘s like family support. Usually that is what made me want to… because I was staying alone. So for me to be able to take my treatment… when I take my medication I would get hungry and I was supposed to cook, I was supposed to… You know? Yeh, I couldn’t do all of this. So I realized that it was better for me to come back home to my roots, [*kune rukuvhute kuna amai ndiyamwe ndakarelaxer] shona proverb which means,* to come back for maternal care and support.

Collins: A-ah. Alright sharp. Maybe this might be a general question as we begin, what do you really understand about MDRTB?

Peter: I heard that it’s a multi-drug resistant TB which means it is resistant to some drugs so it needs heavy dosage which is strong so that it can be treated.

Collins: Uhh, how does it spread?

Peter: I think the way its spread its one and the same thing with the common TB, through the air, it’s an air borne disease. If I cough here and I am infected and you might catch it.

Collins: Alright.

Collins: Okay. Let’s move to the next question, what kind of support did you get so that we… what you would say that made you stay on MDRTB treatment? The kind of support which you ould say a-ah this helped me?

Peter: Uhmm it’s tricky, support in which way?

Collins: It can be emotional support, social support or material support?

Peter: Uhmm emotional support, Iike what I said before that I was open to my family when I was diagnosed with TB. And I thought it’s a normal what… it’s a common TB. So what happened is I went to Matapi Clinic and I was given treatment. Is it. I left my phone number, my address and everything and they called me after 2 days… Can you please come to the clinic? Then I went in the morning. When I got there I was not happy about the way they received me because she… you know when I got there I told them that I am so and so. You called me…

Collins: You called me.

Peter: Ok. You know…the person started to keep… the lady [nurse] started to keep a distance.

Collins: So this social distance started long back.

Peter: A-ah! So I asked myself, “What is happening?’ So she took some masks and she gave me in such a way that I could easily perceive stigma. ‘What is happening?’ I am confused… I started asking so that I could understand then she said “Your results came from the lab. As from now on you should always be wearing a mask”. Why should I be wearing a mask? I don’t know what is happening. And she said, “No the type of TB that you have is the most lethal and dangerous to people: it is called MDR. So wherever you are you must make sure that you are wearing a mask.”

Collins: Mask.

Peter: Ooh! Aah. I took it lightly then I put on my mask. I was accompanied by my friends and we were laughing about it saying, “Can you see whats happening here people have begun to treat me like an animal”. Then the sister in charge came and she calmed me down. She started to explain to me about MDR and common TB. Then when she was explaining she said, ‘Aa-ah! You see, most of the time people with MDR stop taking medication and the like so our policy here at our clinic is that you should come in the morning each and everyday… you take your medication here and you get your injection here then you go home”. Which was better because I didn’t have to take any medication home. My duty was to go and take my tablets and my injection and then go back home.

Collins: You said you disclosed that you had MDR-TB at home?

Peter: Yes… when I went back home I explained the process … I explained everything to them. Then my mum, my young brother, my sister… I don’t want to lie… these people gave me courage.

Collins: How?

Peter: Emotional support because they didn’t stigmatize me as like was done to me at the clinic. At the clinic they discriminated me to the extent that a-ah I was shocked that I had become dangerous within 2 minutes and 2 seconds [a very short space of time]. So my family assured me that such things do happen. They said, ‘We just want you to recover. Take your medicine at the time that they have instructed you until you finish’.

Collins:How about your friends when you were going back home with them did you do what you were doing before or they were showing that they were…?

Peter: These guys I grew up with them and they know me very well and nothing changed.

Collins: Nothing changed?

Peter: Uhmm.

Collins: A-ah alright good to hear. Now when you start having TB symptoms it might be coughing or weight loss is it, symptoms of TB… What did you do to treat the symptoms?

Peter: Honestly speaking I knew that you would start to have night sweats but I just didn’t take it seriously. I didn’t really take it seriously.

Collins: But you once experienced night sweats?

Peter: Yes I experienced it but I didn’t really take it seriously that there might be something wrong. Yes I lost a lot of weight… I lost weight, but because I stayed alone sometimes I didn’t really prepare food so that I could eat at my place… sometimes I would have two meals per day. For me to cook at home you know…. So I didn’t take it seriously that there was something happening to my body. I just thought that it was due to the environment I was living in and the situation that I was in. I started to take this seriously when I went to the mobile clinic.

Collins: You talked something about “situation”. Which situation are you talking about?

Peter: E-eh. My job.

Collins: Where you working or you were not working?

Peter: I was working, I was working. E-eh, Uhmm. I am a sculpture.

Collins: Ooh! Sculptures.

Peter: So I was the the leader there. Right. During that time I would supply…. I have a friend who is in the States [USA], so I used to supply the sculptures to the United States of America and Australlia. So there was a lot of work because of demand and different time zones, stress is triggered. Sometimes I had to work until 1am or 2am because it will be day time there.

Collins: So how long did it take for you from when you started to experience these signs until you were diagnosed with TB?

Peter: A-ah. About 4-5 months.

Collins: Alright. Did you go to any other places and try to seek treatment like at the pharmacy?

Peter: Not at all. Around that time is when Broncleer hit the streets. You see. (*Broncleer - commonly known as bronco is an addictive cough mixture which is abused by youths*).

Collins: Broncleer?

Peter: Yes. So as for me I needed Broncleer to sustain the demands of my work. So I was not serious about it.

Collins: What are the benefits of Broncleer?

Peter: I took it to get high [drunk], not for medical purposes or for anything else. No.

Collins: So that you would get drunk like what alcohol does?

Peter: I wasn’t taking it because I was coughing. No. Because I stopped drinking beer a long time ago. So through the environment like what I was saying the people I worked with ….that was their hobby. So I joined them.

Collins: Alright, alright so do you smoke?

Peter: Yes. I used to smoke.

Collins: When you got infected with TB…

Peter: Yes. I used to smoke, I would smoke even 2 packs.

Collins: 2 by 20 cigarettes?

Peter: Yes 40 pack.

Collins: Per day?

Peter: Yes. From morning till I get to sleep. I used to smoke a lot my friend.

Collins: So when did you quit smoking?

Peter: The moment I was diagnosed of TB.

Collins: When you were diagnosed…wasn’t it difficult for you to quit?

Peter: Yes it is difficult. It’s not an easy thing to just let go.

Collins: But you said you are… a-ah great. Okay so when that nurse told you that you had DRTB tell me… lets go back to that day and that scene. Can you visualize the nurse who informed you about the DRTB results and she gave you a mask. What what came into your mind??

Peter: A-ah…

Collins: We are on the scene, what can you see…

Peter: I just said this person is disrespectful.

Collins: [Laughing]

Peter: True. Because I didn’t know that there are different types of TB… I didn’t know. So I wasn’t happy when I saw the way she was responding to me. I said when I came here you gave me treatment to take home and I left and everything was okay between us…

Collins: Hoo you didn’t know?

Peter: Have I gone so bad today to the extent that a person can tell me to “wait over there”, to be discriminated like that you know it upset me. I don’t want to lie.

Collins: Did you think about… did you not think about issues of how your life is going to be or… think that maybe you will die?

Peter: I didn’t think that I was going to die. The only thing that bothered me was the issue of stigmatization that I experienced there.

Collins: Outside the household, did you disclose to other people apart from your friends, you said you told your family members and they accepted it?

Peter: Yes.

Collins: How about outside apart from your friends?

Peter: Outside apart from my friends?

Collins: Yes.

Peter: Yes. My workmates, the foreigners I told them.

Collins: Alright. So what did they do?

Peter: They said it’s a good decision that you have made to get treatment.

Collins: Alright before you were diagnosed of TB, what was your source of income before you were diagnosed with TB?

Peter: Before I was diagnosed?

Collins: Uhh.

Peter: Everything was okay.

Collins: I mean what did you do that gave you money, what we can call socio-economic life?

Peter: We had an NGO. It was all about helping artists, vulnerable artists who didn’t have access of getting enough income. So we would import raw materials from South Africa and give it to the artists. Right. So something that they would sell to us for USD0.50 we started to give the material to the artists at a price of USD $1 and the material was ours, because it was all about uplifting others so that they could look after their families. Then we would take the artifacts and export them to America. When profits from the sale of the artifacts would be wired back to Africa to buy raw materials and also pay the artists. So my socio-economic life...E-eh. Everything was good but as soon as I was diagnosed with TB, everything changed because you cannot be paid when you are not coming for… for work.

Collins: So even when you want to compare the money that you used to get before and what you were getting was there a major difference and how big was it?

Peter: Big… big difference. Haa it was big I cannot measure it.

Collins: Before how much did you get per month, an amount that you can say I woud not get anything below this amount then after being diagnosed with TB it reduced to how much roughly?

Peter: Before e-eh I would get a monthly income of about USD 200. After, thing got difficult to the extent that it was difficult to get even USD50.

Collins: Alright. So did you get to a point where you struggled to get food in your home?

Peter: I don’t want to lie. If I was living alone…. It was going to be very difficult. But coming back home to my mother…. My mother tried by all means…., what she didn’t want was…you know, like first thing in the morning when I wake up … my mother was so busy to the extent that she would brew 20 litres of *mahewu* because I loved the drink during that time.

Collins: You loved the drink.

Peter: It was like food and drink on my side because haa that medication is so painful. To the extent that my legs were swollen… a-ah…

Collins: Which part of the legs got swollen?

Peter: They were really swollen, these legs were swollen you see starting from here [knee] going down… They got so swollen that even this shoe wouldn’t fit. Uhh, the medication affected me. Now one of my ears does not hear properly.

Collins: You do not hear properly?

Peter: I do not hear properly even if I take an earphone and I put it in my ear then I increase the volume to the maximum, I won’t be hearing properly.

Collins: Alright. You touched on other things, what we call your experiences like your ear problem, what else did you experience during your MDRTB journey?

Peter: Haa I experienced a lot of things my friend. A lot. You see when I was in Matapi then they decided to transfer me to Mbare Poly because Mbare Poly is near to where I stayed. So when they transferred me a-ah I was frustrated my friend… I left Matapi in the morning but was attended to at Mbare Poly I think around after 1pm.

Collins: When you were transferred?

Peter: Uhh, I ended up complaining and shouting at the top of my voice while at the clinic. The sister in-charge came when she was informed that there was someone who was acting crazy. Then the sister incharge came and I said, ‘what kind of service is this? I came here in the morning…’ I just want to say… I have been given my papers [treatment booklets] to give you here so that you can start treating me at this place but that your service is very slow to the extent that it’s now lunch time and I haven’t been assisted. ‘What kind of service is this? It’s better for me to go back to Matapi because at Matapi I have no hustles. Each morning I get at Matapi I get my medication and my injection and I go back home. I don’t spend more than 15 minutes at the clinic. But now hunger has affected me because I have been here for a long time and there is no one to assist me. That is when they started running around and transferred me, then they gave me the… and then they accepted me in their… and I went home. So what was frustrating at Mbare was that each and every day I had to come for an injection… Now the way the procedure was done… we were not taken to a concealed place. There is an open area where there is a gazebo; that is where we would get our injections. There were three of us and we would go there and receive our injections. It’s an open area my friend not… [Sighs]. It’s an open space… To the extent that you will… you will be wanting to… to survive you know. You would just follow what they would be asking you to do but a-ah you know there is a certain level of privacy that is required my friend… There is need for some form of privacy. I can't pull down my trousers and get an injection while the other nurses are watching from one of the rooms. It doesn't … it is not appropriate you know. These are some the things that bothered me but there was nothing I could do.

Collins: There was nothing you could do.

Peter: Uhh.

Collins: Have you ever had the problem of taking your medication, did you just kept going to the hospital to take your medication or there are other times when you were offered to take the medication at home especially non-injection ones?

Peter: E-eh medication in the form of tablets, when I moved to Mbare there was no policy of taking the pills on site, they said go and take them at home.

Collins: Alright.

Peter: So I would first take my tablets and then go to the hospital in the morning and get my injection. I was just going for injections at the hospital.

Collins: Did you ever face any challenges collecting your pills?

Peter: It happened there was a big challenge when there was a shortage of tablets. They were not available, I think I was left with how many… very few which could not last 2 days… I went and asked because they give you a review date for collection of pills even a week before. So I would usually go now and then because of the injections… Then I said a-ah what about tablets and they said hii here tablets are in short supply. I had to be referred to Nazareth, and I went there the following morning and I got them. But I was given a few for about a short period of time, about 3 weeks after that things had normalized and medication was now available.

Collins: Alright, how about the issue of getting hungry… tell us about hunger after taking the tablets.

Peter: Haa there my friend I was… a-ah I was eating a lot haa even more than someone who takes marijuana a-ah. Early in the morning I would drink mahewu first thing because what happened is I would start to feel cramps in my stomach. You know how the heart beat is like you will be feeling it in your… in your stomach because you will be feeling hungry. But when I drank 2 glasses of maheu then it will calm down. But in less than 2 – 3 hours I need something again to eat. Haa it was a difficult time my brother. You know I got to a time when… you know sometimes you can be selective, like as for me haa I don’t like vegetables, but during that time I don’t want to lie to you I wasn’t selective of any food. My mother would cook the mutsine (black jack leaves) I would eat, I do not like it but I would eat, she would also cook muboora (pumpkin leaves) you know… A-ah I would eat, even my mother was surprised that a-ah is everything alright a-ah because haa it was a difficult time.

Collins: But which type of food did you really really like?

Peter: The type of that I really liked?

Collins: Yes the one that you craved for…

Peter: Fish, but without money, it cannot just appear miraculously on your table. It’s different from the black jack leaves you know e-eh even the pumpkin leaves which are readily available at home because my mother grows them in her garden. So its easier to just go in there take it and cook it. That is what sustained me but haa it was difficult my friend, very difficult, haa when I think about that time uhmm I feel the pain.

Collins: At the hospital did they suggest the kind of food you were supposed to eat and the kind of food you weren’t supposed to eat?

Peter: At the hospital they did not tell us anything. Their concern is are you taking your tablets, they did not tell anything about diet and the type of food that I am supposed to eat.

Collins: Alright.

Peter: It was tough, I know these are the services that they are supposed to be offering people at the hospitals but haa they don’t.

Collins: Alright let’s go back to this question, so your mother supported you is it? Did she face any other challenges that she ended up of selling some of her things or borrowing money?

Peter: You know as for my mother… I don’t really know but this stage could have been reached during the time because two and a half years is not a joke. So those are some of the things that my mother would not reveal to me that she is going through a lot because revealling that, as the first born child its obvious that it would affect me. I would end up feeling like I am a burden to my mother, do you understand what I am saying. So those things I know that somewhere somehow happened to my mother but she never showed me that was the situation.

Collins: Did you ever felt like you are burdening your mother even physically, emotionally, financially, did you ever think of that?

Peter: Financially yes, financially yes because when you your mother fetching the black jack leaves for you things will not be okay.

Collins: Alright.

Peter: Plus this disease affected me a lot.

Collins: Aright we are about to finish, you said you liked fish is it, was there a time when you told your mother that haa today I want fish and how did she react when u said that, maybe when you wanted these special types of food.

Peter: I used to do that once in a blue moon, once in a blue moon because as your mother you will be telling her how you will be feeling but at my age it’s something that I stopped because at home there are grand children you get it…

Collins: Uhh.

Peter: My young brother, his wife so it’s something that I tried to avoid you know.

Collins: Did you ever get to a point where you reduced lets say you used to have three meals but you ended up having only two meals or even one meal? Did you do it and noticed that a-ah things are probably getting harder?

Peter: One thing that… I didn’t reduce I do not want to lie to you. I wanted more but good thing about it what I have been telling you that I did not select what was put on the table by my mother.

Collins: Okay you did not select?

Peter: U-uh u-uh I did not select. If I see a plate of mushroom on the table I would eat, if I see anything placed on the… you know I want… I did not demand anything.

Collins: Uhh.

Peter: Whatever I was given by my mother was fine, because I got to a time where I would eat four meals a day. Mahewu in the morning, around 10-10 I was given porridge, in the afternoon Sadza, Sadza around 3PM, in the evening Sadza haa I was eating a lot my friend.

Collins: [Laughing] Alright, alright. There were cash transfers, money that you were supposed to be given to you when you started your treatment you would be registered so that you can receive the money that is supposed to be given to people who are on MDRTB $25 per month, did you receive that money?

Peter: Uhmm what you are saying is new to me. For two and a half years I never heard of such a thing that there is money [chuckles] that is supposed to be given to people.

Collins: So even at the clinic didn’t they tell you?

Peter: No they didn’t mention it at all. Plus as MDR patients we were being isolated. Even when we got to the clinic we were being told to isolate… Those with common TB were served first but initially the sister-in-charge had told us that we were to be served first. But some of the times we had to wait for others to finish first….to wait for about 45 minutes to an hour then they would come and administer our injections then we would leave. So what you saying about money it’s a brand new thing that you are telling me.

Collins: So what do you think should be done so as to fix such things?

Peter: I think there should be systems in place and follow ups should be done. Uhh because you see like for me I argued with more than people at Mbare polyclinic more than 5 times like when I come for injections this week…We knew each other very well with the nurse who was there and was supposed to administer my injections the week that followed but that nurse did not come to work because she had been transferred and there was a new nurse. I had to start explaining to her all over again. E-eh those are some of the things that disappointed me. I was always asked about the whereabout of my documents, and I would say, “Aa-ah so you people you are telling me that I am just coming to collect… one of the nurses accused me that I was coming to steal tablets and I said iih seriously can I come from home to steal tablets? Go and ask your accountant they know me a lot. Why is the information not in the system so that you access it automatically and you will be able to know that a-ah a-ah here we have this patient… Or when you are changing shifts at least you should give each other feedback… Here we have this number of MDR clients isn’t it so that we do not have to explain the same thing over and over again.

Collins: Alright, there is a certain question that I would like to ask you but you decide whether you want to respond to it. There are others who go on TB treatment but still they will be on ART, that is what I wanted to ask that if you were in that situation how was it that you were on TB treatment as well as on ART?

Peter: The good thing about it is what happened to me before they took a scan chest X-ray, they first did an HIV test and fortunately I was negative. So they did not put me on ART, I was only dealing with TB treatment.

Collins: Haa its okay so it does not apply to you is it.

Peter: Uhh.

Collins: Alright, I think we are left with only three questions, but now I want to here your own opinion. So what do you think your future is going to be like now that you recovered congratulations? But this question applied to people who are still on treatment but let me just ask you e-eh it’s the second last question. E-eh you said TB affected you especially financially is it? But generally in terms if your life how did MDRTB affect you?

Peter: Greatly. It affected me greatly…..because I was supposed to travel out of this country and go to stay in Australia. But I couldn’t do it because of MDR. If it was a common TB I know that I was just going to do 6 months and my things would be fine but it took me two and a half years.

Collins: Alright you mean you could have been treated in six months.What happened?

Peter: Time.Time factor and where I wanted to go, they want these tests to be done. I had to do three more chest X-rays for them so that they could be able to give me the VISA that I wanted. So haa everything was shattered, everything did not go as planned because…

Collins: At this time you had done some other tests is it?

Peter: Yes I had.

Collins: You had paid for them?

Peter: Uhh.

Collins: Then it failed because of TB?

Peter: Yes because of TB, all other procedure were okay, I had gone through them and using a lot money… And I did them in South Africa, it was frustrating because I had to travel to South Africa to have those tests done. I think I paid around 3000 to 4000 including travel and accomodation.

Collins: USD?

Peter: Rands. In South Africa they do not deal with… they only deal with Rands so this side it’s obvious that if you have Zimbabwe dollars you have to buy Rands, paying for transport and accommodation whilst these tests are done. Those things cost a lot of money.

Collins: So you could have been in Australia now?

Peter: If I have not been diagnosed with TB haa maybe we probably wouldn’t have met, we wouldn’t have seen each other.

Collins: So is there another area that you feel was affected, you talked about your finance and about these opportunities?

Peter: I think the after effects.

Collins: Alright, what do you mean?

Peter: The after effects of medication, because when I… my left ear started, I felt like l am hearing hollow sounds like huuu… I took it lightly then I asked the doctor and he said what is really happening, and l said haa I don’t understand what is happening with my ear seems like its closing. And he said alright lets wait and see for a month. I started to feel the other ear feeling the same…

Collins: The right ear?

Peter: The right ear, so at that time when you are talking to me i didn’t hear anything. I would then look at your lips, movement of your lips so that I know what you will be saying. Then I went to see the doctor on the next review date, doctor say hii we are reducing your dosage.

Collins: Of the injection?

Peter: Of the injection because I think it… was it 1gram, I am not sure…

Collins: Yes 1000mg…

Peter: Yes that is the dosage I that I got…1 gram. Yes that is the dosage that I got so it was reduced a bit but even now I still have problem with my left ear its nolonger hearing properly.

Collins: Anything else, how is your chest? Are you able to work again like you used to?

Peter: Yes I am working but my wish is I would to go again for another X-ray…

Collins: You want to go for another X-ray?

Peter: Because… because you see, what I noticed usually our local clinics they will be doing sputum sample collection and they have a lab on site. They don’t go deep, there is an incident that I saw someone that I used to be in love with, she passed away… through the fact that she would come to the clinic and they would collect a sputum sample and they couldn’t find anything and they will tell her that she doesn’t have TB, go home. But that person was already infected. When they found out haa the person was aready very sick. She didn’t last even a week on medication.

Collins: She did not last even a week?

Peter: She passed on.

Collins: Alright, let’s say if you wanted to improve the situation of MDRTB patients…what would you say should be done?

Peter: You see, if everything was okay, I heard that sputum samples are transported to Bulawayo… It is very far my friend. When they go there and then they do the tests, it takes time for the results to come back. Because of that I have… because when I went for the review, when they called me for review every month sometimes I would go without aresults but the doctor would tell me that whenever I come for review I should come with results… Why, because the results would not be back from Bulawayo, they would come later probably after two months. So things like that I think there is need to look into that , and here in Harare there should be at least a facility with labs which are able to test MDR sputum sample. So that at least people can be assisted. On medication everything is okay you get it earlier but there are other things which can be considered to see if there is any progress… Is it, there is a situation whereby I might continue getting injections but I would be already negative. But I will keep on getting injections because we do not know my results. So I think there is need to investigate that especially here in Harare there should be a lab.

Collins: Is there anything else that you would recommend?

Peter: And also what I have been suggesting that chest X-ray I think it’s fastest on testing people with TB.

Peter: Sputum sample testing takes time. Because as for me I got tested once and it didn’t take 2 or 3 times. There and there the doctor said to me haa my brother u-uh u-uh you are not well you need to be on treatment. I think that’s all. And also what you were saying if you could put policies that at least if people are supposed to be… the patients are supposed to be getting something because things are hard out there. Some of us we are in informal trading. If you do not go for work everything will also be on hold. Sometimes you would want to be consistent on your medication but it will force you to stop taking your medication and go for work because you can’t go on the street when you have taken your medication and if I am hungry I cannot go on the streets and work…

Collins: When you have taken your medication you can’t stand on the street?

Peter: Because it’s too strong (the medication), it’s very strong and you need enough time to rest.

Collins: Alright.

Peter: That medication requires you to have enough time to rest, it will be working inside the body. So if you do not have money and you want sustain your family and children haa it forces… the situation sometimes forces you to skip your medication, but it will affect your health.

Collins: Alright, haa do you still have something to say?

Peter: Haa I do not have a lot of things to say, but I just hope that if their systems are improved at least people will be helped.

Collins: Haa thank you so much we really value your time and contributions. You have told us a lot of things that we didn’t know and impact especially these things that you have been telling us we do not know about it in our medical field. We have finished our discussion, I think I can take 1 or 2 more questions that you might want to ask before we end?

Peter: Haa nothing much but at least when there are platforms like this haa as for me I am someone who wants to know a lot of things.

**END.**

**Ruth**

**CT**: Firstly, may you please tell us about yourself? How old are you and who are you living with?

**Ruth:** I am a 29 year old single mother of 2 children. The elder child is of school going age. I am currently living with my parents in their house. I am not working at the moment.

**CT**: How long have you been on treatment?

**Ruth**: I think I took 2 years

**CT**: If you still remember the exact month from such a time to such a time.

**Ruth**: Uum… I can’t remember the exact date now but I think I took at least 2 years because I firstly finished the TB course whereby I was taking pills...when I finished it thinking that I completed the treatment that’s when they started me on injections and I started all over again. But I was injected for 4 months only and I discontinued because I developed ear problems. I could not hear properly so when I advised them, that’s when they stopped the injections.

**CT:** Alright, so how long have you been staying here?

**Ruth**: I have stayed here for at least 4 years.

**CT:** Alright, so ever since you were diagnosed with TB, where you receiving treatment from here?

**Ruth**: Yes I was coming from this place

**CT:** Did they tell you about MDR-TB at the clinic? What did they say?

**Ruth**: They were saying this type of TB is curable but it can only be cured if you religiously take the treatment because if you skip the treatment...it is difficult to overcome it....for one to be fully treated, it needs one to persevere and stay on treatment. They were encouraging us that the disease can be treated but it needs one to persevere and continue with the therapy.

**CT:** What did they say about how the disease is spread?

**Ruth**: E-eh….they said the disease is easily spread. So we used to wear these masks before the introduction of masks for coronavirus when going to the hospital even when at home to protect those we stay with...because they said the bacteria spreads in the air so to protect others we were supposed to put on a mask.

**CT:** So you started wearing face masks before the coronavirus pandemic?

**Ruth**: Yes.

**CT:** So how was it like during that time, how did you feel those days?

**Ruth**: Aagh, I felt out of place. I can’t explain the feeling when you get to the hospital, you would be the only ones wearing a face mask....there were not common that time...I just don’t know....I just felt out of place. It was like I am different from other humans with a very serious disease.

**CT:** Alright, let’s go back. You mentioned that you once had TB. When you started to feel unwell, maybe you were coughing, maybe you had night sweats…What did you do till the TB was detected?

**Ruth**: Aagh. It troubled me a lot. It took a long time for the TB to be detected. Because at first, I would vomit after consuming food, after eating I would throw up that’s when I decided to go to hospital and informed them that I was throwing up after eating and they advised me to go for testing. I was then tested and they started me on ART and I thought I am now recovering but what disturbed me is that I got worse. It so happened that during the night I would drench my blankets with sweat, I would sweat profusely and when I eat I would vomit. There was a time when I shivered very much and what troubled me a lot was that the sickness continues yet the sickness has been detected, why is the sickness not resolving? Why am I losing weight daily? That’s when I went back to the same hospital and to the same people who had counselled me and I told them that since that time...I haven’t gained weight, I don’t know what’s causing this and they said, “Maybe you have TB. Let us test you for TB”. They took me and they tested me for TB. When the results came, I was negative but I continued to be unwell....was always sleeping, shivering and the moment I ate, I would vomit and had lost weight remarkably. They told me that I was TB negative and I was shocked to hear that I did not have TB. They asked me if I was taking my pills correctly because I was supposed to take them same time every day. They asked me to go home and follow what they had just told me that if I take them at 8 am I should continue to have them at 8 am. The moment I followed their advice I became even worse and had no power to walk at all.... That is when I went back to the hospital and said I do not know what is happening. I am getting worse. Around that time, I had difficulties in walking and my strength was gone. Wherever I was seated I would shiver and they tested me for TB again. They called me when the results came and told me that it was TB and I was initiated on that first treatment but there was no change. I just had a little improvement but continued to be sick. When I had completed the treatment, I was then told that I was supposed to be injected. But the moment I started that treatment... it took…it took me less than a month because I started to have problems with my legs...had pain in my joints....had no power so they used to sit me down because I couldn’t sit on my own...I had to ask someone to help me sit and when I needed to stand up I had to ask someone again to pull me up. But the moment I started taking injections, I was amazed one day when I was sitting on the sofa and I just tried to stand up ....I held onto the edge of the sofa and I realised that I was managing on my own. It’s something that took me a long time to do that to an extent that people knew that I could not wake up from my bed on my own, someone had to come and pull me up but the moment I started having injections, it didn’t take me time. Within a month I discovered that I was beginning to gain strength in my legs…was now able to do household chores like scrubbing the house because my legs were now bending unlike before...had problems with my joints.

**CT:** Alright, in order to get medical help, were you only going to poly clinics or you visited private doctors also?

**Ruth**: Aagh, No. I didn’t go to private doctors, I went to poly clinics only.

**CT:** Hmm, thanks for that, so how long did it take from the day you noticed the symptoms and diagnosed of the first TB? From the rough estimate, how many months or weeks were they?

**Ruth:** For me to recover?

**CT**: No. For the TB to be detected.

**Ruth**: Aa-ah. I think it’s 6 to 7 months before it was detected and was sick then. If I had continued like that aah it was going to be something else.

**CT**: Alright. Let’s go back to the day when the TB was detected, at first you were told that you were TB negative, the second time you are informed of the TB positive results, what came to your mind on that day?

**Ruth**: I received it well because what I had been diagnosed with at first was huge, it frightened and shocked me. So the moment I was informed of the TB results, the nurses counselled me nicely and I learnt that one has to accept everything that life brings because that’s the reality. If you don’t accept it then you end up dying because you would be denying reality. You have to accept it. So I accepted it because what was diagnosed at first was very scary so I said to myself this TB is curable if I take the treatment it can be cured. So there was no need to die in denial...I accepted it without any stress.

**CT:** Aaagh! That’s wonderful. So did you share the results with others or you just kept it as a secret?

**Ruth**: No. I shared the TB results with my relatives because people really wanted to know what had befallen me...I was bedridden and those days that’s when my grandmother passed on when my legs had serious joint pains. So my sickness drew the attention of everyone who was at the funeral. Whenever I wanted to sit down people knew that I was needed to be sat down, to be lifted up. So I realised there was need to let the people know what had befallen me. They were curious to hear about it because I was really sick....so when the TB was detected, I let them know what had happened.

**CT:** Alright, so how long have you been bedridden.... with the help of others to stand up?

**Ruth:** Aagh it was a long time I think it was at least 7 months...I can say the whole of the first treatment I wasn’t able to stand on my own...I managed to stand up the moment I commenced the injections.

**CT:** Before the first TB and this MDR was detected, how did you live because we want to see how your life was affected by this TB?

**Ruth:** Alright, before the first TB was detected, I used to plait hair. I was a hairdresser and a housewife. My husband was working in South Africa. So…TB affected me very much because even if I were to plait hair …..You know dandruffs….and….it’ not advisable for someone you once had TB. It affected my life very much because it affected my marriage. The moment I got unwell, my husband could not accept it. He lacked the will to help me. I just don’t know why; maybe he just thought I was going to die and he had nothing to do with me. Because the moment I got sick he ran away and went to South Africa. He never came back. He only came back recently when he heard that the mother of your child is now fit. That’s when he came back to see me. You know how men behave....but it affected my life....it affected my marriage and also my work, I am supposed to be busy plaiting hair [in a saloon] right now. If I decide to plait hair today, one of the clients may have too many dandruffs, and with this TB, I may get sick again. So it really affected me.

**CT:** Alright. You were now answering the question that I wanted to ask...how this TB affected you…from your marriage you mentioned that your children are not yet going to school, we want to hear your experiences on the monetary side, how were your experiences at the clinics?

**Ruth**: Aaagh. This one affected me in many things for example...my husband was tired of the situation and he ran away leaving me and my children relying on my parents. The clinic is quite a distance from my place. That’s the time when I was having problems with my legs...it wasn’t appropriate to ask money for combis from my mom everyday...it would be on their freewill so I really understood the situation I was in and that I was grown up. I didn’t ask bus fare whenever I went to the clinic; I would just keep quiet and wait for my mom to give me the money. On the days she didn’t give me, it did not bother me. The hospital was far away so it affected me a lot especially when I walk to the hospital because those who knew how fat I was and how skinny I had become and difficulties I had in walking. So it drew the attention of everybody...it does something in a person but I can’t really explain it. When you get to the hospital, you are supposed to put on a mask, sometimes you meet with people you know and they really wanted to know why I had to put on a mask each time I went to the clinic... We also faced some challenges at the hospital, we would get at the hospital in the morning around 8am only to be served after they are through with other patients or some other tasks. We would be seated outside, at the back of the clinic. I would have gone to the clinic on foot and would be tired and looking forward to my next meal when I get back home after walking to the clinic. You would be penniless and you are made to wait for a long time then you are injected and your stomach is empty. People wanted to know what was happening in my life. We experienced several challenges in this journey. Relations with close friends and some other relatives changed. Some relatives started to pretend as if I was about to die. They never consulted or included me in important matters of life...but would really want to thank my family for standing by me, they strengthened me and it made me strong. One other thing that really stressed me was my husband of 8 years and I bore him those two children….it’s not easy to be deserted after having getting to that level of a sickness. There is a certain lady I used to admire each moment we went for our injections. She would be accompanied by her husband and if it takes long for us to be attended, the husband would leave her and go to do some of his tasks then come back and accompany her home. I would now start to wonder why my husband never called me just to check on how I was. It stressed me lot and was always asking myself why this was happening to me but would really want to thank God. If it wasn’t for Him, I would have died. He would give me that strength to carry on with life. I would have that power to strengthen myself because sometimes I would pass by the shops and you would realise that people would be on stand still trying to get a glimpse of me. But I would want to thank God because I was never bothered by that. He gave me life, hope and strength to carry on with life. I was never worried about people, if I had done that I wouldn’t have recovered from DR-TB.

**CT:** Ok, there is a time you mentioned about being at the back of the clinic, what do you mean?

**Ruth:** It’s like when we got to the clinic people with TB were told that TB is spread in the air so we were told to put on masks. So they isolated us away from other patients. When you get at the clinic, you would have your card stamped after that, you would go and sit at the far back of the clinic. That’s where we were allowed to sit. We were not allowed inside the clinic because we would infect other patients with TB. So they isolated us. After they have finished whatever they would be doing, that’s when they would come to the back to attend to us. After that we would go back home.

**CT:** Ok, we want to know how the medication affected you.

**Ruth:** The treatment has got lots of pills. I think there were about 5 or 6 types of pills...Some you take 3, others 4 and the remaining you take 2. It’s a huge quantity so they would drain your strength especially if you don’t have enough food to eat. You need to eat a lot of food. Sometimes it is a challenge when you are being looked after...you are not supposed to demand that I need this and that but you just eat whatever is served on that particular day. So these pills used to make me feel very weak. Sometimes I would end up shivering because the pills need one to eat regularly but since I didn’t have any income, it was difficult to demand for more food, I just had to be content with what was provided. So the issue of not being able to buy some food stuffs and some of the medication because the pills are many and can get to around 16 that are to be consumed at once. You would have come back from the clinic to get your daily injection. So the treatment require one to have frequent meals. So when food is in short supply, it becomes a challenge because your body gets weak and you become dizzy.

**CT:** Alright, so what type of food did you really like and how did you get it?

Uum as for me I really liked *Mahewu* because I didn’t have any appetite, so most of the time I wanted mahewu and sadza made from rapoko because I heard that if one consumes ropoko sadza one gains strength faster. So that’s what I wanted. So sometimes when you don’t have money, you eat whatever is available. But there but there is a time when we got assisted by the state [Ministry of Health]...there are days we got money for 3 months from the state [Ministry] to cushion us. I would then go and buy rapoko meal, mahewu and bananas. After eating these, I would feel there was a change. I would see a big change because I would feel that if I eat and take my pills I would be fitter than when I haven’t had enough. I could realise the benefits of taking the pills. There are some instances where you are injected and you take pills at the same time...and when you get home you have some tea with bread only and you spend the rest of the day...you could feel as if the sickness coming back again.

**CT:** So what exactly can you point and say it made me to continue with my treatment otherwise I would have given up on it. It might be support that you got, help, what kind of help or anything that happened.

**Ruth:** As for me I can’t say I got supported and also I can’t say I lacked support because though my relatives showed concern, they don’t show that concern in terms of providing counselling. They were supposed to strengthen me....but my pillar of strength was God. Personally, God helped me a lot, am someone who grew up believing that God can solve my problems no matter how hard it was or big it was. I would call upon Him. So it’s Jehovah who helped me because when I started the treatment...when I was informed of the results I am someone who kept my strength...so when I kept on praying I would act as if nothing was troubling me at all. Like I said earlier on that I would walk to hospital whilst people would be looking at me, I wasn’t bothered that I was skinny and walking with difficulties. God gave me the strength and hope and I knew that everything was going to be okay. I couldn’t have made it without His strength. As for my family or people in general...I can’t really point exactly what they did, but God strengthened me.

**CT:** Ok, you once mentioned that you got incentives from the state, you mean the $25 or what?

**Ruth:** Yeah, the $25 ones

**CT:** How long did it take you to get registered, did you faced some challenges from the time that you were informed of the MDR for you to be registered?

**Ruth:** Uum. I am someone who didn’t know about the incentives. When we went to the clinic there was a certain man who came requesting for our names and other details so that we would be given some monies. So he wanted to send them for processing so that we would get help. As for me I didn’t know about it. It took time for us to get the money but it finally came...also for a short time.

**CT:** How did the money help you the moment you got it?

**Ruth:** It helped me a lot because I am someone who have kids and am not working. Their dad was not sending anything for their upkeep. It helped me a lot because that time I managed to buy some goodies in to lessen the burden on my parents who were taking care of me. I managed to buy some food stuffs, clothes for my children and school uniforms and books. It helped me a lot because I didn’t have any money and was not longer able to work or to do anything that give me money. I was once employed by Clean City where they collect garbage bins and the working environment has a lot of dust. The moment I got there, it didn’t take me a long time before I got sick. I think I only worked for just a week and started coughing again. I had that typical cough to such an extent that my all of workmates were worried about my health not knowing that I was previously diagnosed with TB. I later on quit the job. So this sickness affected me a lot. I can no longer get employed to do every other job; only those that pose less risk to my health.

**CT:** Alright. You mentioned that you were taking at least 16 pills, can you explain to us how it to take TB and ARV tablets.

**Ruth:** Aagh. It was a little bit difficult but I can’t say the ART pills disturbed anything since its only 1 pill. It’s only 1 pill so I would just take it as it is, the TB ones were too many and they cause dizziness. I didn’t have any problem with the ART one.

**CT**: Alright. Let’s say someone is diagnosed with TB today, what is it that you could recommend be done to him/her. You may reflect to what you have gone through yourself.

**Ruth:** The responsible people should first of all do background checks on the patient. Sometimes a patient would be lodging alone, going to work and if that person if diagnosed with TB, then it means he won’t be able to pay rent or buy some food stuffs. The mistake that I have discovered is that, when a TB patient goes to hospital, their [nurses] only concern is to treat the patient. You are giving him the treatment but the person was working and was able to go wherever he wanted. Now with the pills and the injection...you are supposed to go to the clinic for daily injections. You no longer have time to attend to your daily business. The injection itself causes dizziness. There is no way you can go back to work after getting the injection and a handful of tablets. The moment you go on DR-TB treatment, your career is destroyed. People are supposed to get help. It would be good to help patients with food hampers and also to verify their background, the lifestyle they used to live, who they lived with....A TB patient may be a family man, a bread winner having a wife and children and to make the matters worse, the wife won’t be working but relying on the husband. The family would look up to him and now that he is on treatment, no one would take care of the family. When the father goes out to hospital, the children would be expecting to receive some goodies when he comes back home. So the authorities must not just give medication without knowing the background of the patient otherwise to check where the patient is getting the food. Others would end up committing suicide due to overwhelming situations. Because there is no way you can go and collect TB treatment then you start to explain how hard your life is. It’s impossible. Even the nurses at clinics don’t want to hear that. If you have gone to collect the pills that’s it. You collect and go back home otherwise if you want to talk about something else, they would say maybe it’s the treatment that is making you insane. What I would advise the authorities is that if they want people to complete their medication they must research the on the lifestyle of those people before dispensing medication. They must check how these people are living, with who and their source of income. Other people are started on medication not that they will be very sick, so the moment he starts to take that medication and if it changes his lifestyle for the worse then he would just default taking the therapy leading to death. People are being killed by the situations that they face after taking the treatment, others won’t have monies for rent yet they would be lodging, food on the table yet these pills needs one to consume a lot, no income. Some of these patients might be staying alone, they just came to towns to look for work and they don’t have anyone to take care of them.

**CT:** Ok, the last question is how do you see yourself in the future?

I don’t know but I can tell that my future is bright, if it wasn’t for God who helped me...I can’t tell my expectations but I will just wait upon the Lord who does miracles. One day He might make a way for me unexpectedly. My working life was destroyed by this sickness but I have hope, I don’t know what my tomorrow holds for me but I have faith that it’s going to be bright. If it wasn’t bright, I wouldn’t be healed but the fact that I am healed it shows brightness. It’s not easy with my age to be infected with TB then go and injected like what I did. Most of them passed on long back because they could not handle it or do anything because of age, peer pressure from your peers you would be socialising with would be taking place but as for me I passed that stage. It’s not easy to wake up every day, bath knowing that you are going to the clinic to receive an injections; it’s not easy but I managed to pass that stage. My tomorrow looks bright even though I don’t know what’s going to take place but what I know is that God healed me.

**CT:** This one is optional, when your husband came back and saw you fit like this, what did he say?

**Ruth:** He is troubling me because he wants me back home and is asking for forgiveness for what he did.

**CT:** Many thanks for your time. I really appreciate. This is the end of our conversation. Do you have any questions?

**Ruth:** No. I don’t have any question.

******************************************************************************

**Sipho_M**

Collins: So thank you so much. As I said before I am Collins Timire we are doing this research to understand your experiences with MDR-TB treatment and how you managed to stay on treatment. Let’s start the interview. I would like to start by knowing when you were initiated on MDR treatment?

001: Can I speak in Shona?

Collins: Yes. You can switch if you want. If you want to explain in Ndebele it’s still okay.

001: I started the first treatment for 6 months…eehh…

Collins: Can you tell me about yourself including your family

001: I am a man aged 44 years and am living with my wife. I am no longer working because of TB.

Collins: Have you had both DS-TB and DR-TB?

001: Yes.

Collins: You were treated of DS and then you were started on DR-TB?

001: Yes.

Collins: Oh you have previously been treated of DS-TB?

001: Yes.

Collins: No. Let’s talk about the current DR-TB treatment.

001: Ok MDR, I was initiated on MDR treatment in June 2019.

Collins: June 2019? So you have been on treatment for more than 18 months?

001: It’s almost 2 years now. It’s going to be 2 years in June.

Collins: Alright, it’s ok. So when you were diagnosed of TB, where were you staying?

001: I was staying in Kezi, I was employed in Kezi.

Collins: So you have relocated?

001: Yes, I left Kezi when I was sick and came here to Brunapeg Hospital.

Collins: Ooh you were diagnosed of TB here?

001: Yes.

Collins: Alright, you were staying where you are staying at the moment?

001: Yes.

Collins: Alright. So you didn’t relocate again?

001: No.

Collins: You said you were staying in Kezi and also employed there. Alright we will talk about that later. I will ask about that later. So can we say the reason why you came here is because you were sick?

001: Yes.

Collins: Have you been told anything about what MDR-TB is?

001: No they didn’t tell me.

Collins: So do you understand what it is now? What do you know about MDR-TB?

001: Aaah, people sometimes say maybe I defaulted treatment what what...

Collins: Aha

001: The first treatment, took, it was supposed to be completed in 6 months. Back in 2003.

Collins: Uumm so did you complete it?

001: I completed it. From 2003 up to 2019 then I …

Collins: Alright. How is it transmitted? Did they tell you?

001: Yes they told me.

Collins: What did they say?

001: They said I should avoid gatherings, especially where there are children.

Collins: Alright. We are here now. When you were still in Kezi right, there is a time when you felt that you were no longer feeling well. Right? Maybe you were coughing; maybe you were sweating at night. We want to know what happened. Can you tell us what you did to try and get treated from the time you started feeling sick?

001: Eehh I started by feeling…I was vomiting after every meal, vomiting. That’s when I went to the clinic.

Collins: Where?

001: In Kezi.

Collins: Tell us everything that happened until you were diagnosed of TB.

001: When I went to Kezi aaah I had severe chest pains but I wasn’t coughing. I was vomiting and was experiencing a lot of pain but wasn’t getting any better, not getting any better. I started losing weight that’s when I came here. That is when I was diagnosed of TB.

Collins: Alright. So you started in Kezi they didn’t diagnose the TB?

001: No they didn’t.

Collins: Then you came here?

001: Yes

Collins: Tell me. Were there costs that you incurred when you were going to Kezi and when you came here?

001: Yah yah yes.

Collins: Tell us more.

001: I used to come here for ART refill. I hadn’t been transferred by then. When I fell sick I used to come here to collect my medication then go back using public transport. That became very difficult because I was sick that’s when I came here.

Collins: So you incurred transport costs?

001: Bus fare.

Collins: Did you pay in Kezi?

001: Yes. Not at the clinic, no at the clinic no.

Collins: So how much did you pay for transport to and fro.

001: R200 to and fro.

Collins: R200. Which is around 10 dollars, 10 US dollars?

001: It used to be R250 including food.

Collins: Ok including food? So how many times did you come here from Kezi?

001: Aaah I came here a lot of times. I last came here in…I can’t remember the exact dates.

Collins: Alright. But how much do you think you might have spent on all the times that you travelled?

001: Aaah it’s too much.

Collins: Could it be 5 times?

001: Yes it could be.

Collins: You came 7 times?

001: Yes.

Collins: Alright. We are here now. When you were diagnosed of TB here right, iihh the day you were diagnosed of MDR-TB what came into your mind when you were told that it’s MDR-TB?

001: Aaah I couldn’t comprehend what they meant by MDR. I only grasped that’s its TB since I have been treated of TB before. I told myself that I am healed I because I was once treated of TB. So if it’s TB that wanted to kill me, then I will be fine.

Collins: *[laughter]*… I am healed… Alright.

001: I gained my strength in knowing that it’s TB, Amen, Aaah, fine. I knew there is a block [building] in the middle with wards for people with various ailments where people are admitted whilst they wait for their actual diagnosis.

Collins: So you were once admitted?

001: There is a TB ward behind those buildings. When I was diagnosed I was admitted into the TB ward.

Collins: Oh so you were once admitted here?

001: Yes.

Collins: For how long?

001: Aaah I stayed for maybe 2 weeks in the wards at the middle blocks. My sputum sample had been sent to Plumtree for testing. Then the results came indicating that I had TB. That’s when I was transferred to the TB ward. I was admitted for four months.

Collins: 4 months?

001: Yes.

Collins: So they sent it to Plumtree which is 92 kilometers from here?

001: Yes they sent my sputum sample… [*interjection*]

Collins: …Ooh yes. Why were you admitted? Were you unable to walk or what? Why were you admitted?

001: What happened is the injections were just too many plus I did not have accommodation as a result I was admitted.

Collins: Had you started getting your injections by then because when you were diagnosed of TB… [*interjection*]

001: …I was too sick I hadn’t recovered…

Collins: Ooh you were sick…ok before you were initiated on treatment?

001: Yes. Initially I was admitted into the general ward then when my results came I was transferred to the TB ward.

Collins: Alright. So when they admitted you it was because you were bedridden?

001: Yes.

Collins: Alright. So how long did it take from the time you started feeling that you were sick up to the time you were diagnosed of TB?

001: Haaa maybe 2 months.

Collins: What about when you were told that its TB, its MDR-TB did you tell anyone? What did you do, did you tell anyone?

001: Aaah nurses are the ones who went home and told people there that I have been diagnosed with TB so I needed a place where I could stay in isolation. They said that there are children and other people I should be isolated but they could not find such a place that’s why I ended up being admitted.

Collins: Ooh they could not find it?

001: Yes.

Collins: Alright. So at home you were…, can you describe your living arrangements at home. Whom did you stay with at home?

001: Aaah I used to have…, I used to have my own family but my wife passed on. When I went to Kezi the homestead that I had left became dilapidated.

Collins: So whom were you staying by then?

001: When?

Collins: Isn’t you are saying your homestead got dilapidated, when you were diagnosed iihh you said they went home?

001: Ooh, to my relatives.

Collins: To your relatives, alright. How many people were you staying with?

001: Aaah…there were of them many maybe 10.

Collins: Alright thank you so much. We are…when they were told about your diagnosis, did they change their attitude towards you?

001: Yes they changed because…because when the nurses got home they said I have a very dangerous disease and I should be isolated. So they said aaah a person with this kind of disease when he gets here he will spread it, he is very dangerous. Then there were disagreements on where I had to stay. Now because I was too sick I was not able to build a shack or temporary structure for myself to stay in. You see. Aaah it became… then I realized that aaah there is a homestead that was left by my aunt close by. I rennovated the place then stayed there. I went to get my wife and we stayed there.

Collins: Ooh you remarried?

001: Yes.

Collins: So you stay with your wife at the moment?

001: We staying together.

Collins: Alright it’s ok. Alright, alright now we want to know what your source of livelihood was what your source of income was before you were diagnosed with TB when you were staying in Kezi?

001: In Kezi I was employed in the neighborhood. I was informally employed people in the community who paid me R800 per month, I got R800. My wife used to get a R1000, she was also employed close by.

Collins: You were paid 800 Rands?

001: Me, yes.

Collins: Your wife was getting a thousand so both of you were getting one thousand eight hundred?

001: Now we no longer have that thousand eight hundred we see it from others that there is something called money.

Collins: Oh how are the two of you surviving now?

001: Now we joined, we joined an Oxfam livelihood support programme. We get 10 kg of…isn’t Oxfam counts the number of people in a household. So our household has two people. We get 10 kg of maize.

Collins: So what else do you get?

001: Sugar beans and cooking oil.

Collins: In what quantities?

001: Less than 2 litres cooking oil then beans less than 2kg.

Collins: Beans, cooking oil and maize. Alright. We want to understand, when you were diagnosed with TB here right, tell us your experiences even with treatment.

001: Aaah what I experienced with TB here they no longer…they no longer treat us like they used to. Previously, when white people were still around even the food was good. Now it’s just the same as someone who goes to bed without eating anything. The treatment is too strong it increases your appetite. But the food is just too little. That was the main challenge, the issue of food.

Collins: What about the way they treat you?

001: As for me haaa I was like a matron because this hospital [*laughs*] at this hospital… [*interjection*]

Collins: …You were a matron?

001: With treatment I used to…treatment I was the one who used to mmmm, I was the only patient who was very knowledgeable and experienced with issues to do with TB treatment here. I was good at everything in terms of treatment. Unlike other patients who were difficult; others default; others have issues with treatment, I endured 108 injections and I’m taking my treatment for two years. There are lots of tablets like this…

Collins: Alright you talked about food, when you got home when you got home how did you get your food?

001: Aaah it was difficult to get food. We begged our relatives who had sympathy they gave me.

Collins: Uumm. Did you sell any household assets?

001: No I didn’t.

Collins: Alright. So what would you say if this and that was not available I wouldn’t have completed my treatment? If I hadn’t gotten this and that I wouldn’t have completed my treatment?

001: I left everything to God and the desire to complete the treatment but the biggest challenge was food. I did not have anyone who said come and get this or that. No one. I am just doing everything by myself. But I’m almost done now. Even if there are a few months left, I’m closer to where I’m going than where I came from.

Collins: Alright. What about food, which food do you prefer eating the most? Which you say aaah…

001: … This treatment makes you like things like meat or milk. With that I eat a lot. Even sugar beans. I also like sadza. Rice no [*laughs*] I like sadza the most.

Collins: So where you are staying now, are you having your meals as frequently as you used to or…if you used to have your meals twice or three times…

001: Aaah money, if you don’t have money where will the food come from? Nowhere.

Collins: I mean if you used to have three meals have you reduced the number of meals you have?

001: We can cook in the morning the two of us. We cook in the morning then in the evening then we have a meal in the morning then in the evening again.

Collins: People who are on MDR-TB treatment are supposed to get some money have you heard about it?

001: Yes some men came from Harare, whether they were coming from Harare or elsewhere I don’t know. They came aaah a long time ago around June last year. Aaah I didn’t get any money.

Collins: Did they ask for your Ecocash number?

001: Everything.

Collins: Did you give them?

001: Yes. We called someone in Harare to follow up. We didn’t get a positive response.

Collins: Nothing? Alright. You said you collect your ART medication here right. Tell me the experience of having to take ART and MDR-TB treatment at the same time. How was the experience for you?

001: Aaah for me everything is alright.

Collins: You didn’t face any challenges?

001: They are difficult…TB treatment is painful. It’s the one that has painful side effects. If I take the dose I experience painful side effects.

Collins: The other treatment does not have?

001: Yes it doesn’t have.

Collins: Alright, alright. So now how do you see your future?

001: Aaah I can’t say I have a future. I’m just waiting for the grave.

Collins: Why?

001: Aaah there is no future. Do you think there is a future? There is none. I can no longer perform physical activities as I used to I try to perform light tasks but I will be sick the next morning. No. It’s too much I have been unwell since 2003. This time it’s been…the next time it will even be worse.

Collins: Even worse? So you are saying if you try to perform some light tasks you will be sick the next morning you mean your body iihh … [*interjection*]

001: …It’s very weak.

Collins: You are not as strong as you used to be?

001: The reason why I haven’t recovered is because I don’t have anyone to assist me. If I was well provided for, I would have recovered. Every day you are always thinking we have run out of mealie-meal, we have run out of sugar yet there is nothing you can do? There is so much stress.You see.

Collins: So do you always have stress or the stress comes occasionally?

001: Aaah I am always stressed because the situation is hopeless.

Collins: Alright so if you could… [*interjection*]

001: …If I was someone else I would have stopped treatment because there is nothing I can do I am hopeless. I would have stopped long ago so that I die and leave all this.

Collins: So why did you continue with the treatment?

001: Aaah I just persevered so that I that I complete the treatment.

Collins: Uumm. Tell us what assistance would you recommend for people who are on MDR-TB treatment like you or those on TB treatment?

001: Aaah I don’t know how other people survive in their homes.

Collins: From your own experience.

001: You people if you are doing your job, you should assist us with food. Eehh that’s what you can help us with because if you are on this treatment you need enough food. Only that. If I haven’t eaten sadza I won’t take the dose because the side effects are painful because you will be hungry.

Collins: Alright. So in this current farming season did you… [*interjection*]

001: …in 2003 they used to give us food. 2003 they gave us food. When we came here we used to go to the kitchen to get food first before returning to our homes. They no longer do that. It’s the situation in the country.

Collins: So you want what used to be done in 2003 to be done again.

001: Haaa, maybe that would be very helpful in terms of food. When you come here you need to be given food before you go home so that you continue on treatment. Not all that other support. That’s why people default treatment, it’s because of lack of food.

Collins: Haaa thanks so much. Thank you. We are taking note of everything that you are saying. That’s what we have observed elsewhere that at least if people are assisted… [*interjection*]

001: …If there was a donor who would assist people with food it would go a long way.

Collins: How did you become one of the Oxfam beneficiaries? What happened for you to become a beneficiary?

001: The matron wrote a note for me. The note was very helpful, otherwise they hadn’t listed me. [*Flipping patient booklet to show the letter*]

Collins: [*reading a note from the patient booklet*] “Lack of family support and shortage of food. Forward mobile numbers to the Global Fund to assist them with funds. To link the client to social workers and NGOs to provide nutritional support and social support.” Alright. So the matron is the one who wrote this for you?

001: Yes, I showed it to the Oxfam field officers.

Collins: So you started benefitting soon after showing them? How long did it take for you to start benefitting?

001: No aaah it doesn’t take long. Maybe three months.

Collins: It took three months before you started benefiting? I mean from the time you showed them the note how long did it take for you to start benefitting?

001: Aaah it a took a long time. I have received the assistance for four times now.

Collins: When was the last time you received the assistance?

001: This month.

Collins: So you are still getting the assistance?

001: It’s coming to an end next month. They are stopping.

Collins: Why are they stopping?

001: That’s what they said.

Collins: How about the harvest in the fields?

001: The rains were too much.

Collins: Uumm. Aaah alright thank you so much for your time right. Thank you for your time.

*****************************-END-******************************************

**Spiwe**

Collins: Alright. Thank you for your time. In this study we want to understand how TB, MDR-TB affects your livelihoods. So all the information that will be collected here will be anonymous. Your name will not appear right? So I also want to remind you that we will be using this audio recorder. It doesn’t take pictures, it’s just the voice. I will be writing some notes. Is that OK with you?

Collins: May please tell me about yourself

Spiwe: I am a 20 year old lady. Am married and I live with my husband and my 1 year old daughter. We are currently staying with my parents. There are 7 of us. I am not employed

Collins: When were you initiated on treatment and how long have you been on MDR-TB treatment?

Spiwe: I could say since I was…when I left South Africa coming here I had…., when I left South Africa coming here I came in November. I had gone there in August. When I was in South Africa I used to go to the hospital for TB tests but they couldn’t diagnose it yet I started having the symptoms in October. My mother in law whom I used to stay with told me that night sweats, regular vomiting and loss of appetite are all symptoms of TB. In South Africa they cannot properly manage TB, it can only be managed here in Zimbabwe. With the hope that I will be treated she took me to a private doctor where she paid R2000 because it included pregnancy tests. We paid the money. He couldn’t find anything then I came back home on the 8^th^ of Novemeber and went to [Manama Mission hospital]. I got to Manama where I got treatment after being diagnosed with TB, but they said it’s MTB [DS-TB]. So I left after being told it was MTB [DS-TB] and came back home and started taking my treatment. I took it from November, December, January then when I was coming from collecting my resupply in February they called me back saying I was taking the wrong treatment, I have to be on a DR-TB treatment. They then changed my treatment to this current regimen. After that I came back and started to… when I started this treatment I started having sounds in my ears, my feet were sore and swollen…things like that.

Collins: Alright aahh. So you have just started your MDR-TB treatment?

Spiwe: Yes. In February 2020… [*Interjection*]

Collins: Now we are in March 2021.

Spiwe: Yes.

Collins: So you are now twelve… [*Interjection*]

Spiwe …and with this treatment, I’m not getting all the drugs. There is one which was always in short supply which is called Clofazamine. So I wasn’t getting all the drugs for my treatment. I started getting them later. They gave me a full course last month all along I was getting on an alternate basis.

Collins: Ummm.

Spiwe: And then I was given injections so that they could be administered at a nearby clinic because they wouldn’t admit me. I used to go by a scotch cart. I would skip one day and go on the next …like on an alternate basis. The nurses at that clinic who administered the injections didn’t treat me well like what they do at Manama. When she was giving me the shot she would…the isolation was too extreme like everytime I arrived I was instructed to go to some tree which was close to the toilets. Ug-h, the other patients would just stare at me inquisitively and that would hurt me so much as to why the isolation had to be that extreme. Why am I being treated like my kind of sickness would kill someone instantly if I came into contact with them. At one point I got hurt and my hand got swollen like this because the nurse would…after administering the injection when removing it she would hurt me she didn’t remove it properly. She would withdraw the injection in the manner similar to someone trying to cut off a piece of flesh and then sometimes she would literary stab me and sprinkle the dosage and some of the injection wouldn’t get into my body. I would even see some blood mixed with the medicines on the cotton swab that she would have used … [*Interjection*]

Collins: Um-mmmm…!

Spiwe: You see. When I finished I never had any problems as I was getting my treatment from Manama I never changed. I used to go to the nearest clinic for injections because Manama is far [20km away]. I wouldn’t manage to get transport to go there every day or afford to go there every other day so I used to go to Ruvuma because it is closer.

Collins: Alright ummm may you tell me what you know about MDR-TB? What you know about the disease.

Spiwe: They said it’s Rifampicin resistant. It can be cured or cannot be cured and it’s dangerous. That’s what they told me.

Collins: What did they say about how it’s transmitted?

Spiwe: The just said I should open windows of my bedroom when I wake up in the morning. Then with utensils I should have my own plate, have my own cup and my own spoon and use by myself even a towel I shouldn’t share things with anyone you see.

Collins: Alright, didn’t they tell what causes it?

Spiwe: No. They didn’t tell me.

Collins: I would like us to talk about where you started seeking TB treatment. I would like you to remember everything that happened from the time you started having TB symptoms up to the time you were diagnosed with TB.

Spiwe: I was in South Africa……Isn’t I went to South Africa in August, then in September that’s when I started feeling symptoms that indicated that I was no longer well. I used to have chills, when it’s cold I could feel the cold more I could shiver to the extent that I could feel it in my bones. I used to have night sweats, loose appetite in the afternoon if I force myself to eat I would vomit you see. So, I went to a clinic called FS which was close by and I… [*Interjection*]

Collins …In South Africa…?

Spiwe: Yes, in South Africa. They said they will test me but they couldn’t find anything. They tested for TB but couldn’t find it. They said come back next week and I went back and found different faces and they said I should pay. They said…fortunately the person that was at the window was a Zimbabwean who advised me to go outside where TB was being tested, I went there, got tested for the second time but nothing was found. My mother in law then took me to a big hospital called eehh… aahh I have forgotten the name aaah. Eeeh at the hospital and then….I have forgotten the name I will remember it there is a private doctor close by where I paid R2000 for a pregnancy test…[*Interjection*]

Collins: R2000?

Spiwe: Yes. They did a pregnancy test, tested for HIV and TB. They couldn’t find anything but my mother in law insisted that I had TB symptoms so she advised me to come back home. When I came back in November that’s when I went to Manama where I was diagnosed of TB, MTB. Then I took the treatment for three months, in February 2020 they advised me that I was taking the wrong treatment and told me I was an MDR patient.

Collins: Ummm. So when you came back from South Africa how many days did you stay before going to Manama?

Spiwe: I stayed for 3 days. I had money by that time, and then I went there.

Collins: Why didn’t you go to Manama immediately after arriving?

Spiwe: I was tired. I was exhausted because I arrived around 10 am spent the whole day and the next one at home and I went on the third day. I was tired the journey was too long for me and worse still I was not feeling well.

Collins: Would you tell me how long it took for you to be diagnosed with TB from the time you began feeling sick?

Spiwe: It was 3 months because it was September, October, November.

Collins: Isn’t it they gave you the wrong treatment at first …. [*Interjection*]. So all in all its six months. Alright eeh now you have been told that you have TB please tell me how you felt. What came into your mind?

Spiwe: When he brought my result slip and told me I had been diagnosed with TB. I wasn’t shocked because my mother was previously treated of TB. I told myself that it’s something that will pass and can be treated. You see. That’s how I took it.

Collins: Whom did you tell your results?

Spiwe: I had gone with my grandmother; the one that was seated here is the one I went with. Then when I came back here when relatives came to see me…isn’t they will be asking, “How did it go?” How did it go because I had also been given advice to go to the hospital. I could say on the second day when I had arrived people were saying go to the hospital you don’t look well. So everyone knew that I had gone to the hospital. When I came back after being diagnosed with TB they knew that that girl who is not feeling well is back and they came to see me. That is when I told them I have been diagnosed with TB and had started TB treatment.

Spiwe: Yes. She accompanied me because I was breastfeeding so when I came back they…isn’t they had that belief that it’s TB so they separated me from my daughter. I could walk by myself.

Collins: Ooh.

Spiwe: I was alright.

Collins: So when you told them how did your grandmother take it?

Spiwe: My grandmother comforted me saying I will be cured. Worse here at home my mother and father in law encouraged me to take my treatment and follow whatever instructions that I was given so that I could get cured.

Collins: People…?

Spiwe: Other people from outside the family that’s why I say 70% or 80% will be very different. I hear stories some of them are…for instance someone said I have HIV yet I know that I don’t…

Collins: Ummm.

Spiwe: At some point one of my relatives came in an inappropriate way. They came here…she came and asked to see my patient card in a way that is… [*Interjection*]

Collins: Did you show them…?

Spiwe: The way they asked….they ended up asking whether I was taking TB treatment only. So I got worried as to why I hadn’t been told at the hospital that if someone has TB they could also have HIV or contract it later or it’s possible that you could… I was frightened because I thought maybe I could have HIV after finishing my TB treatment yet they had told me that I don’t have it. How come? So I couldn’t understand that you see. Those are some of the challenges that I faced and I was disturbed.

Collins: What was your source of income before you were diagnosed of TB?

Spiwe: I used to sell some snacks and sweets. Those snacks that you pack into smaller packets but one packet used to cost R1 or 10 cents. But now that I have TB I can no longer sell things that I open and repackage because I can see that people out there…because people won’t understand that someone who is coughing is repackaging and sealing the packets it’s difficult to understand. So at the moment I am surviving by selling salt that is already sealed. I can just pick a packet and give it to someone.

Collins: Where do you sell from?

Spiwe: Here at home

Collins: How much did you earn per month?

Spiwe: With salt I get R100 and snacks I could get maybe R200 per month or R500 if there is business when schools are in session.

Collins: Is there a difference between the income that you used to earn before your diagnosis and now?

Spiwe: Now that I’m sick it’s rare for people to come and buy my stuff. Sometimes I struggle to get money for transport to Manama and I end up borrowing. If I don’t sell all my stuff I won’t be having money to repay the debt as a result I will be afraid of what the person that I owe would say you see.

Collins: Please tell me what you used to earn. Say the figure I want to see the difference. What you used to earn before diagnosis and what you are earning now?

Spiwe: I could earn about R500 from selling snacks and salt would give me R200. But now salt gives me maybe R100 at the end of the month because not everyone is free to come close to me some of them kind of…some of them are afraid of me you see. Not all people are comfortable coming close to me. So the whole thing becomes…

Collins: If we compare your life before you were diagnosed of TB and now, could we say there is a difference?

Spiwe: There is… there is a difference because before I was diagnosed of TB I could do a lot of things. I could stand… iihh now I cannot do a lot of physical activities. I could do this or that and be able to take care of myself you see. I could manage to earn something for myself in different ways but now it’s difficult because if I do something small whatever it could be, I easily get tired or for instance if I try to implement a certain plan or try to do something for survival I always ask myself what people think of me. People are, they are like isolating me… some of them are avoiding me. I have a lot of friends but when I got sick some of them comfort me over the phone but some of them avoid me such that I end up asking myself why this happened to me.

Collins: So these people that you are talking about, are they neighbours, relatives what kind of relationship do you have with them?

Spiwe: They are neighbours and other people in the community.

Collins: Ummm.

Spiwe: For instance you will be friends with someone like say okay let’s put some money together and buy something or I could say let’s put some money together for a loan scheme so we can buy something. I would share such ideas with people but now no one wants to do that with me or I could say chat with me on how I am doing or how I am surviving. You see people now isolate me that way.

Collins: Eeeh your relationship with your husband compared to…

Spiwe: At first he understood but when we got towards the end of last year I could say around October, November it became different. There is a very big difference because when I talked about going to the hospital if…isn’t it he is not employed and I’m not employed as well and I don’t have anything and sometimes I wouldn’t have sold any salt. He changes his mood as if I am bothering him. So that change… i-ihh that thing happens over and over again and tends to affect me and I get confused over what is about to happen. You see. I end up borrowing money by myself out of fear and I will be afraid that if I don’t sell anything where will I get the money? Then I get what…and some of the words are too heavy for someone like me who has TB. Such thoughts come into my mind.

Collins: You talked about how you were treated when you were being injected, I would like you to tell me about issues surrounding food. Things to do with your appetite, the kind of food that you liked then…. [*Interjection*]

Spiwe: At the beginning… [*Interjection*]

Collins: Has your appetite increased and how do you get your food?

Spiwe: At the beginning, isn’t it when you are on treatment obviously your appetite increases? So at the beginning I used to get dried fish and baked beans from my mother in law in South Africa and then some jam and other different things so that I could eat.You see? But now I no longer get the fish that I used to get so that I can have a variety of relish.

Collins: Ummmm.

Spiwe: I no longer get the variety. I now know that if we get better relish like meat if its finished I will have to make do with vegetables, dried vegetables. I won’t be having any choice but to wait until mid-month so that they can send me baked beans.

Collins: You have told me a lot of challenges that you have faced, that you are currently facing please tell me how you have managed to stay on treatment?

Spiwe: Its because I was told it can be cured, sometimes it cannot be cured but my biological mother told me…She was treated of TB so when I was diagnosed of TB I wasn’t….in my family they didn’t isolate me because I had TB. No or say TB kills. No, they accepted me and said I should adhere to my treatment; I will be cured because TB can be cured; if I don’t take your treatment I will die. So I also want to live. I also want to raise my daughter you see. I won’t stop taking my treatment and leave my child suffering.You see, it’s not good.

Collins: So is there anyone else who encouraged you besides your biological mother?

Spiwe: Even my mother in law when she heard that I had been diagnosed of TB she called and…she actually cried saying adhere to treatment, take your treatment and do not worry. Then in December she also came and she didn’t isolate me like making me use my own plates. No. She didn’t have that attitude of saying eat by yourself use your own utensils. No. She said, “Don’t worry.” But I’m the one who had to be careful because I didn’t want to put others at risk and have everyone affected, you see.

Collins: So besides the support you are getting form your mother and mother in law is there anything else that encouraged you?

Spiwe: I will be, I will be…isn’t in life you hear testimonies that so and so was cured so I also draw some encouragement that if I take my treatment I will also be a testimony. If I complete my treatment I will also be well why it would be impossible.

Collins: So when you were sick did you sell any household assets or property?

Spiwe: Yes. We sold two goats. We sold them under pressure because I wanted to go to Manama. Those goats had a value of around R600-R700 but we ended up selling them at R500 because I wanted to get what…money to go to the hospital. Because around that time donkeys were not there; it was during a drought season. So it was not possible for me not to go and get my treatment thus we had to sell the goats.

Collins: You talked about loss of hearing, when did that happen?

Spiwe: At the beginning of my treatment that when I could hear a sound in my ears and my feet were painful. When they were painful I didn’t go to the hospital, I told myself the only first aid I could do was to put my feet in cold water… [*Interjection*] they had a burning sensation. The sensation was as if I had put them in fire but after three days, no I can say after four days they were okay. My ears no my ears were now okay. My eyes, I also had a problem with my eyes when it was hot my sight will be blurry, it will be as if I’m blind or see things that are not real. When I woke up they used to be teary but they became fine.

Collins: How long did the problem with your feet last?

Spiwe: It could have lasted maybe a week.

Collins: What about your eyes?

Spiwe: My eye problem lasted maybe 2 weeks. But at the hospital they had told me that if you have problems with your eyesight for more than 2 weeks you should know that you have reacted to treatment. When I saw that I no longer had the problem at 2 weeks I knew that I was fine.

Collins: Have you ever received money that is given to MDR-TB patients?

Spiwe: No. I haven’t received it. They just said I should send my ID number, I sent it and nothing happened after that. I didn’t receive money, I didn’t receive any assistance.

Collins: When did you send you ID number?

Spiwe: Ummm they took my ID number in November 2020

Collins: Did you make any follow up, did you get any feedback?

Spiwe: No I didn’t. They just sent an SMS saying I should sent my ID urgently and that was the end of it. So I just told myself that aahh maybe since I stay where there are network problems, maybe I delayed in sending the number for the programme, but I was wondering what had happened as there was no feedback as days went by. I just concluded that maybe I delayed.

Collins: This is my last question. Let’s say you are now in charge of the welfare of TB patients what do you think can be done or what kind of assistance can be given to TB patients?

Spiwe: Like, I wish patients would get transport assistance when they travel to get their treatment or money for transport so that they get to the hospital on time because TB treatment is not the kind of treatment that one can skip doses. It’s not possible. You would feel that there is a problem; there is a mistake that I have made.You see. Even food, if patients could get food assistance; you as health workers would know which food is most appropriate for TB patients so that they can be healthy. You could assist them with food. They also need money if I don’t have money I could try and introduce something like having them assemble at a place like a school within their proximity on a given date. Like you would know that a central place so that they get their supplies like the system that is used with baby clinics. They would bring the medicines closer to the patients and give them because some won’t be having the funds to travel so health workers can be sponsored by the government to do that.

Spiwe: Personally I think this phase of TB will pass and my life will get back to normal where I can be able to provide for myself as I used to and enjoy life. That’s what I think… [*Interjection*]

Collins: You talked about laboratory results what were you saying?

Spiwe: Eeh that eeh I always have a problem when I get to Manama. They always tell me that my sputum results are not available. When I ask why it is every time when I come I don’t find my results they say they sent my sputum to Gwanda but they don’t know whether it got lost or it was received. On the latest sample this year they said…they called and were told that the sample was not received in Bulawayo but the person who had taken it there claimed that they submitted it yet they say they can’t find it. So I don’t know whether people who lose my sputum are the ones from Manama or it got lost in Bulawayo or Gwanda. I get confused because if I give them a sputum sample so that my treatment is changed and I do not get the results it means I stay on the same treatment. Maybe things will change you see. That’s something that really…

Collins: Alright please tell me more; you talked about the treatment that you get earlier on. Please tell me more about the treatment you get at Manama.

Spiwe: At Manama because I’m always late…I could say I always have transport challenges so when I get to the TB office late even at the laboratories I am treated well because they understand my challenges…they ask me why I’m late. But at places like the pharmacy they won’t attend to me you see. They say wait there we are going for lunch why did you come late because they would tell me I have lots of drugs which need a lot of time to sort out yet they will be hungry. But at the TB offices if Im working with the usual people they attend me in such a way that makes me feel comfortable and hopeful but if I go to other departments the treatment is different.

Collins: Alright thank you so much. **-END-**

**Tecla_F**

CT: Alright thank you for your time. As I mentioned before, we will be using this audio recorder. We want you to tell us your experiences during TB treatment until now, when you were initiated on treatment and how long you have been on TB treatment. I think as we start I would like to know…you don’t mention your name or phone number right. Ummm as we start I would like to know how old you are and where you stay…tell me about your family.

Tecla: I’m 30 years old and am married with one child. There are 6 of us where we stay.

CT: Alright. 6, how many of those earn an income?

Tecla: Just the 2 of us.

CT: What level of education did you complete?

Tecla: I completed form 4 my young sister is the one who is in university.

CT: What about employment do you get employed sometimes, are you employed?

Tecla: I’m not employed at the moment. Before I got sick I used to work in town.

CT: Alright we would like to know how long you have been on MDR-TB treatment.

Tecla: I have been on TB treatment for 20 months.

CT: When were you initiated on treatment?

Tecla: 23 July 2019

CT: Alright. I want to ask if you were told anything about MDR-TB at the clinic. What did the nurses tell you that you now know about MDR-TB?

Tecla: The nurses told me that if you are on TB treatment you are not supposed to cough with your mouth open, you should close your mouth when coughing. You are not supposed to spit on where other people walk on otherwise they will be infected. Then…with this type of TB they told me I’m not supposed to use…. during the time when I was sick I was not allowed to use the same utensils that others use or be close to children who are 5 years and below.

CT: Alright. How is MDR-TB transmitted?

Tecla: Uggh! The way they put it…they said it can be transmitted through coughing or through breathing. Plus spitting on the ground can also transmit it.

CT: Alright, thanks. Let’s look at the time when you felt that umn-n, I am no longer feeling well to the time you were diagnosed of TB. Maybe you can start by telling us what you felt and what you took to try and get yourself treated. Tell us where you went and the costs that you incurred until you were diagnosed of TB.

Tecla: When I started feeling sick, I had flue like symptoms but I would get treated like today and would get healed for 2 or 3 weeks and the flue would come back again. I would get treated again and the flue would be healed for 2, 3 weeks before it comes back again. It took a long time from January up to the 7^th^ of July when I was tested. I was tested and was diagnosed with TB. I had fever, chills and shortness of breath. Coughing came at a later stage but it was mostly fever, chills and shortness of breath.

CT: So when you felt like that from January to July right, did you go … were you going to hospitals or what? Where did you go to try and seek treatment?

Tecla: I went to private hospitals, that’s where I used to go and get treatment. I used to spend money on… paying for medicines. I went [to private hospitals] three times. On the first visit I paid R280. On the second visit I paid R130. On the third visit I paid R650. Those are the visits that I spent money.

CT: What was the money for?

Tecla: It was for medicines.

CT: Medicines? How much did you spend on consultations?

Tecla: Consultation was R250 but it excluded medicines because it was a private hospital.

CT: Is that where you were diagnosed of TB?

Tecla: Yes.

CT: Alright. So can we say it took about 6 months for you to be diagnosed of TB?

Tecla: Yes.

CT: What about the day you were diagnosed of TB, when you were told that you have TB what came into your mind?

Tecla: What got into my mind I … I was worried about how and where I had gotten it because I wasn’t using any drug. I had neither taken alcohol nor smoked cigarettes or even worked in an environment where there was dust. I was worried until I got counselling. They told me it could be family TB. Plus the TB affected my bones it could have been in the blood. That is what calmed me. But I was worried before I got counseling over why this had happened.

CT: Alright. They said it affected your bones?

Tecla: Yes.

CT: How did they discover that? Did they perform a scan at any point?

Tecla: They performed scans in South Africa.

CT: When you went to the private hospital?

Tecla: No. I was now going to the hospital by that time.

CT: Tell us more about the experiences at the hospital.

Tecla: At the hospital…Aaah. I didn’t pay anything at the hospital. They just took me for a scan. They tested my lungs they found out they were okay. Then they tested for this TB of bones and they said they have discovered TB of the bones.

CT: It’s called an MRI scan.

Tecla: Yes.

CT: So you didn’t pay at that time?

Tecla: I was now at a public hospital.

CT: Alright let that pass … [*noise from car passing by*]. So when you were diagnosed of TB whom did you share your results with? Did you tell anyone?

Tecla: When I was diagnosed of TB in South Africa my aunt, my mother’s young sister used to keep the results when we were moving around.

CT: Is she the one you were you staying with?

Tecla: Yes. When we were coming here we filed them in an envelope then I came over here. My mother is the one who kept them until we took them to Beitbridge Hospital.

CT: So you were diagnosed of TB in South Africa and then…..what happened until you came back to stay here?

Tecla: For me to come back here with the situation I was in….. It’s my mother and the rest of the family who said, “You never know what might happen. You might wake up in a worse situation or you might die and it will cost us lots of money, so its better you come home before your situation deteriorates further”. Plus at home I would be given better care than over there because I was supposed to complete my treatment in 6 months. Then someone told them that back in Zimbabwe things are better and there is better care. If it’s TB of the bones they will treat it until they see that I am totally healed.

CT: Then you came?

Tecla: Then they hired a vehicle to bring me here because I couldn’t board a bus or taxi with a lot of people.

CT: How much did it cost for the journey from South Africa?

Tecla: They paid them R1000 because we are related.

CT: Alright. When you got to Zimbabwe what did you do?

Tecla: When I was now in Zimbabwe I started treatment on the 3^rd^ of July. From then on I wasn’t able to walk because of weak joints and shortness of breath. So I had to hire a vehicle from July, August, September, October, November, and December. I hired a vehicle every day at R40 per day.

CT: Per day? Didn’t they conduct any tests over here? Tell us whether they…

Tecla: They conducted a test to…they wanted to check on my kidneys. They also did some on the side of my abdomen which was painful to check if my lungs were functioning properly. They then did some… blood tests so that they would know which blood group I belonged to. Those were some of the tests.

CT: Alright. So was this conducted at a private hospital or and tell us how much it cost.

Tecla: They were conducted at a private hospital. On the first occasion they paid USD 120 for a scan. Then the other time I paid USD 50. Then the other time I paid um-m I don’t remember how much I paid…..um-m either USD25 or USD 15. I can’t remember….I no longer have the receipt but it’s what’s written there. They wanted to know my blood group for a blood transfusion because my blood levels were very low.

CT: How much blood did they want to transfuse you with?

Tecla: I don’t remember how much it was. They had said those pints… they had said 2.

CT: What about TB, did they test for it?

Tecla: Here, yes they did. When I came back from South Africa they retested me.

CT: A sputum test?

Tecla: Yes they did but I had difficulties in producing sputum. They induced me so that I could produce a sputum. It was difficult to produce a sputum.

CT: So where did they find the TB?

Tecla: Ee-eh. I could say they found it on both sides. Both sides.

CT: Alright, alright. Mmmm let’s see how you used to earn your living before you were diagnosed of TB. In South Africa how did you survive, what was your source of income?

Tecla: In South Africa I used to work in an informal restaurant. That’s how I used to survive, take care of my child and assist my grandmother because she is the one who raised me. My mother used to stay elsewhere.

CT: Tell us how much you used to earn per month?

Tecla: I used to earn R2500 per month. I could only manage to send maybe R1500 home because in South Africa I had to buy food. I never used to pay for accommodation because my aunt, my mother’s young sister has a house. So in terms of rentals I was covered. I used to work so I could provide for people back home.

CT: What about now, can we say there has been a change in what you are earning now compared to what you used to earn in South Africa?

Tecla: Aaah! I see that a lot of things have changed because even if I want something, say I want this particular thing if I don’t have money I can’t get it because I’m not employed. In terms of school fees BEAM [government social support] came to my rescue. I used to worry and to depend on my mother to pay school fees for my child, and to give me some money for transport to go to the hospital every month.

CT: Alright. So tell us, alright thanks for that. Tell us about your experiences since you started your TB treatment journey be it at the hospital or challenges with money or anything else that you still remember to say mmmm that time. Your experiences. Even the drugs or their side effects, anything that you experienced during your treatment.

Tecla: I could say I wasn’t getting enough food at home because my mother was no longer employed. I used to get it here and there. At the hospital there were expenses also. Then there was a time when they made a mistake in administering an injection, I had problem with my leg which they treated until I was healed. Plus the money that was needed at the hospital, a lot of money was needed. Those were the challenges that I experienced.

CT: Alright. Have you ever been admitted?

Tecla: Yes I have been admitted 3 times here [in Zimbabwe].

CT: Tell us what happened. Were you paying when you were admitted, what was happening during the three times you were admitted?

Tecla: Yes on the first instance I was admitted for 10 days and we paid the required fee. On the second occasion money was needed for a blood transfusion that’s when I was admitted for 3 days whilst we were waiting for the blood. So when the blood was delivered a transfusion was conducted but if there was a delay in delivery we used to buy the blood. Then on the third occasion I was admitted for 5 days because my kidneys were no longer functioning properly and we paid for the admission and medicines.

CT: So when you were paying for admission and medicines can we say…when you were admitted each and every time were you admitted for one day and discharged the following day or. Can you enlighten us on that?

Tecla: No. I used to be admitted for 3 to 4 days, 3 to 4 days. The first time I was admitted for 10 days, I was admitted on the 13^th^ and discharged on the 23^rd^. I was admitted for 3 days on the second time. I can’t remember the date. On the third occasion I was admitted for 5 or 6 days.

CT: Have you had hearing problems?

Tecla: I completely lost my hearing at some point. Even now I have a problem that if somebody speaks I might not hear them. Plus I hear sounds in my ears.

CT: Did you tell them at the hospital and what did they say?

Tecla: I once told them I used to go for tests and they said I was okay but I hear sounds to the extent of not hearing anything. At the beginning they said it’s the side effects of the drugs.

CT: So tell us how much you spent on all this. What you spent on food and money for all those hospital bills ughmn the hospital admissions, you were spending money right? How did you get it, how did you get the money?

Tecla: My mother used to perform some menial jobs in the neighborhood. She used to clean houses and do laundry for people so that she could get some money to assist me. Then relatives who visited us used to assist us after seeing our situation but most of the times she used to perform some menial jobs.

CT: Alright. I want to hear if there were other sources of income besides menial jobs that you used to get money from like selling household assets or borrowing.

Tecla: There is a time when she borrowed from a taxi driver who used to drive me. She used to go for three months without paying the taxi driver whilst looking for the money. Then there is a time she sold a bed.

CT: How much did he charge per day?

Tecla: He charged R40 per day.

CT: Alright. So R40 multiplied by 3 months?

Tecla: Yes.

CT: Alright we are about to finish we are almost done. How did TB affect your life? If you remember how it affected your life, like say I lost this, this and that. How did it affect your life?

Tecla: Aaah. I could say it affected me because I lost my job which then hindered my progress in my plan for life. I was dumped by the person I have a child with whom I was married to because he had seen that I was sick and then he…he left me. Then I can’t get what I want whenever I want. I can say that’s how it affected me.

CT: Eeh did it affect the plans that you had? Are you comfortable sharing that I had planned this but because of TB I could not achieve that. Can you share? If you are comfortable sharing you can say haaa I expected to achieve that but because of TB if I failed to do that.

Tecla: Aaah I can say when I started treatment I had joined a loan scheme where we contributed R700 per month so that we could share the savings in December but I couldn’t continue. I stopped in June [after contributing R4200] when I became sick. I had started in January. I had hoped to buy a cow for my child. Plus I had other plans like starting a business for my young sister who was not employed so that when I hadn’t managed to send some money they would be cushioned. So I couldn’t continue the loan scheme.

CT: Did they pay you back the money that you had contributed?

Tecla: Yes. They paid me back what I had contributed up to June.

CT: Then what did you do, what did you spend the money on?

Tecla: Some of it on hospital bills, I helped my mother settle the taxi driver’s debt with some of it.

CT: So it was R700 multiplied by 6, which is R4200rands?

Tecla: Yes.

CT: What about the money that is given to TB patients explain to us if it has been helpful and how many times have you received it?

Tecla: I can say I have received it 3 times. I received 25 US dollars the first time but with the rates that were prevailing, it was in RTGS, bond notes [local currency]. If you converted it, it didn’t have much value; it no longer had the value it was supposed to have. The second disbursement was $75 US but it was converted again to RTGS. It was just enough to buy me a few groceries and I used the change for transport to the hospital. That’s the second one. The third disbursement was $ US 250. I managed to buy food, books and uniform for my child and a few other things that I have with the $ US 250. The third one because they had opened banks accounts for us we got it as hard currency but when we went to [the bank] to get it, they gave us $ US 235. I used the first and second disbursement for transport. I managed pay school fees, buy a uniform, books for my child and the food that we currently have with the last disbursement.

CT: Alright. Second last question. What do you think could be improved in terms of these funds? What do you think to say haaa they could do this and that with these funds? What do you want with these funds?

Tecla: We say if they give us the funds, like what they promised that they will be releasing the funds every three months they should do that in a timely manner because sometimes life will be very difficult for us such that there will be nowhere to turn to especially when it comes to food. Plus the treatment that we are taking needs us to have enough food so if we don’t have those funds we won’t be having any assistance. The problem is they fail to live up to their promise of releasing the funds at the stipulated dates as a result we suffer.

CT: If you look at your future now, you are left with 3 month of treatment right? How do you see it?

Tecla: If all goes well. I think I am now fit such that in 2, 3 months as I complete treatment; depending on how fit I am I will be able to search for a job so that I can take care of myself.

CT: Alright. Eehh last question. If you were asked to give advice on what can be done for people on TB treatment that you are on, what would you say can be done for them? If someone is on treatment I have seen that i-ih they have these challenges so do either one or two or three for them. What would you want?

Tecla: From what I experienced during TB treatment they should be able to…TB patients should be assisted with adequate food, then money for transport, then hospital bills or money for scans or everything than can be helpful to them. That’s my opinion especially food because if you are on TB treatment you need enough food.

CT: Alright. I have thought of another question about what you can specifically point out to say um-m if I didn’t have that or this person or that kind of assistance I would have stopped treatment or something might have happened with my life. What you can say um-m this and that or these people or this kind of assistance helped me, what is it?

Tecla: I can say what I experienced when I was receiving care; the nurses who were giving me treatment cared for me. They used to take time with me. When I was … when they saw that I was in a critical condition they used to support me. Then my mother is one person I can single out. If it wasn’t for her or if I had been cared for by someone else I would have died or I would have been in a different chapter. Haaa I would have stopped the treatment but she used to support me. She used to give me all her love.

CT: How long did she take care of you when you were unable to walk?

Tecla: My mother was my caregiver from July to February last year. July 2019 up to February 2020 that’s when my mother was taking care of me. Then I came over here to our rural home where there is my young sister who used to support me, cooking and doing laundry for me because I took a long time without being able to do my own laundry or nibble some *sadza* as my hands were too soft such that they couldn’t handle anything warm.

CT: When you say you took a long time without being able to nibble some sadza how long are we talking about? When you were being assisted you couldn’t even walk?

Tecla: I couldn’t do anything. That’s the period I said was from July 2019 to February 2020. I had difficulties in walking. Then March, April I could walk occasionally.

CT: What about people in the community were they gossiping or not?

Tecla: Because I was staying in town I wouldn’t say people were talking. No one used to see me I used to stay indoors. People in the community here in the rural areas were talking but that’s recent when I had already recovered. But I used to tell them that its part of life you can’t create gossip out of it or behave as if I got sick by choice.

CT: Did they get to the extent of wanting to distance themselves from you?

Tecla: Haaa they didn’t distance themselves but the issue is they start talking about me whenever they saw me like I became their topic of discussion wherever they were.

CT: Alright. Thank you so much my sister we really appreciate your time and what you have told us. Thank you so much for being patient with us especially when we delayed coming when you were waiting for us. Haaa if it was someone else you would find them gone right.

-END-
